# Artificial intelligence for lung cancer classification in cytology specimens: A systematic review and diagnostic test accuracy meta-analysis of benign–malignant diagnosis and ADC/SCC/SCLC subtyping

**DOI:** 10.1016/j.jpi.2026.100704

**Published:** 2026-08-03

**Authors:** Ayan Myssayev, Amin Tamadon, Nadiar M. Mussin, Reza Shirazi, Afshin Zare

**Affiliations:** a Vice-Rector for Strategic Development and International Cooperation, Astana Medical University, Astana, Kazakhstan; b Department of Natural Sciences, West Kazakhstan Marat Ospanov Medical University, Maresyev Street, Aktobe 030012, Kazakhstan; c Department of General Surgery, West Kazakhstan Marat Ospanov Medical University, Aktobe, Kazakhstan; d Department of Anatomy, School of Biomedical Sciences, Medicine and Health, UNSW Sydney, Sydney, NSW, Australia

**Keywords:** Artificial intelligence, Lung cancer, Cytology, Diagnostic test accuracy, Adenocarcinoma–squamous–small-cell carcinoma subtyping

## Abstract

**Background:**

Cytology specimens are essential for lung cancer diagnosis, yet distinguishing benign from malignant cells and subtyping adenocarcinoma, squamous cell carcinoma, and small-cell lung cancer remains challenging due to limited cellular material.

**Methods:**

We systematically searched seven databases for studies evaluating artificial intelligence-based lung cancer classification in cytology. After screening, 32 studies were included, of which 20 contributed 33 analyzable two-by-two data rows for diagnostic test accuracy meta-analysis. Outcomes were pooled using bivariate logit random-effects models. Summary receiver operating characteristic curves, Fagan nomograms, Deeks' test for publication bias, and quality assessments using QUADAS-2 and artificial intelligence-specific tools were performed.

**Results:**

For benign versus malignant diagnosis, 18 data rows encompassing 3464 observations yielded a pooled sensitivity of 89.3% (95% confidence interval, 84.1–92.9), a specificity of 88.5% (95% confidence interval, 83.2–92.3), and an area under the curve of 0.952. At a median prevalence of 58.2%, the post-test probability of malignancy after a positive artificial intelligence result was 91.2%, whereas a negative result yielded a post-test probability of 14.6%. For one-versus-rest subtyping among adenocarcinoma, squamous cell carcinoma, and small-cell lung cancer, 14 data rows comprising 2687 observations demonstrated a pooled sensitivity of 77.5%, specificity of 89.5%, and area under the curve of 0.901. Small-cell lung cancer showed the strongest performance (specificity 93.2%, area under the curve 0.949), whereas squamous cell carcinoma had lower sensitivity (67.0%). Substantial heterogeneity was observed across studies. Deeks' test suggested potential small-study effects, and the risk of bias was high in all included studies.

**Conclusion:**

Artificial intelligence-based cytology classification achieves high diagnostic accuracy for benign-malignant discrimination and shows promising but less mature performance for histological subtyping. Prospective external validation, standardized reporting, and clinically integrated human–artificial intelligence studies are required before routine clinical deployment.

## Background

Lung cancer remains one of the leading causes of cancer mortality worldwide, and accurate pathological classification is central to treatment selection, prognostication, and appropriate use of molecular and immune biomarker testing.^1^ Artificial intelligence (AI), particularly deep learning, is increasingly being investigated across the lung-cancer care pathway, but translation into routine practice remains limited by validation, bias, workflow, and clinical-integration barriers.^2^

Cytology specimens occupy a particularly important position in lung cancer diagnosis.^3^ In many patients with advanced or unresectable disease, small biopsies and cytology specimens may be the main available diagnostic material, and these limited samples must support morphological diagnosis, immunohistochemistry, and predictive biomarker testing.^4^ A systematic review of deep learning in lung-cancer pathology emphasized that histological and cytological materials are commonly used for diagnosis and that AI methods may assist lung-cancer diagnosis, subtyping, prognosis prediction, mutation-status characterization, and PD-L1 assessment.^5^

Despite the central clinical role of cytology specimens, particularly in patients with advanced or unresectable lung cancer for whom limited cytological material may constitute the principal or only available diagnostic specimen,^4^ AI-assisted cytology has received substantially less systematic attention than AI applications based on histopathology or radiological imaging.^6^ Consequently, the diagnostic performance, clinical applicability, methodological quality, and limitations of AI-based lung cancer classification using cytology specimens have not been comprehensively quantified in a dedicated diagnostic test accuracy (DTA) meta-analysis.

The diagnostic challenge is not limited to identifying malignancy. Contemporary lung-cancer classification requires separating benign/reactive processes from malignant disease and, among malignant tumors, accurately recognizing adenocarcinoma (ADC), squamous cell carcinoma (SCC), and small-cell lung cancer (SCLC).^7^ The 2021 WHO lung-tumor classification recommends classifying non-small-cell carcinoma in small biopsies and cytology as a more specific type, such as ADC or SCC, whenever possible.^8^ This task is clinically important but difficult: an IASLC cytology working group study found that conventional cytology subtyping of NSCLC had substantial rates of non-small-cell carcinoma—not otherwise specified, and that non-keratinizing SCC remained especially challenging.^9^

AI-based cytology models could potentially provide reproducible image- or slide-level support, reduce missed malignant cells, flag difficult cases for expert review, and improve consistency in labs with variable cytopathology resources.^10^ However, individual studies have used different specimen types, image units, validation designs, algorithms, reference standards, and reporting metrics.^5^ Therefore, the clinical value of AI in lung cancer cytology cannot be judged reliably from isolated accuracy reports alone.

Against this background, and given the relative underrepresentation of cytology-focused AI research compared with histopathology- and radiology-based applications, this systematic review and DTA meta-analysis comprehensively evaluated AI-based lung cancer classification in cytology specimens. The primary objective was to estimate pooled diagnostic performance for benign–malignant discrimination. Secondary objectives were to summarize performance for ADC/LUAD, SCC/SQCC, and SCLC one-versus-rest subtyping; evaluate likelihood ratios (LRs), diagnostic odds ratios (DOR), summary receiver operating characteristic (SROC) performance, post-test probability shifts, threshold effects, and small-study effects; and assess study-level risk of bias and applicability. The primary objective was to estimate pooled diagnostic performance for benign–malignant discrimination. Secondary objectives were to summarize performance for ADC/LUAD, SCC/SQCC, and SCLC one-versus-rest subtyping; evaluate LRs, DOR SROC performance, post-test probability shifts, threshold effects, and small-study effects; and assess study-level risk of bias and applicability.

## Materials and methods

### Protocol, registration, and reporting framework

This systematic review and DTA meta-analysis was registered in the Open Science Framework (OSF) Registries (https://doi.org/10.17605/OSF.IO/GDXPY). The review was reported in accordance with PRISMA-DTA, the PRISMA extension for systematic reviews and meta-analyses of DTA studies.^11^ The methods were organized around the DTA components required for transparent reporting: participants and specimens, index tests, target conditions, reference standards, study design, units of assessment, thresholds, and synthesis methods.

### Review the question and the intended clinical role of the index test

The review question was framed as follows. The population comprised human cytology specimens or cytology- or specimen-derived diagnostic data obtained in the setting of suspected or confirmed lung cancer. The index tests were AI, machine-, or deep-learning models applied to cytology images, whole-slide images, cells, patches, spectra, or specimen-level features. The target conditions were benign–malignant lung cytology diagnoses and lung cancer subtyping, including ADC/LUAD, SCC/SQCC/LUSC, and SCLC. Acceptable reference standards included histopathology, expert cytopathology consensus, clinical follow-up, or adjudicated clinical diagnosis. The intended clinical role of the index test was adjunctive cytopathology triage or diagnostic support, not autonomous replacement of pathologist diagnosis, ancillary testing, or clinicoradiological correlation.

### Information sources and search strategy

Seven bibliographic databases were searched on May 11, 2026: Embase, Europe PMC, OpenAlex, PubMed, Scopus, Semantic Scholar, and Web of Science. Search concepts combined lung cancer terms, cytology or cytopathology specimen terms, AI or machine-learning terms, and diagnostic classification or accuracy terms. Full database-specific search strings, date searched, limits or filters, and record yields are reported in Supplementary Table S1.

### Eligibility criteria

Studies were eligible if they evaluated an AI, machine-, or deep-learning approach for classifying human lung cancer cytology specimens or cytology-derived diagnostic data. Eligible diagnostic targets included benign–malignant discrimination and ADC/LUAD, SCC/SQCC/LUSC, and/or SCLC subtyping. Studies were excluded when they focused solely on radiological imaging, radiomics-only imaging, prognosis, treatment response, mutation prediction without cytology classification, lymph-node staging without cytology classification, sample adequacy only, cell-line-only material, preprocessing-only or captioning-only methods, mixed-cancer cytology without lung-specific outcomes, or lacked extractable diagnostic classification outcomes. The eligibility criteria by population, specimen, index test, target condition, reference standard, outcome, study type, and language/date domain are detailed in Supplementary Table S2.

### Study selection

Records were deduplicated using available identifiers and bibliographic fields, including DOI, PMID, and normalized title. Screening proceeded through sequential stages: duplicate removal; exclusion of non-primary or ineligible publication types; removal of studies unrelated to lung cancer; exclusion of wrong modality, wrong specimen type, or wrong endpoint; and final manual eligibility review for AI-based cytology or cytology-derived lung-cancer classification outcomes. Study-selection tracking, including PRISMA-DTA flow categories and links to the extracted data sources, is summarized in Supplementary Table S3. Study selection was performed independently by two reviewers. Titles and abstracts were screened against the predefined eligibility criteria, followed by independent full-text assessment of potentially eligible articles. Disagreements at either stage were resolved through discussion and consensus; when consensus could not be reached, an additional reviewer adjudicated the final decision.

### Data extraction and study characteristics

For each included study, extracted study-level characteristics included study identifier, country or region, design and setting, cytology specimen and preparation, population and sample size, target task, reference standard, and source reference. These study characteristics are summarized in Supplementary Table S4. Extracted AI model and dataset characteristics included model identifier, algorithm or architecture, input unit, annotation and ground-truth level, training/validation/test/external sample sizes, threshold rule, validation design, and code or data availability; these are summarized in Supplementary Table S5. Data were extracted independently by two reviewers using predefined structured extraction fields. Extracted information was cross-checked for consistency, and discrepancies were resolved through discussion and consensus, with adjudication by an additional reviewer when necessary. When multiple publications, datasets, model variants, or diagnostic targets originated from the same underlying study, these were examined to minimize inappropriate duplication and to preserve the correct unit of analysis.

### Risk of bias and applicability

Risk of bias and applicability were assessed using QUADAS-2 domains adapted for AI diagnostic-accuracy studies, including patient selection, index test, reference standard, flow and timing, and overall applicability. AI-specific signaling considered potential data leakage, separation of training and testing data, external validation, threshold derivation, and whether model evaluation approximated the intended clinical context. Domain-level and overall judgments are reported in Supplementary Table S6. Risk-of-bias and applicability assessments were performed independently by two reviewers. Disagreements in domain-level or overall judgments were resolved by discussion and consensus and, when necessary, by adjudication involving an additional reviewer.

### Diagnostic accuracy data extraction

For each eligible diagnostic-test-accuracy row, the extracted data included the diagnostic target, specimen or data unit, threshold or decision rule, reference standard, unit of analysis, inclusion decision for meta-analysis, and, when available, 2 × 2 table components (true positive (TP), false positive (FP), false negative (FN), and true negative (TN)). Benign–malignant DTA rows are reported in Supplementary Table S7. ADC/SCC/SCLC one-versus-rest subtype rows are reported in Supplementary Table S8. Multiclass ADC/SCC/SCLC confusion-matrix extraction layouts are reported in Supplementary Table S9. Reported diagnostic performance metrics that were not always directly usable as 2 × 2 DTA data, including accuracy, area under the curve (AUC), precision/positive-predictive value (PPV), recall/sensitivity, F1 score, LRs, and other metrics, are summarized in Supplementary Table S10. Threshold, index-test, and reference-standard details are summarized in Supplementary Table S11.

The original unit of analysis reported by each study was retained and explicitly coded as cell-, patch/image-field-, whole-slide-, spectral-, specimen-level, or another clearly defined analytical unit. No attempt was made to mathematically transform different analytical units into a common biological unit. A diagnostic row was eligible for quantitative synthesis only when a valid 2 × 2 contingency table could be extracted or reconstructed at the evaluation unit reported by the original study.

### Meta-analysis grouping and primary comparisons

Analyses were grouped according to prespecified clinical questions and target classes. The primary analysis evaluated AI-based benign–malignant diagnosis. Secondary analyses evaluated ADC/LUAD, SCC/SQCC/LUSC, and SCLC versus the rest. LCNEC versus SCLC was retained as an exploratory, non-primary subtype comparison when data were insufficient to pool. The grouping plan, inclusion rules, subgroup variables, sensitivity analyses, model/threshold rules, and planned outputs are summarized in Supplementary Table S12.

Quantitative pooling was primarily organized by diagnostic target rather than by assuming equivalence among different analytical units. Accordingly, cell-, patch-, whole-slide-, spectral-, and specimen-level evaluations could contribute to the same broad diagnostic-target synthesis when they provided valid 2 × 2 data; however, their original analytical units were preserved during extraction and interpretation. These units differ in biological meaning, within-specimen correlation, sampling structure, effective sample size, and relationship to the final clinical diagnosis and therefore should not be considered intrinsically interchangeable. Consequently, pooled estimates across heterogeneous analytical units were interpreted as broad random-effects summaries of AI diagnostic discrimination rather than as evidence of direct equivalence between analytical scales.

### Diagnostic accuracy measures and statistical synthesis

For each 2 × 2 row, sensitivity, specificity, PPV, negative-predictive value, accuracy, prevalence, positive LR (LR+), negative LR (LR-), DOR, Youden index, and exact binomial 95% confidence intervals for sensitivity and specificity were calculated. Pooled sensitivity and specificity were estimated using bivariate logit random-effects models. LR+, LR-, and DOR were pooled using random-effects meta-analysis on the log scale. Rows with zero cells used a Haldane–Anscombe correction for transformed LR, DOR, and logit-scale analyses while preserving raw cell counts for tabulation.

### Variability, threshold effects, and additional analyses

Between-study variability was summarized using Cochran's Q and, where applicable, I^2^, and SROC curves and AUCs were estimated using the Moses–Littenberg SROC models. Threshold effects were evaluated by Spearman correlation between logit sensitivity and logit false-positive rate. Small-study and publication-bias patterns were explored using Deeks funnel asymmetry regression. Fagan nomograms used pooled prevalence as a standardized pre-test probability for illustrative purposes. Because prevalence varied across studies and may not represent a typical real-world clinical population, the resulting post-test probabilities were interpreted as scenario-based demonstrations of the effect of pooled LRs rather than directly transportable estimates for individual patients or specific clinical settings. Planned heterogeneity, subgroup, and sensitivity-analysis coding included specimen type, cytology preparation, stain or imaging modality, algorithm family, validation design, threshold selection, unit of analysis, and risk of bias, as detailed in Supplementary Table S13.

### Certainty of evidence and data checking

The certainty of evidence for DTA outcomes was profiled using domains relevant to diagnostic accuracy evidence, including risk of bias, indirectness, variability/inconsistency, imprecision, and possible publication bias; the outcome-level certainty profile is provided in Supplementary Table S14. Data extraction discrepancies and adjudication status were recorded in a structured log, with final values retained after adjudication, as summarized in Supplementary Table S15. Operational definitions and formulae for TP, FP, FN, TN, sensitivity, specificity, LRs, DOR, zero-cell continuity correction, and independent dataset coding are provided in Supplementary Table S16.

## Results

### Study selection

**Fig. 1:**
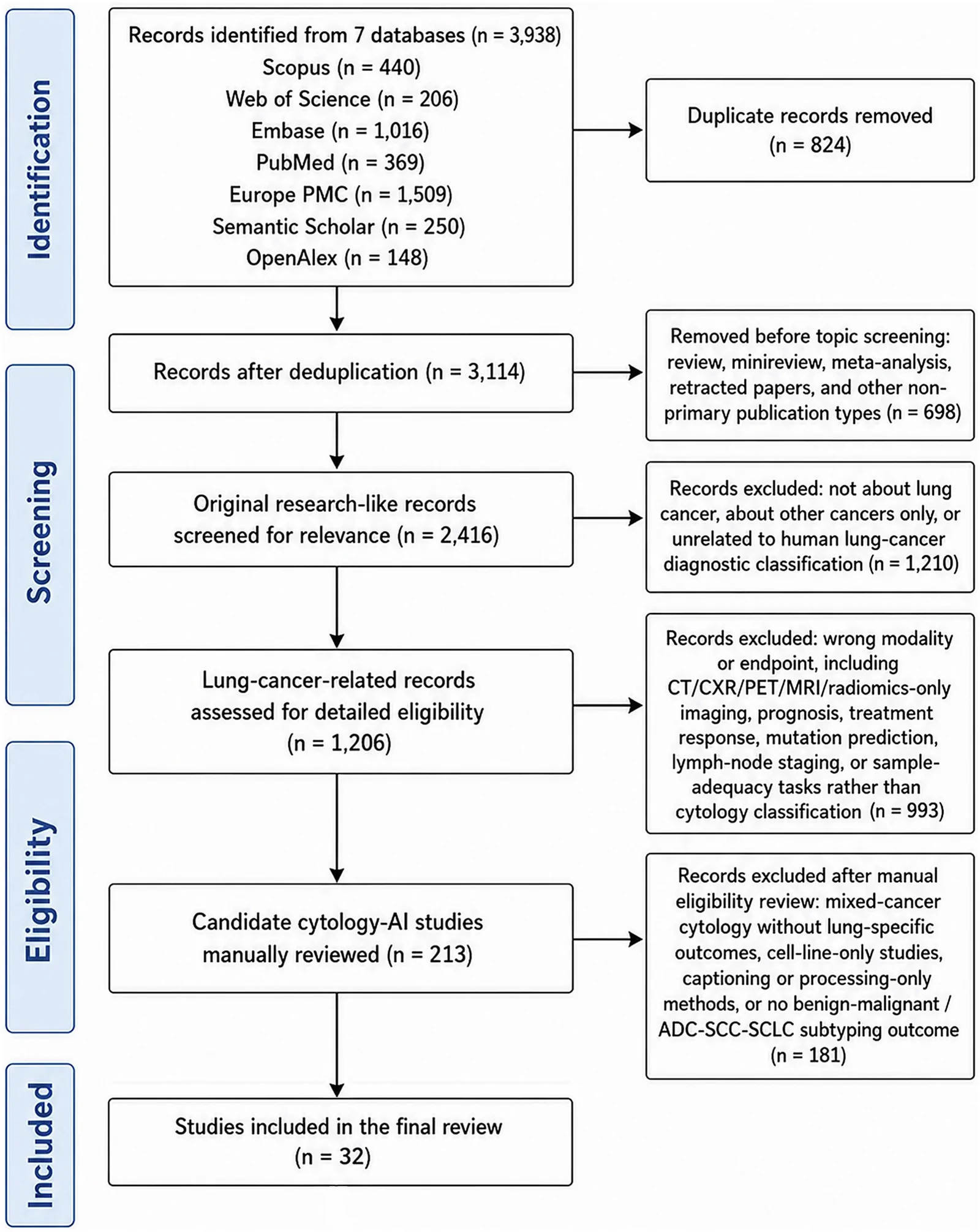
PRISMA flowchart of study screening and selection. Flow diagram showing identification, deduplication, screening, eligibility review, and final inclusion of studies evaluating AI for lung-cancer classification in cytology specimens.

The search identified 3938 records across seven databases. After removing 824 duplicates, 3114 records remained. Screening excluded 698 non-primary or ineligible publication types, leaving 2416 records that were originally research-like. A further 1210 records were excluded because they were not about lung cancer, focused only on other cancers, or were unrelated to human lung cancer diagnostic classification. Of 1206 lung-cancer-related records, 993 were excluded for incorrect modality, specimen type, or endpoint, leaving 213 candidate cytology- or specimen-based records. Manual eligibility review excluded 181 records, leaving 32 studies for the final review (Fig. 1).

### Benign–malignant diagnosis

**Fig. 2:**
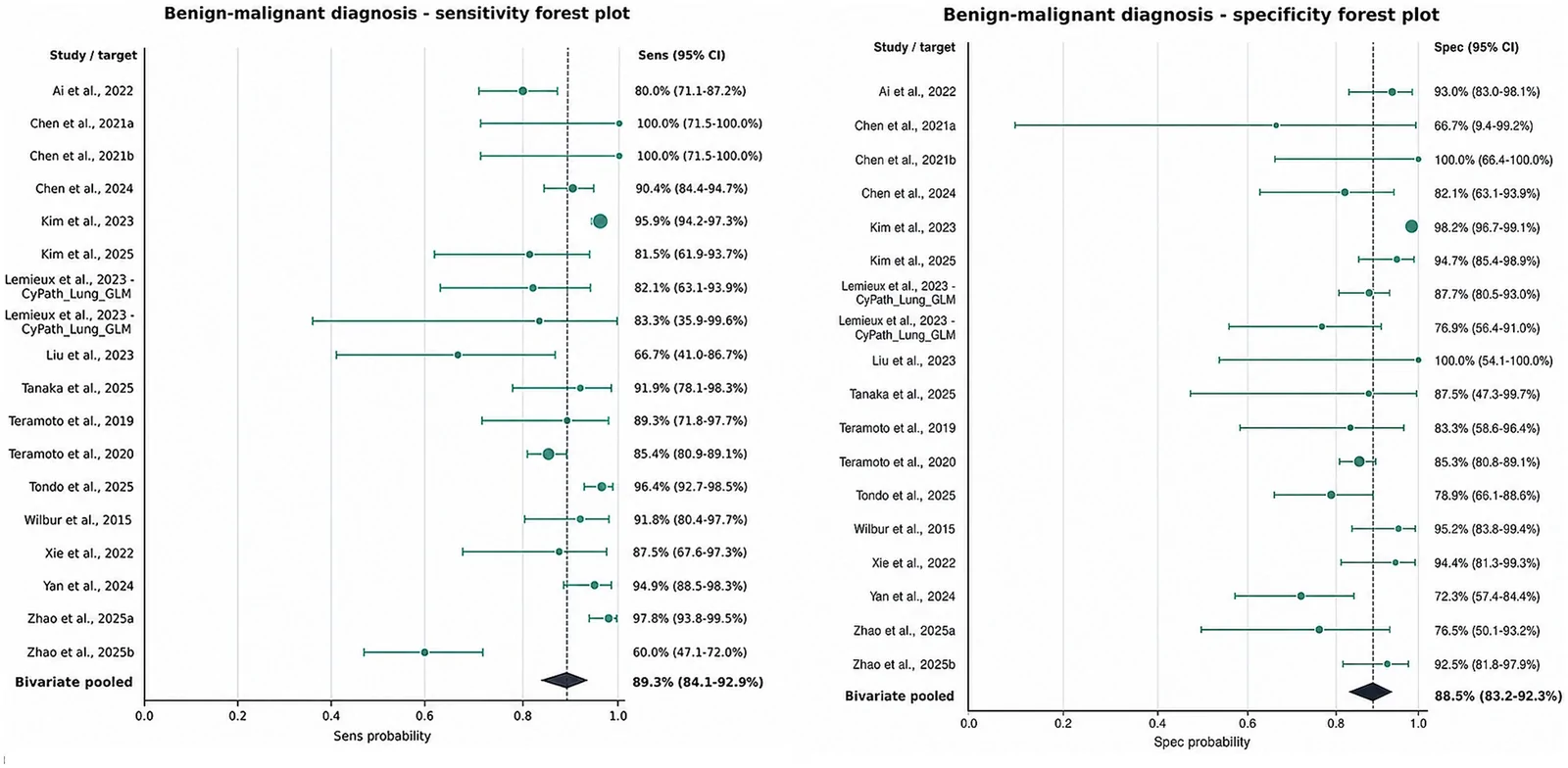
Benign–malignant diagnosis: sensitivity and specificity forest plots. Forest plots of study-level sensitivity and specificity for AI-based benign–malignant lung-cancer classification in cytology specimens. Point estimates are exact binomial estimates, and confidence intervals are Clopper–Pearson intervals. The bivariate pooled estimates were sensitivity 89.3% and specificity 88.5%, with substantial heterogeneity.

Eighteen benign–malignant DTA rows, including 3464 observations, were meta-analyzed. Individual study sensitivity ranged widely, but the bivariate pooled sensitivity was 89.3% (95% confidence interval (CI), 84.1–92.9%). Pooled specificity was 88.5% (95% CI, 83.2–92.3%). Heterogeneity was substantial, with I^2^ of 86.3% for sensitivity and 76.5% for specificity (Fig. 2).

**Fig. 3:**
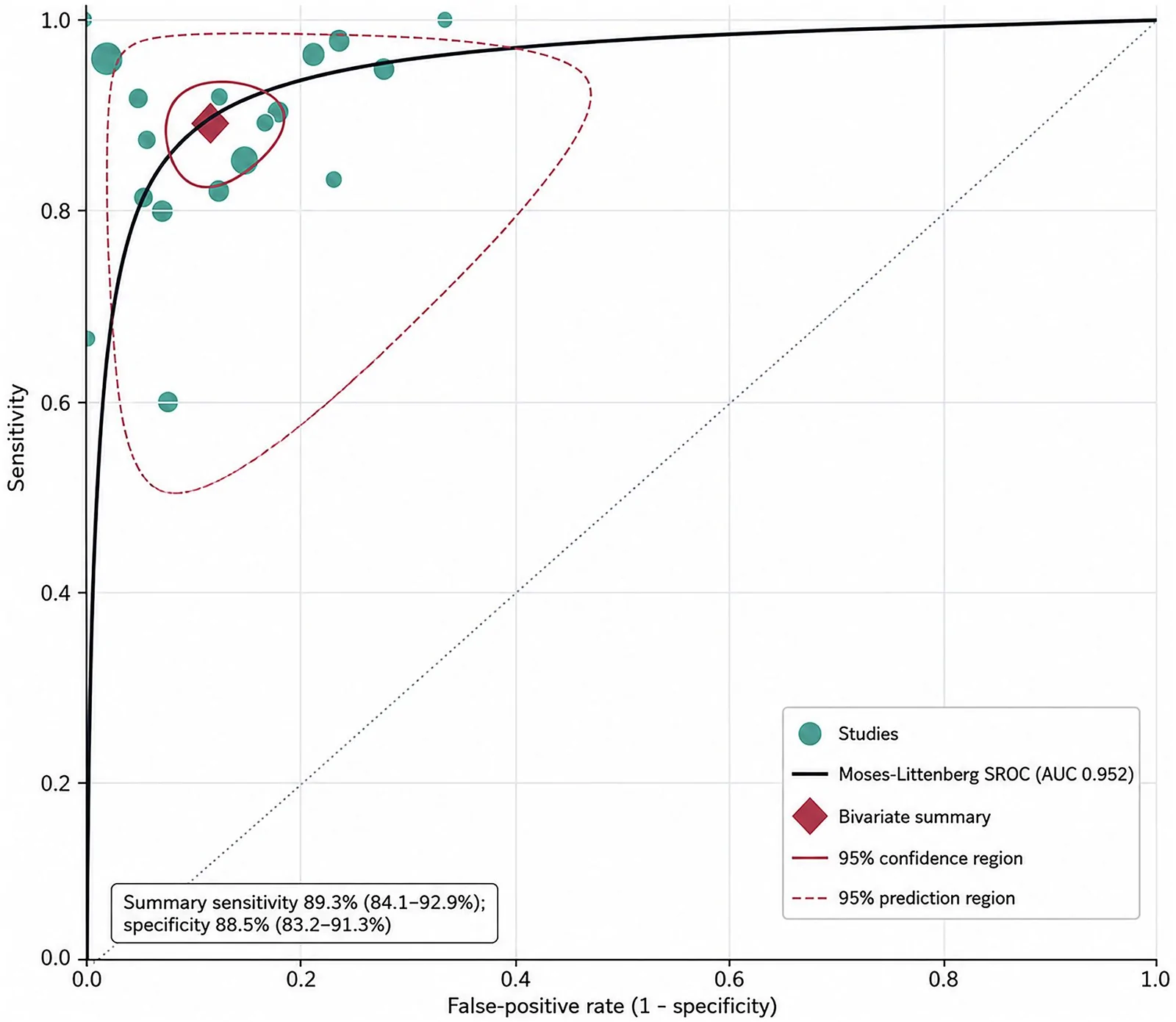
Benign–malignant diagnosis summary receiver operating characteristic (SROC) and bivariate summary plot. SROC plot for benign–malignant AI cytology classification. Circles represent individual studies, the diamond indicates the bivariate summary point, and ellipses show approximate 95% confidence and prediction regions. The SROC AUC was 0.952.

SROC analysis showed strong global discrimination for benign–malignant diagnosis. The Moses–Littenberg SROC AUC was 0.952, and the bivariate summary point corresponded to a sensitivity of 89.3% and a specificity of 88.5%. The prediction region was wider than the confidence region, indicating between-study variability and the likelihood that future study performance may vary across settings, specimen types, and validation designs (Fig. 3).

**Fig. 4:**
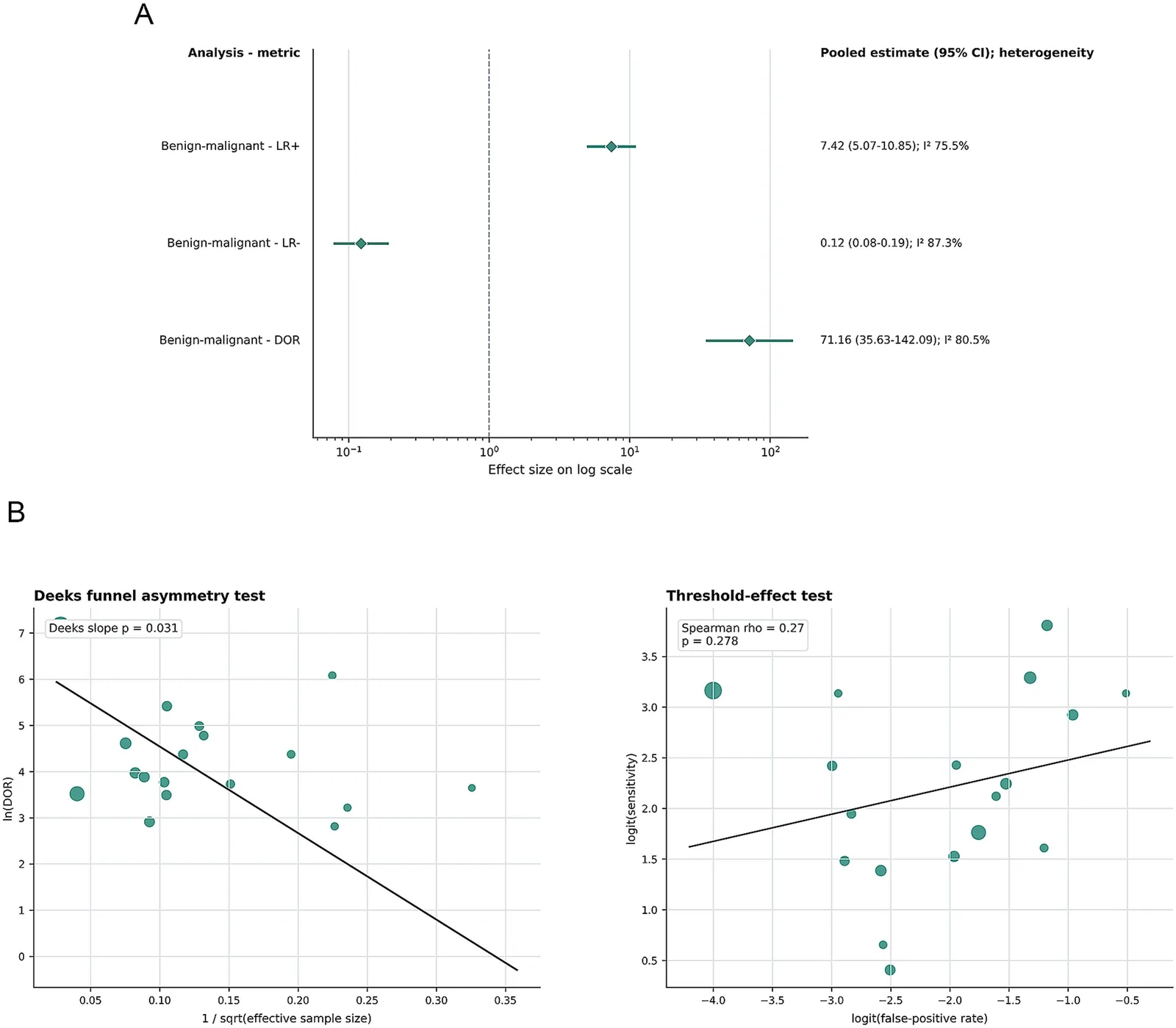
Benign–malignant pooled likelihood ratios (LRs), diagnostic odds ratio (DOR), and diagnostic-bias/threshold diagnostics. Panel A shows pooled LR+, LR−, and DOR on the log scale for benign–malignant diagnosis. Panel B shows Deeks funnel asymmetry testing and threshold-effect testing using Spearman correlation between logit sensitivity and logit false-positive rate. Deeks testing suggested small-study effects or publication bias, whereas the threshold-effect test was not statistically significant.

LR synthesis showed an LR+ of 7.42 (95% CI, 5.07–10.85), LR− of 0.12 (95% CI, 0.08–0.19), and DOR of 71.16–71.2 (95% CI, approximately 35.6–142.1). These values indicate a strong overall association between AI test results and benign–malignant status, although the LR+ remained below the conventional threshold of 10, which is often used as strong rule-in evidence. Deeks funnel asymmetry testing was positive for the benign–malignant analysis (*p* = 0.031), whereas threshold-effect testing did not show a statistically significant threshold pattern (Spearman rho = 0.27, *p* = 0.278) (Fig. 4).

**Fig. 5:**
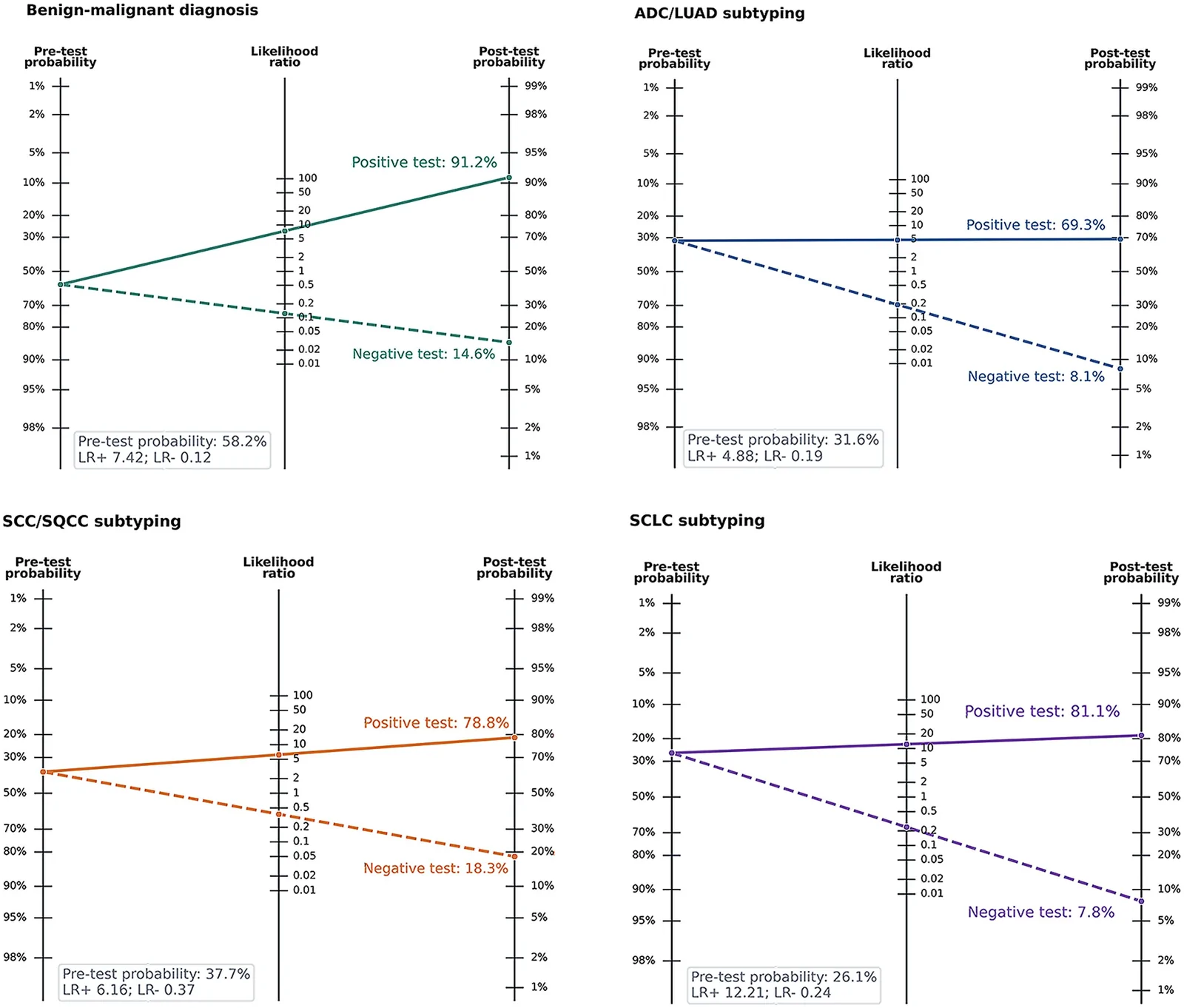
Illustrative Fagan nomograms based on pooled prevalence and pooled likelihood ratios. Fagan nomograms demonstrating changes from pre-test to post-test probability following positive and negative AI results for benign–malignant diagnosis and ADC/LUAD, SCC/SQCC, and SCLC subtyping. Pooled prevalence was used as a standardized pre-test probability for illustrative purposes. Because of substantial heterogeneity in study populations, case selection, clinical settings, specimen types, imaging approaches, and units of analysis, these prevalence values may not represent typical real-world clinical populations. Accordingly, the reported post-test probabilities should be interpreted as scenario-based illustrations of the effect of pooled likelihood ratios rather than as directly transferable estimates for individual patients or specific clinical settings.

For illustrative Fagan analysis, the pooled benign–malignant prevalence of 58.2% was used as a standardized pre-test probability. Under this specified scenario, a positive AI result increased the post-test probability of malignancy to 91.2%, whereas a negative result reduced it to 14.6% (Fig. 5). Because the contributing studies were heterogeneous in population characteristics, case selection, specimen type, clinical setting, and unit of analysis, the pooled prevalence should not be regarded as representative of a single typical real-world clinical population. Accordingly, these post-test probabilities are illustrative and should not be applied directly to individual patients or clinical settings without first applying an appropriate setting-specific pre-test probability. The contributing studies varied in their analytical scale, including cell-, image/patch-, whole-slide-, spectral-, and specimen-level evaluations. Therefore, the pooled benign–malignant estimates represent an overall summary across heterogeneous AI-cytology implementations and should not be interpreted as demonstrating direct equivalence among these analytical units.

### ADC/LUAD, SCC/SQCC, and SCLC subtyping

**Fig. 6:**
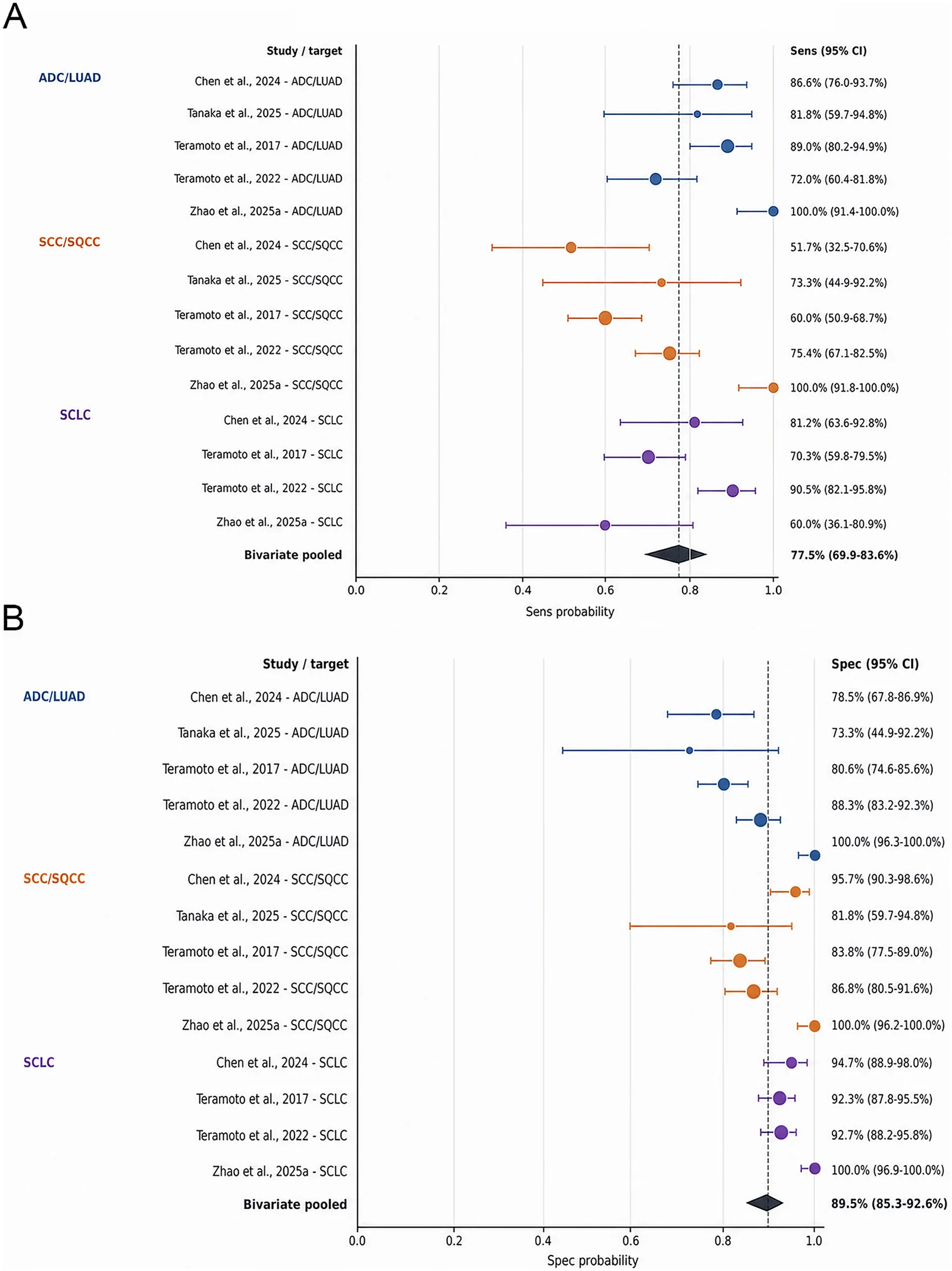
ADC/SCC/SCLC one-versus-rest subtyping: sensitivity and specificity forest plots. Forest plots of study-level sensitivity and specificity for one-versus-rest AI classification of ADC/LUAD, SCC/SQCC, and SCLC in cytology specimens. The bivariate pooled overall subtyping estimates were sensitivity 77.5% and specificity 89.5%. Estimates should be interpreted cautiously because several class rows were derived from the same multiclass studies.

Fourteen one-versus-rest rows involving 2687 observations were included in the overall ADC/SCC/SCLC subtyping meta-analysis. The pooled sensitivity was 77.5% (95% CI, 69.9–83.6%), and the pooled specificity was 89.5% (95% CI, 85.3–92.6%). Heterogeneity remained substantial, with I^2^ of 78.2% for sensitivity and 77.6% for specificity (Fig. 6). Because several one-versus-rest class rows were derived from the same multiclass studies, the pooled overall subtype estimates should be interpreted with caution, and class-specific results are more clinically informative. Additional methodological heterogeneity arose from differences in the unit of analysis and imaging approach. Because several estimates were derived at different analytical scales and some one-versus-rest rows originated from the same multiclass studies, the overall subtype summary should be viewed primarily as an aggregate description of the available evidence rather than a single universally transferable measure of clinical diagnostic performance.

**Fig. 7:**
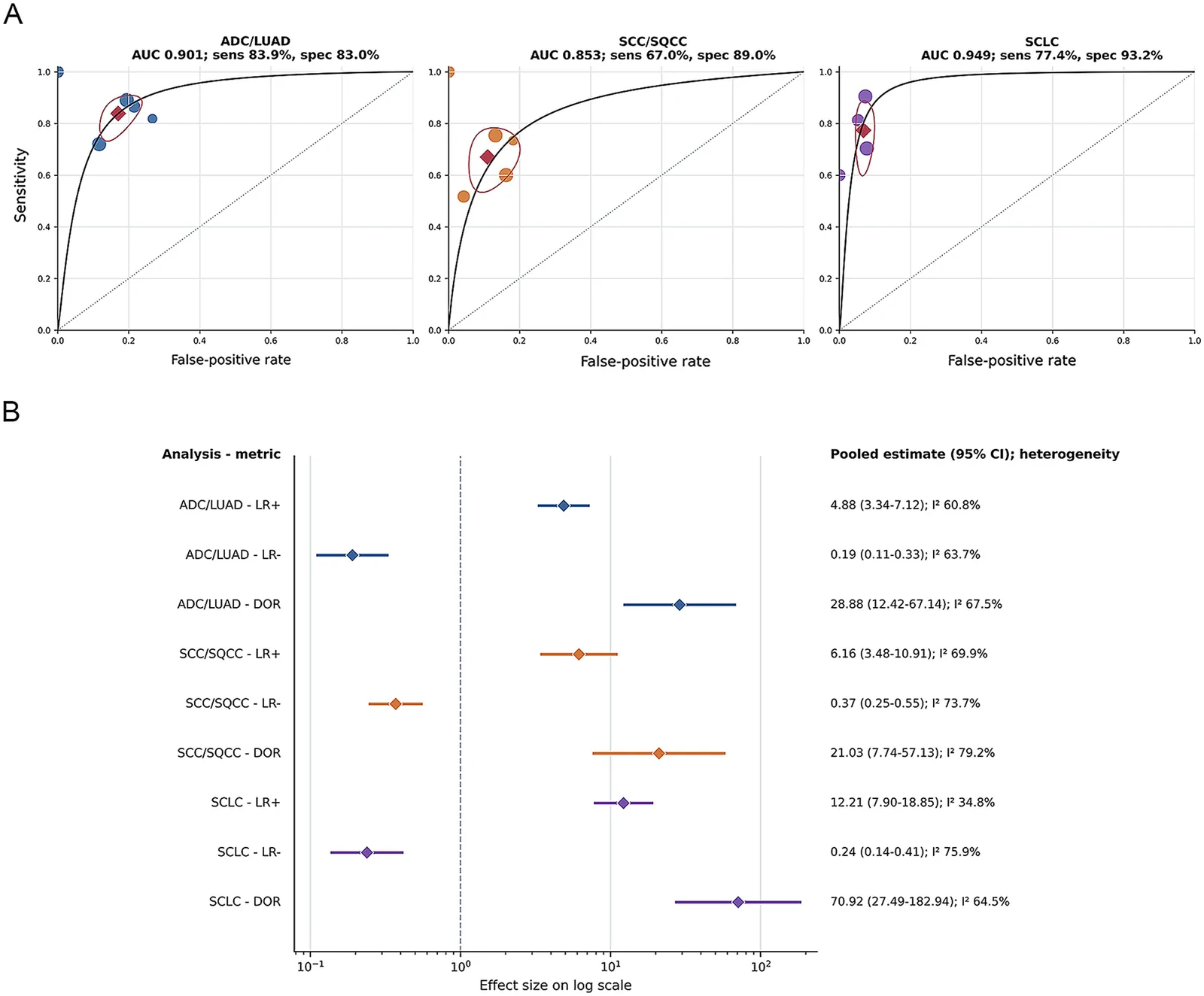
Class-specific SROC plots and pooled likelihood ratios/diagnostic odds ratios for subtyping. Panel A shows class-specific SROC plots for ADC/LUAD, SCC/SQCC, and SCLC. Panel B shows pooled LR+, LR−, and DOR for each subtype class. SCLC showed the strongest rule-in profile, with a specificity of 93.2%, an LR+ of 12.21, a DOR of 70.92, and an AUC of 0.949.

Class-specific SROC and LR analyses revealed distinct diagnostic profiles across tumor classes. ADC/LUAD subtyping had a sensitivity of 83.9% (95% CI, 75.6–89.8%), a specificity of 83.0% (95% CI, 77.7–87.2%), a LR+ of 4.88, a LR− of 0.19, a DOR of 28.9, and an AUC of 0.901. SCC/SQCC subtyping had a sensitivity of 67.0% (95% CI, 56.2–76.3%), a specificity of 89.0% (95% CI, 82.1–93.5%), a LR+ of 6.16, a LR− of 0.37, a DOR of 21.0, and an AUC of 0.853. SCLC subtyping had a sensitivity of 77.4% (95% CI, 63.7–87.0%), a specificity of 93.2% (95% CI, 90.8–95.1%), a LR+ of 12.21, a LR− of 0.24, a DOR of 70.9, and an AUC of 0.949 (Fig. 7).

The SCLC results showed the strongest rule-in profile among the subtype groups, with specificity exceeding 93% and LR+ exceeding 10. In contrast, SCC/SQCC showed the lowest pooled sensitivity, indicating that AI models may miss a larger proportion of SCC/SQCC cases than ADC/LUAD or SCLC cases. Fagan nomograms showed that, at pooled class prevalence, positive AI tests increased the post-test probability to 69.3% for ADC/LUAD, 78.8% for SCC/SQCC, and 81.1% for SCLC; negative tests reduced the post-test probability to 8.1%, 18.3%, and 7.8%, respectively (Fig. 5). As with the benign–malignant analysis, these values represent illustrative probability shifts under the pooled-prevalence scenarios used in the nomograms and should not be interpreted as universally applicable clinical probabilities.

**Fig. 8:**
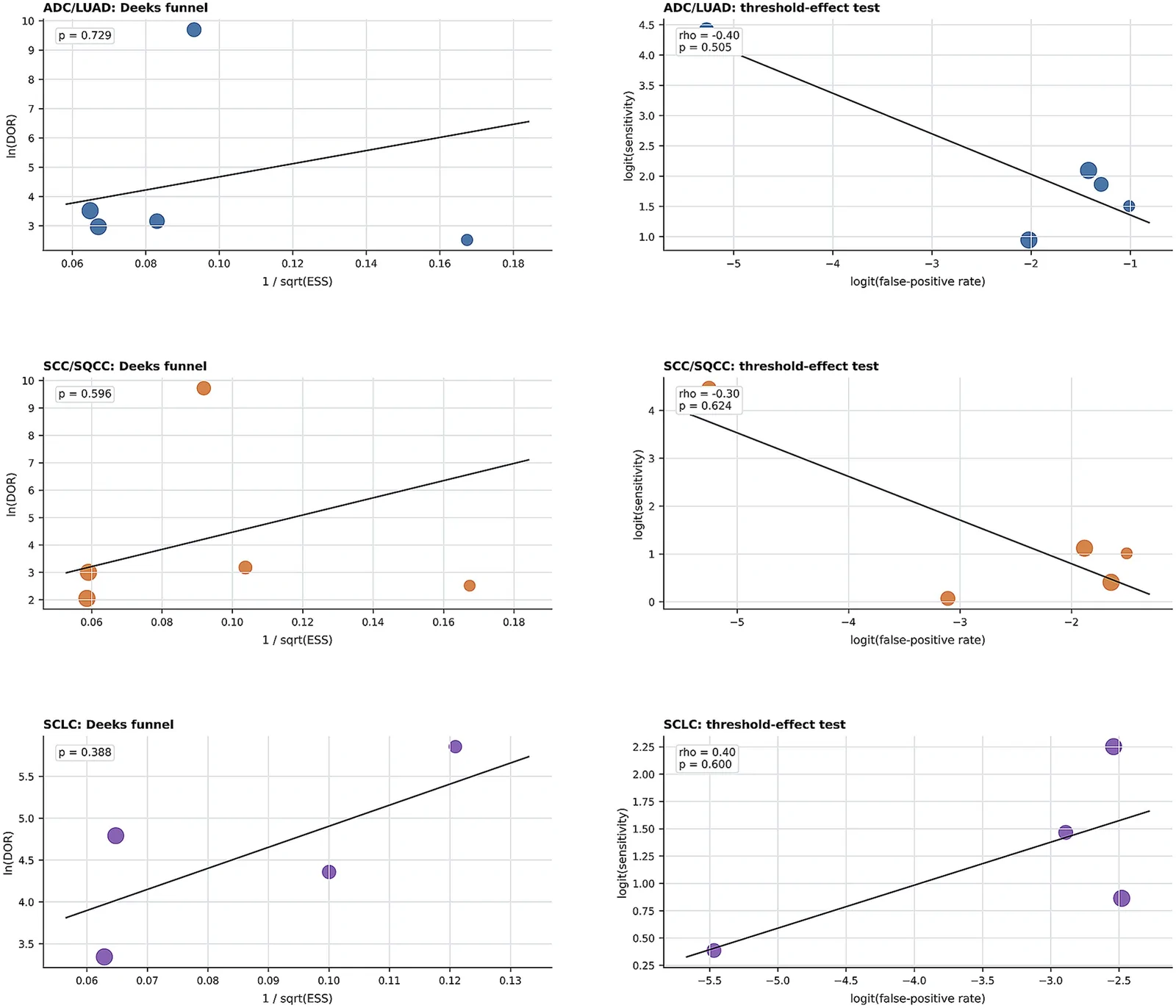
Class-specific publication-bias and threshold-effect diagnostics for subtyping. Deeks funnel asymmetry plots and threshold-effect plots for ADC/LUAD, SCC/SQCC, and SCLC one-versus-rest subtyping. No class-specific Deeks or threshold-effect test was statistically significant, although interpretation is limited by small class-specific sample sizes and non-independence among some rows.

Class-specific Deeks tests did not show statistically significant funnel asymmetry for ADC/LUAD (*p* = 0.729), SCC/SQCC (*p* = 0.596), or SCLC (*p* = 0.388). Threshold-effect testing was also not statistically significant for ADC/LUAD (rho = −0.40, *p* = 0.505), SCC/SQCC (rho = −0.30, *p* = 0.624), or SCLC (rho = 0.40, *p* = 0.600). These analyses were exploratory because class-specific sample sizes were small and several rows within source studies were correlated (Fig. 8).

### Risk of bias and applicability

**Fig. 9:**
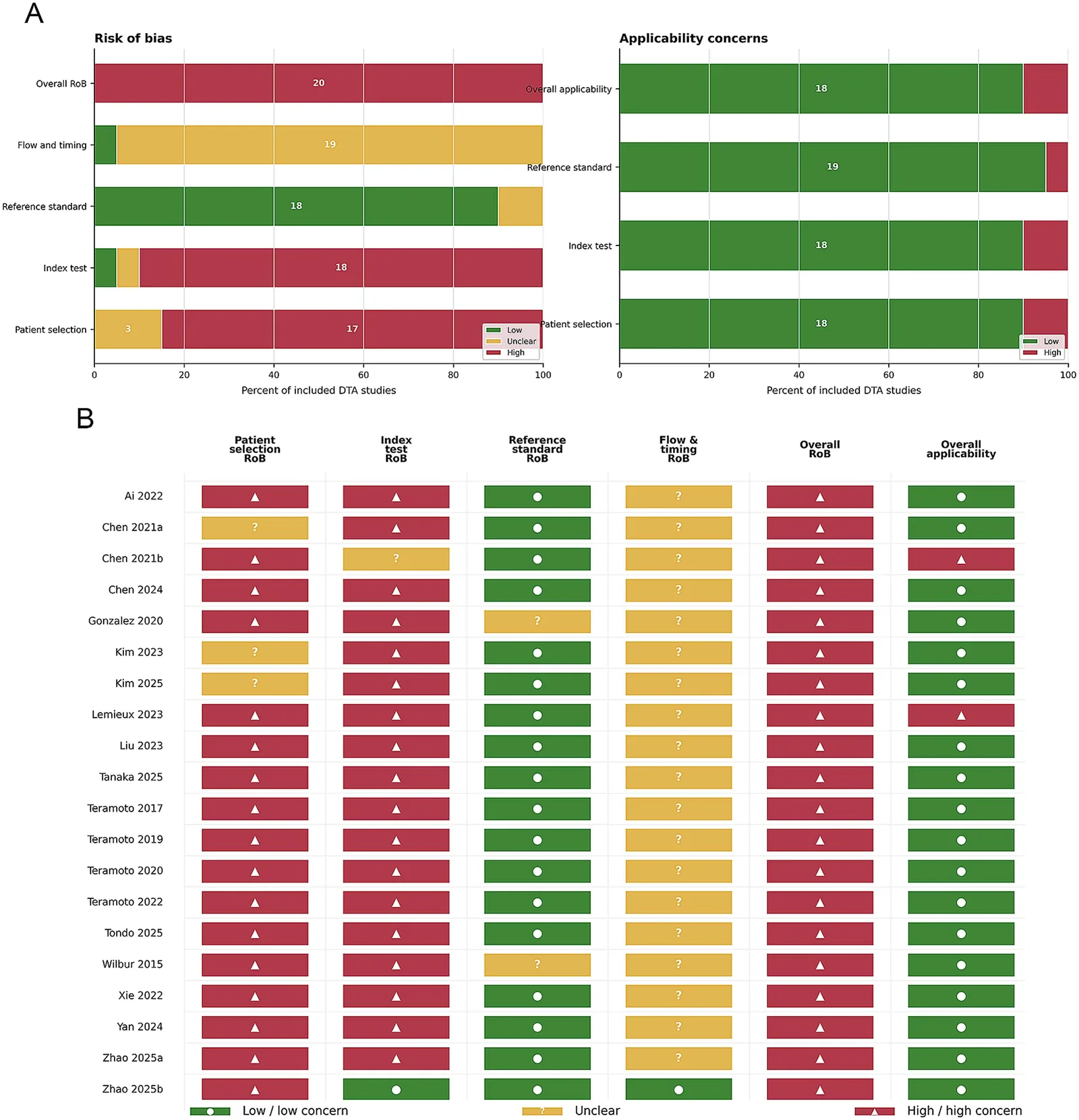
QUADAS-2/AI risk-of-bias and applicability assessment. Panel A summarizes the risk of bias and applicability concerns across the 20 unique DTA-contributing studies. Panel B presents a study-level traffic-light plot. Overall risk of bias was high across all DTA-contributing studies, whereas overall applicability concerns were low in 18 studies and high in 2.

Among the 20 unique DTA-contributing studies, the overall risk of bias was rated high in all. Patient-selection risk of bias was high in 17 studies and unclear in 3. Index-test risk of bias was high in 18, unclear in 1, and low in 1. Reference-standard risk of bias was low in 18 and unclear in 2. Flow and timing were unclear in 19 and low risk in 1. Overall applicability concern was low in 18 studies and high in 2 (Fig. 9).

## Discussion

This systematic review and DTA meta-analysis found that AI-based cytology classification has high pooled diagnostic performance for benign–malignant discrimination in lung cancer and promising, though more heterogeneous, performance for ADC/LUAD, SCC/SQCC, and SCLC subtyping. The benign–malignant pooled AUC of 0.952, sensitivity of 89.3%, specificity of 88.5%, LR of 0.12, and DOR of approximately 71 suggest clinically meaningful discrimination. The Fagan analysis further illustrates that AI outputs could substantially alter diagnostic probability under the specified pre-test probability scenarios. However, because pooled prevalence was derived from heterogeneous studies with differing clinical settings, patient-selection mechanisms, specimen types, disease spectra, and analytical units, it should not be interpreted as a universally representative clinical prevalence. The calculated post-test probabilities therefore demonstrate the diagnostic implications of the pooled LRs under an illustrative scenario rather than providing directly transferable estimates for individual patients or labs. In clinical application, post-test probabilities should be recalculated using the pre-test probability appropriate to the specific population and diagnostic setting.

The benign–malignant results are broadly consistent with prior meta-analytic evidence on AI-assisted lung-cancer diagnosis in radiological imaging. Liu et al. reported a pooled sensitivity of 0.87, a specificity of 0.87, a LR+ of 6.5, a LR− of 0.15, a DOR of 43, and an SROC AUC of 0.93 for AI-assisted lung-cancer diagnosis, largely in CT-based studies.^12^ The present cytology-focused estimates are similar or slightly stronger for sensitivity, LR−, DOR, and AUC. This comparison is encouraging, but the clinical contexts differ: CT-based AI typically addresses lesion detection or imaging diagnosis, whereas cytology-based AI evaluates cellular morphology in specimens already obtained for pathological diagnosis. Therefore, cytology AI may be best viewed as a pathology-assistance tool rather than a replacement for screening.

The findings also align with individual AI studies in cytology. Teramoto et al. used progressive growing generative adversarial networks to augment lung cytology image classification and reported an overall accuracy of 85.3%, approximately 4.3% higher than a prior approach without GAN-based pretraining.^13^ Xie et al. evaluated weakly supervised deep learning on whole-slide images of pleural effusion cytology and reported accuracies of 91.67%, sensitivity of 87.50%, specificity of 94.44%, and an AUC of 0.9526, which closely parallels the present pooled benign–malignant AUC.^14^ These consistencies support the biological and technical plausibility of AI-assisted cytology in recognizing malignant cells.

Subtyping results were more mixed. SCLC showed the strongest rule-in performance, with a specificity of 93.2%, a LR+ of 12.21, a DOR of 70.9, and an AUC of 0.949. This profile suggests that a positive AI SCLC output may be useful for prioritizing urgent expert confirmation, neuroendocrine-marker review, and appropriate clinical triage. ADC/LUAD classification was reasonably balanced, but SCC/SQCC sensitivity was lower at 67.0%. This is consistent with known diagnostic difficulty in conventional cytology. The IASLC cytology working group reported that concordant cytology subtyping was achieved in only 53.7% of responses and that suboptimal recognition of LUSC without keratinization remained a major hurdle.^9^ Thus, the lower SCC/SQCC sensitivity observed here likely reflects not only AI limitations but also the intrinsic morphological overlap and limited features available in cytology specimens.

Compared with histopathology-based AI, cytology AI is less mature. Deep-learning models applied to biopsy or whole-slide histology have achieved very high performance in some settings. Kanavati et al. reported an AUC of 0.99 for classifying indeterminate whole-slide images from transbronchial lung biopsies into ADC, SCC, SCLC, and non-neoplastic categories, using additional external test sets.^15^ Yang et al. developed a six-type histopathology classifier for lung ADC, lung SCC, SCLC, pulmonary tuberculosis, organizing pneumonia, and normal lung, achieving multicohort AUCs of 0.970, 0.918, 0.963, and 0.978.^16^ These histology studies benefit from architectural information, broader tissue context, and often more mature digital pathology pipelines. Cytology, by contrast, includes dispersed cells, variable preparation types, overlapping inflammatory and reactive changes, and fewer standardized public datasets.

An additional source of heterogeneity was the analytical scale at which AI performance was evaluated. Individual-cell and patch-level models classify local image content and may contain multiple correlated observations from the same patient or specimen, whereas whole-slide and specimen-level systems more closely approximate a case-level clinical decision. Spectral approaches introduce an additional distinction, as predictions may be based on high-dimensional optical or molecular signatures rather than on conventional cytomorphology alone. Consequently, these approaches are not directly interchangeable, and numerically similar estimates of sensitivity or specificity may have different clinical meanings depending on the evaluation unit. The random-effects synthesis therefore provides a broad summary of diagnostic discrimination across the available AI-cytology literature rather than establishing equivalence between cell-, patch-, whole-slide-, spectral-, and specimen-level systems. Future evidence syntheses would benefit from sufficiently large evidence bases permitting separate meta-analyses by unit of analysis, specimen preparation, imaging modality, and validation design.

The primary concern in this review is methodological quality. Overall risk of bias was high across all 20 DTA-contributing studies, primarily due to patient selection, index-test design, validation limitations, and uncertain flow/timing. High heterogeneity further suggests that pooled estimates should not be interpreted as fixed performance expected across all labs. Many AI studies remain retrospective, internally validated, and algorithm-centered rather than workflow-centered. This mirrors broader concerns in lung-cancer AI translation, where robust multicenter validation, fairness, data sharing, regulatory readiness, and clinical integration remain central barriers.^17^

Clinically, the most realistic near-term role of AI in lung-cancer cytology is as an adjunct to, rather than an autonomous, diagnostic system. AI may help pre-screen slides, identify high-risk fields, triage cases for rapid pathologist review, support less-experienced observers, reduce FNs in high-volume labs, and provide a second-reader function for benign–malignant discrimination. For subtyping, AI outputs should be integrated with cytomorphology, immunocytochemistry, cell block findings, molecular testing needs, and clinical-radiological context.

## Future directions

Future studies should move from retrospective algorithm evaluation to prospective, multicenter clinical validation. Datasets should include diverse cytology preparations, stains, scanners, institutions, and patient populations, with explicit separation of training, validation, and external test cohorts. Subtyping models should be developed as calibrated multiclass tools rather than relying solely on one-versus-rest analyses, and should report class-level confusion matrices, confidence scores, rejected/uncertain cases, and performance in low-cellularity or diagnostically difficult specimens.

Human–AI interaction studies are needed to determine whether AI improves cytopathologist performance, turnaround time, interobserver agreement, and triage efficiency. Future work should also evaluate integration with immunocytochemistry, cell-block assessment, molecular testing workflows, and multimodal clinical data. Federated learning and privacy-preserving model development may help overcome barriers to multi-institutional data sharing. Reporting should follow current AI and diagnostic-accuracy guidance, including transparent model development, calibration, external validation, failure analysis, and equity assessment. TRIPOD+AI and related reporting initiatives provide useful guidance on transparent reporting of machine-learning models.^5^

## Study limitations

This review has several limitations. First, only 20 of the 32 included studies contributed analyzable DTA data, and the meta-analysis relied on extracted 2 × 2 rows rather than uniform patient-level datasets. Second, substantial heterogeneity existed across specimen type, preparation technique, staining or imaging modality, scanner platform, AI architecture, validation design, reference standard, and unit of analysis. In particular, cell-, patch-, whole-slide-, spectral-, and specimen-level models differ in sampling structure, within-specimen correlation, effective sample size, biological interpretation, and proximity to the final clinical diagnostic decision. Although the original unit of analysis was preserved during extraction, some heterogeneous units contributed to the same broad diagnostic-target synthesis when valid 2 × 2 data were available. Therefore, the resulting pooled estimates should be regarded as broad random-effects summaries rather than evidence that the different analytical units are directly comparable or clinically interchangeable. Third, several subtype rows were non-independent because multiple one-versus-rest class results came from the same multiclass source studies. Fourth, class-specific subgroup sizes were small, especially for SCLC, making threshold and publication-bias analyses exploratory. Fifth, the LCNEC/SCLC result was based on one row and could not be pooled. Sixth, the benign–malignant Deeks test suggested possible small-study effects or publication bias. In addition, the Fagan nomograms used pooled prevalence as a standardized illustrative pre-test probability. Given the marked heterogeneity in populations, case selection, specimens, and diagnostic settings, this pooled prevalence may not represent routine clinical practice; therefore, the corresponding post-test probabilities should be considered illustrative rather than directly generalizable. Finally, the overall risk of bias was high in all DTA-contributing studies, limiting confidence in direct clinical generalizability.

## Conclusion

AI-based lung-cancer classification in cytology specimens shows high pooled accuracy for benign–malignant diagnosis and promising performance for ADC/LUAD, SCC/SQCC, and SCLC subtyping. SCLC had the strongest rule-in profile, whereas SCC/SQCC remained the most challenging subtype. Despite encouraging diagnostic performance, substantial heterogeneity, high risk of bias, small class-specific evidence bases, and limited external validation, these factors prevent immediate routine clinical adoption. AI should currently be considered an adjunctive cytopathology tool requiring prospective validation, standardized reporting, and workflow-based evaluation before clinical deployment.

## CRediT authorship contribution statement

**Ayan Myssayev:** Data curation; Writing – original draft; **Amin Tamadon:** Conceptualization; Methodology; Formal analysis; Writing – original draft; **Nadiar M. Mussin:** Supervision; Methodology; **Reza Shirazi:** Data curation; All authors: Investigation; Writing – review & editing; Final approval of the manuscript; **Afshin Zare:** Conceptualization; Formal analysis.

All authors have read and approved the final manuscript.

## Informed consent

Not applicable.

## Compliance with ethical standards

Not applicable.

## Funding

This research received no external funding.

## Declaration of competing interest

The authors declare that they have no known competing financial interests or personal relationships that could have appeared to influence the work reported in this article.

## Data Availability

All data generated or analyzed during this study are included in this published article and its supplementary information files. The extracted diagnostic test accuracy datasets, study-level characteristics, risk-of-bias assessments, and meta-analytic outputs are available from the corresponding author upon reasonable request. The review protocol was prospectively registered in the Open Science Framework (OSF) Registries (https://doi.org/10.17605/OSF.IO/GDXPY).

## References

[1] Li Y., Yan B., He S. (2023). Advances and challenges in the treatment of lung cancer. Biomed Pharmacother.

[2] Gandhi Z., Gurram P., Amgai B. (2023). Artificial intelligence and lung cancer: impact on improving patient outcomes. Cancers.

[3] Morikawa K., Kida H., Handa H. (2022). A prospective validation study of lung cancer gene panel testing using cytological specimens. Cancers.

[4] Kim H., Chung J.-H. (2022). Biomarker testing of cytology specimens in personalized medicine for lung cancer patients. J Pathol Transl Med.

[5] Davri A., Birbas E., Kanavos T. (2023). Deep learning for lung cancer diagnosis, prognosis and prediction using histological and cytological images: a systematic review. Cancers.

[6] Hays P. (2024). Artificial intelligence in cytopathological applications for cancer: a review of accuracy and analytic validity. Eur J Med Res.

[7] Nabeel S.M., Bazai S.U., Alasbali N. (2024). Optimizing lung cancer classification through hyperparameter tuning. Digit Health.

[8] Nicholson A.G., Tsao M.S., Beasley M.B. (2022). The 2021 WHO classification of lung tumors: impact of advances since 2015. J Thorac Oncol.

[9] Jain D., Nambirajan A., Chen G. (2022). NSCLC subtyping in conventional cytology: results of the international association for the study of lung cancer cytology working group survey to determine specific cytomorphologic criteria for adenocarcinoma and squamous cell carcinoma. J Thorac Oncol.

[10] Yang Y., Guan S., Ou Z., Li W., Yan L., Situ B. (2023). Advances in AI-based cancer cytopathology. Interdiscip Med.

[11] McInnes M.D., Moher D., Thombs B.D. (2018). Preferred reporting items for a systematic review and meta-analysis of diagnostic test accuracy studies: the PRISMA-DTA statement. JAMA.

[12] Liu M., Wu J., Wang N. (2023). The value of artificial intelligence in the diagnosis of lung cancer: a systematic review and meta-analysis. PLoS One.

[13] Teramoto A., Tsukamoto T., Yamada A. (2020). Deep learning approach to classification of lung cytological images: two-step training using actual and synthesized images by progressive growing of generative adversarial networks. PLoS One.

[14] Xie X., Fu C.-C., Lv L. (2022). Deep convolutional neural network-based classification of cancer cells on cytological pleural effusion images. Mod Pathol.

[15] Kanavati F., Toyokawa G., Momosaki S. (2021). A deep learning model for the classification of indeterminate lung carcinoma in biopsy whole slide images. Sci Rep.

[16] Yang H., Chen L., Cheng Z. (2021). Deep learning-based six-type classifier for lung cancer and mimics from histopathological whole slide images: a retrospective study. BMC Med.

[17] Zhu E., Muneer A., Zhang J. (2025). Progress and challenges of artificial intelligence in lung cancer clinical translation. npj Precis Oncol.

