# Supplementary material for: Artificial intelligence for lung cancer classification in cytology specimens: A systematic review and diagnostic test accuracy meta-analysis of benign–malignant diagnosis and ADC/SCC/SCLC subtyping
Source: J Pathol Inform. 2026 Aug 3;22:100704. doi: 10.1016/j.jpi.2026.100704 (PMC13546853; doi:10.1016/j.jpi.2026.100704)
Supplement: Supplementary file 1 [file mmc1.docx]

# Artificial Intelligence for Lung Cancer Classification in Cytology Specimens: A Systematic Review and Diagnostic Test Accuracy Meta-analysis of Benign–Malignant Diagnosis and ADC/SCC/SCLC Subtyping

# Supplementary Tables

## Supplementary Table S1. Search strategy and database yield.

| **Database** | **Date searched** | **Search string** | **Limits/filters** | **Records retrieved** |
| --- | --- | --- | --- | --- |
| Embase | 11. May. 2026 | ('lung cancer'/exp OR 'lung cancer' OR 'lung carcinoma':ti,ab,kw OR 'pulmonary cancer':ti,ab,kw OR 'pulmonary carcinoma':ti,ab,kw OR 'non small cell lung cancer':ti,ab,kw OR nsclc:ti,ab,kw OR 'small cell lung cancer':ti,ab,kw OR sclc:ti,ab,kw OR adenocarcinoma:ti,ab,kw OR adc:ti,ab,kw OR 'squamous cell carcinoma':ti,ab,kw OR scc:ti,ab,kw) AND ('cytology'/exp OR 'cytology' OR cytology:ti,ab,kw OR cytologic*:ti,ab,kw OR cytopathology:ti,ab,kw OR 'fine needle aspiration'/exp OR 'fine needle aspiration' OR 'fna':ti,ab,kw OR 'ebus':ti,ab,kw OR 'tbna':ti,ab,kw OR 'ebus-tbna':ti,ab,kw OR sputum:ti,ab,kw OR 'pleural fluid':ti,ab,kw OR effusion:ti,ab,kw) AND ('artificial intelligence'/exp OR 'artificial intelligence' OR 'artificial intelligence':ti,ab,kw OR 'machine learning'/exp OR 'machine learning' OR 'deep learning'/exp OR 'deep learning' OR 'machine learning':ti,ab,kw OR 'deep learning':ti,ab,kw OR cnn:ti,ab,kw OR 'convolutional neural network*':ti,ab,kw OR 'computer aided diagnosis':ti,ab,kw OR 'computer-aided diagnosis':ti,ab,kw OR cad:ti,ab,kw OR 'digital cytology':ti,ab,kw OR 'computational cytology':ti,ab,kw) AND (diagnosis:ti,ab,kw OR classification:ti,ab,kw OR detection:ti,ab,kw OR subtyping:ti,ab,kw OR subtype*:ti,ab,kw OR benign:ti,ab,kw OR malignant:ti,ab,kw) |  | 1,016 results |
| Europe PMC | 11. May. 2026 | ( ("lung cancer" OR NSCLC OR SCLC) AND ( cytology OR cytopathology OR "fine needle aspiration" OR EBUS OR TBNA ) AND ( "artificial intelligence" OR "machine learning" OR "deep learning" OR CNN ) AND ( sensitivity OR specificity OR accuracy OR AUC OR "diagnostic accuracy" ) ) AND (((SRC:MED OR SRC:PMC OR SRC:AGR OR SRC:CBA) NOT (PUB_TYPE:"Review"))) | Article type | 1,509 results |
| OpenAlex | 11. May. 2026 | ("lung cancer" OR "lung carcinoma" OR NSCLC OR SCLC OR adenocarcinoma OR "squamous cell carcinoma")  AND  (cytology OR cytopathology OR "fine needle aspiration" OR EBUS OR TBNA OR sputum)  AND  ("artificial intelligence" OR "machine learning" OR "deep learning" OR CNN OR "computer-aided diagnosis")  AND  (diagnosis OR classification OR subtyping OR benign OR malignant)  NOT  (review OR "systematic review" OR meta-analysis OR bibliometric) | Article type | 148 results |
| PubMed | 11. May. 2026 | ( ( "Lung Neoplasms"[Mesh] OR "lung cancer"[Title/Abstract] OR "lung carcinoma"[Title/Abstract] OR "pulmonary cancer"[Title/Abstract] OR "pulmonary carcinoma"[Title/Abstract] OR NSCLC[Title/Abstract] OR SCLC[Title/Abstract] OR adenocarcinoma[Title/Abstract] OR ADC[Title/Abstract] OR "squamous cell carcinoma"[Title/Abstract] OR SCC[Title/Abstract] ) AND ( "Cytodiagnosis"[Mesh] OR cytology[Title/Abstract] OR cytologic*[Title/Abstract] OR cytopathology[Title/Abstract] OR cytopathologic*[Title/Abstract] OR "fine needle aspiration"[Title/Abstract] OR FNA[Title/Abstract] OR EBUS[Title/Abstract] OR TBNA[Title/Abstract] OR "EBUS-TBNA"[Title/Abstract] OR sputum[Title/Abstract] OR "pleural fluid"[Title/Abstract] OR effusion[Title/Abstract] ) AND ( "Artificial Intelligence"[Mesh] OR "Machine Learning"[Mesh] OR "artificial intelligence"[Title/Abstract] OR "machine learning"[Title/Abstract] OR "deep learning"[Title/Abstract] OR CNN[Title/Abstract] OR "convolutional neural network*"[Title/Abstract] OR "computer aided diagnosis"[Title/Abstract] OR "computer-aided diagnosis"[Title/Abstract] OR CAD[Title/Abstract] OR "digital cytology"[Title/Abstract] OR "computational cytology"[Title/Abstract] ) AND ( diagnosis[Title/Abstract] OR classification[Title/Abstract] OR detection[Title/Abstract] OR subtyping[Title/Abstract] OR subtype*[Title/Abstract] OR benign[Title/Abstract] OR malignant[Title/Abstract] ) ) | - | 369 results |
| Scopus | 11. May. 2026 | TITLE-ABS-KEY ( ( "lung cancer" OR "lung carcinoma" OR "lung neoplasm*" OR NSCLC OR SCLC OR adenocarcinoma OR "squamous cell carcinoma" ) AND ( cytology OR cytopathology OR "cytology specimen*" OR "fine needle aspiration" OR FNA OR EBUS OR "EBUS-TBNA" OR TBNA OR sputum OR "pleural fluid" OR effusion ) AND ( "artificial intelligence" OR "machine learning" OR "deep learning" OR CNN OR "convolutional neural network*" OR "computer-aided diagnosis" OR "digital cytology" ) AND ( sensitivity OR specificity OR accuracy OR AUC OR "area under the curve" OR "diagnostic performance" OR "diagnostic accuracy" ) ) AND ( LIMIT-TO ( DOCTYPE , "ar" ) ) AND ( LIMIT-TO ( LANGUAGE , "English" ) ) | Article type  English language | 440 results |
| Semantic Scholar | 11. May. 2026 | ("lung cancer" OR NSCLC OR SCLC) AND (cytology OR cytopathology) AND ("machine learning" OR "deep learning" OR AI) NOT (review OR meta-analysis) | Article type  English language | 250 results |
| WOS | 11. May. 2026 | "lung cancer" OR "lung carcinoma" OR "pulmonary cancer" OR "pulmonary carcinoma" OR "lung neoplasm*" OR "non-small cell lung cancer" OR NSCLC OR "small cell lung cancer" OR SCLC OR adenocarcinoma OR ADC OR "squamous cell carcinoma" OR SCC (Topic) and cytology OR cytologic* OR cytopathology OR cytopathologic* OR "cytology specimen*" OR "fine needle aspiration" OR FNA OR EBUS OR TBNA OR "EBUS-TBNA" OR sputum OR "pleural fluid" OR effusion (Topic) and "artificial intelligence" OR "machine learning" OR "deep learning" OR CNN OR "convolutional neural network*" OR "computer-aided diagnosis" OR "computer aided diagnosis" OR CAD OR "digital cytology" OR "computational cytology" (Topic) and diagnosis OR classification OR detection OR subtyping OR subtype* OR benign OR malignant (Topic) and Article (Document Types) and English (Languages) | Article type  English language | 206 results |

WOS, Web of Science

## Supplementary Table S2. Eligibility criteria.

| **Domain** | **Inclusion criteria** | **Exclusion criteria** | **Operational notes** |
| --- | --- | --- | --- |
| Population | Human cytology specimens are evaluated for suspected or confirmed lung cancer. | Non-lung primary tumors only; animal/in-vitro-only studies. | NR |
| Specimen | Cytology specimens such as EBUS-TBNA, FNAC, sputum, bronchial washing/brushing, BAL, pleural fluid, and related lung cytology material. | Histology-only or radiology-only studies with no cytology specimen data. | NR |
| Index test | Artificial intelligence, machine learning, or deep learning models applied to cytology images, slides, cells, patches, or specimen-level features. | Manual cytology only; non-AI rule-based methods without classification performance. | NR |
| Target condition | Benign vs malignant lung cytology diagnosis and/or ADC/SCC/SCLC subtyping. | Tasks unrelated to diagnosis/subtyping, e.g., prognosis only. | NR |
| Reference standard | Histopathology, expert cytopathology consensus, clinical follow-up, or adjudicated standard. | No reference standard or unverifiable labels. | NR |
| Outcomes | Extractable 2x2 data or enough information to reconstruct TP/FP/FN/TN; other metrics recorded separately. | No diagnostic performance data. | NR |
| Study type | Original diagnostic/classification studies, full text, peer-reviewed, or accepted preprint if eligible by protocol. | Reviews, editorials, protocols, and abstracts without full data. | NR |
| Language/date | As specified in the protocol. | As specified in the protocol. | NR |

EBUS-TBNA, endoscopic bronchial ultrasound-guided transbronchial needle aspiration; FNAC, fine-needle aspiration cytology; BAL, bronchoalveolar lavage; ADC, adenocarcinoma; SCC, squamous cell carcinoma; SCLC, small cell lung cancer; TP, true positive; FP, false positive; FN, false negative; TN, true negative

## Supplementary Table S3. Study selection and PRISMA-DTA flow counts.

| **Flow stage** | **Count** | **Linked source** | **Notes** |
| --- | --- | --- | --- |
| Records identified from databases | 0 | Collected the Excel file, Search_Log sheet | No bibliographic database search was performed in this extraction-only step. |
| Records identified from other sources | 32 | Collected the Excel file, Search_Log sheet | full-text papers across 7 extraction batches. |
| Duplicates removed | 0 | Collected the Excel file, Search_Log sheet | No duplicate papers are recorded in the workbook. |
| Records screened | 32 | Collected the Excel file, Search_Log sheet | Calculated after deduplication. |
| Records excluded at title/abstract | 0 | Collected the Excel file, Search_Log sheet | No title/abstract screening step in this extraction-only workbook. |
| Reports sought for retrieval | 32 | Collected the Excel file, Search_Log sheet | All full texts were available in the extraction workbook. |
| Reports not retrieved | 0 | Collected the Excel file, Search_Log sheet | All papers were retrievable. |
| Reports assessed for eligibility | 32 | Collected the Excel file, Study_Characteristics sheet | All full texts have been entered in the characteristics table. |
| Full-text reports excluded | 0 | S4 | No full text excluded from qualitative extraction; rows without reliable 2x2 data are marked No/Check in DTA/meta tables. |
| Studies included in the qualitative synthesis | 32 | Collected the Excel file, Study_Characteristics sheet | Count from non-empty Study_Characteristics rows. |
| Studies included in the DTA meta-analysis | 20 | Collected the Excel file, Meta_Analysis_Input sheet | Unique Study_ID values with at least one Include=Yes row. |
| Benign-malignant DTA rows included | 18 | Collected the Excel file, BM_DTA / Meta_Analysis_Input sheets | Rows with Include=Yes and benign-malignant analysis type. |
| Subtype DTA rows included | 15 | Collected Excel file, Subtyping_DTA / Meta_Analysis_Input sheets | Rows with Include=Yes and subtyping analysis type. |

DTA, diagnostic test accuracy; BM, benign-malignant

## Supplementary Table S4. Characteristics of included studies.

| **Study ID** | **Country/region** | **Design/setting** | **Cytology specimen and preparation** | **Population and sample size** | **Target task(s)** | **Reference standard** | **Reference** |
| --- | --- | --- | --- | --- | --- | --- | --- |
| Ai et al., 2022 | China | Retrospective diagnostic model development/validation \| Single center: Zhongshan Hospital, Fudan University | Bronchoscopy ROSE slides from TBNA, TBLB with/without EBUS-GS, and TBB \| Cytological WSI from rapid H&E-stained ROSE smear; one slide per patient \| Rapid H&E | Patients receiving bronchoscopy examinations from Jan 2017 to Dec 2019; one ROSE slide selected per patient. Training/validation required definitive pathology, clinical information, and acceptable scan quality; testing used a comprehensive final diagnosis. \| patients: 627 \| specimens/data: 627 ROSE slides; 739,582 ROSE patches (training 374 slides, validation 91, testing 162) | Malignant vs benign | Comprehensive clinical diagnosis with pathology/clinical data | ^1^ |
| Chen et al., 2020 | Indonesia | Unclear/retrospective image dataset \| Single center: RSUD Dr. Soetomo Surabaya | Pleural fluid/FNAB cytology images \| ThinPrep slide cytology images; augmentation performed \| Not reported | Cytology images from lung/pleural fluid examination classified as cancer versus normal; exact patient/sample count not reported. \| patients: Not reported \| specimens/data: Not reported; article states dataset was limited and augmented | Cancer/abnormal vs normal | Cytology label; details not reported; Cytology label; details NR | ^2^ |
| Chen et al., 2021a | Taiwan | Prospective/observational diagnostic AI study \| Two National Taiwan University sites (Cancer Center and Hsin-Chu Hospital) | EBUS-TBB and EBUS-TBNA imprint ROSE cytologic images \| Imprint smears on glass slide without coverslip; digital microscope images cropped to patches \| Hemacolor rapid stain | 97 patients undergoing 104 EBUS procedures; 70 peripheral pulmonary lesions and 34 mediastinal/hilar lesions; ROSE images captured during procedure. \| patients: 97 \| specimens/data: 499 cytologic images; 7,486 patches; test set 66 images/990 patches/14 patients | Malignant vs benign | Formal cytopathologic results | ^3^ |
| Chen et al., 2021b | China | Retrospective case-control/biobank diagnostic study \| Single center: Zhejiang Cancer Hospital biobank | Pleural effusion supernatant \| Centrifuged supernatant; FT-NIR spectra captured in triplicate | 82 pleural effusion samples: 47 malignant pleural effusions from lung adenocarcinoma with pleural metastases and 35 benign pleural effusions from tuberculosis/tuberculous pleurisy. \| patients: 82 \| specimens/data: 82 pleural effusion samples; train 62 (36 MPE/26 BPE), test 20 (11 MPE/9 BPE) | Malignant pleural effusion vs benign pleural effusion | MPE cytology/histology; BPE TB culture/clinical diagnosis | ^4^ |
| Chen et al., 2024 | China | Retrospective observational diagnostic AI study \| Single center: Shanghai Chest Hospital | ROSE slides from TBNA, TBLB, and TBB of lung lesions or intrathoracic lymphadenopathy \| Stamped/smeared ROSE slides scanned as WSI; paired serum tumor markers used as model inputs \| Diff-Quik | ROSE slides of patients with lung lesions or intrathoracic lymphadenopathy undergoing transbronchial sampling; included only diagnostic procedures and WSIs whose cytology label matched the final diagnosis. \| patients: Not reported separately; 811 WSIs \| specimens/data: 811 WSIs; 16,621,134 patches; train 481, validation 156, test 174 | Malignant vs benign; ADC/LUAD vs rest; SCC/LUSC vs rest; SCLC vs rest | Final transbronchial sampling diagnosis with immunohistochemistry/follow-up | ^5^ |
| Ganesh et al., 2022 | Singapore | Pilot proof-of-concept diagnostic ML study \| Single-center clinical pleural fluid sample collection, National University Hospital / A*STAR Singapore | Pleural fluid \| Centrifuged pleural fluid supernatant; 5 uL deposited on silver-coated silicon nanopillar SERS substrate \| Label-free SERS; no cytology stain | 34 pleural-effusion subjects: 15 lung cancer, 7 other cancers, and 12 non-cancer controls; primary lung cancer vs control analysis used n=12 lung cancer and n=11 controls. \| patients: 34 \| specimens/data: 34 pleural fluid samples; lung cancer vs control analysis n=23; all-cancer vs control analysis n=34; thousands of SERS spectra acquired per group. | Lung cancer-associated pleural effusion vs control effusion; Any cancer-associated pleural effusion vs control effusion | Clinical diagnosis of lung cancer vs non-cancer control; Clinical diagnosis of cancer vs non-cancer control; Clinical diagnosis of lung cancer vs control effusion | ^6^ |
| Giarnieri et al., 2025 | Italy | Retrospective pilot diagnostic AI study \| Single center: Cytopathology Unit, Sant'Andrea University Hospital, Sapienza University of Rome | Pleural effusion cytology \| Standard cytology preparations; manually annotated single cells \| Papanicolaou | 78 pleural effusion cases from Jan 2022 to Dec 2023: 40 lung adenocarcinoma malignant pleural effusions and 38 benign pleural effusions; 969 images with 6,390 cell annotations. \| patients: 78 \| specimens/data: 969 raw images; 3,260 adenocarcinoma cell annotations and 3,130 mesothelial cell annotations; train/validation/test split 80/10/10. | Adenocarcinoma cells vs mesothelial cells | Cytopathology diagnosis with IHC confirmation/rule-out of lung adenocarcinoma MPE; Cytopathology and immunohistochemical confirmation/rule-out | ^7^ |
| Gong et al., 2025 | China | Retrospective diagnostic AI model evaluation \| Single center: Lishui Municipal Central Hospital, Zhejiang, China | Respiratory cytology samples from lung lesions / intrathoracic sampling \| Diff-Quik-stained slides scanned as WSIs; high-magnification still images selected for testing \| Diff-Quik | 739 respiratory specimens with on-site diagnoses were collected; confirmed case set described 96 SCC, 64 adenocarcinoma, 6 NSCLC-NOS, 50 SCLC, 1 carcinoid, and 20 normal cases. The test set used 116 images across six categories. \| patients: 739 respiratory specimens; 237 confirmed/normal cases described \| specimens/data: 116 test images: 8 carcinoid, 5 NSCLC-NOS, 16 SCLC, 33 normal, 20 SCC, and 34 adenocarcinomas. | Six-class respiratory cytology diagnosis; binary malignant/benign not extractable | Biopsy/cell block/IHC confirmation and clinical/imaging normal adjudication; Biopsy/cell block/IHC for malignancies; biopsy/clinical/imaging adjudication for normal cases | ^8^ |
| Gonzalez et al., 2020 | USA | Retrospective pilot diagnostic algorithm study \| Single institution archival lung FNA cases, University of Pittsburgh Medical Center / University of Miami authorship | Lung fine-needle aspiration cytology smears with cell block/core biopsy material \| Direct FNA smears and cell block/core biopsy sections; tumor regions annotated in QuPath and exported as tiles \| Diff-Quik, Papanicolaou, and H&E | 40 archival lung FNA cases of high-grade neuroendocrine carcinoma: 17 SCLC, 13 LCNEC, and 10 mixed/unclassifiable. Cases randomized into training and test groups. \| patients: 40 \| specimens/data: 114 slides reported in abstract; methods also state 117 slides/464,378 tiles. Training: 20 cases (11 LCNEC, 9 SCLC); test: 20 cases (2 LCNEC, 8 SCLC, 10 mixed/unclassifiable). | LCNEC vs rest | Original cytology diagnosis with IHC confirmation of neuroendocrine differentiation; Original cytology diagnosis with IHC neuroendocrine confirmation | ^9^ |
| Kim et al., 2023 | South Korea | Retrospective multicenter diagnostic AI study \| Nationwide Korean respiratory cytology image set from >200 institutions plus tertiary/major hospitals | Sputum and bronchial washing respiratory cytology \| Conventional smear and liquid-based cytology; extended Z-stack image generation, color normalization, patch extraction \| H&E and Papanicolaou | 1,273 patients/WSIs from sputum and bronchial washing specimens; 716 lung malignancies and 557 benign lesions. The dataset included conventional smears and liquid-based cytology with H&E or PAP staining. \| patients: 1273 \| specimens/data: 30,590 image patches from 1,273 WSIs; training 27,362 patches/1,146 WSIs; validation 3,228 patches/126 WSIs; testing/human comparison 1,272 patches. | Lung cancer vs benign cells | Expert patch labels based on corresponding histopathology/cytology and review; Corresponding histopathology and expert cytology patch review | ^10^ |
| Kim et al., 2025 | South Korea | Retrospective diagnostic model development/validation with multicenter/nationwide dataset and external validation \| Open AI Dataset Project for Cytopathology and Korean Society of Cytopathology Quality Assurance Program | Pleural fluid cytology \| Whole-slide cytology images; z-stacked extended-depth-of-field scanning \| Papanicolaou and H&E | Pap- and H&E-stained pleural fluid WSIs classified as benign or malignant for lung cancer; malignant slides were histologically confirmed and expert reviewed. \| patients: Not reported; 885 development WSIs plus 93 external validation WSIs \| specimens/data: 885 WSIs (576 benign/309 malignant); WSI-level analysis used 837 WSIs; external validation 93 WSIs; patch model used 30,801 patches | Malignant effusion vs benign | Histology-confirmed malignant cases and expert-reviewed cytology patch labels; Histologically confirmed malignant cases and board-certified pathologist review; Histology-confirmed malignant cases and expert cytology patch review; Histology-confirmed malignant cases and board-certified pathologist review | ^11^ |
| Lai et al., 2025 | Taiwan | Proof-of-concept optical/hyperspectral feature-extraction study \| Taiwan hospital pleural-effusion cytology samples under IRB-approved retrospective/anonymized protocol | Pleural effusion cytology \| Pleural effusion centrifugation/Ficoll-Paque mononuclear-cell isolation; cytocentrifuge slides; methanol fixation \| Giemsa | Pleural effusion cells categorized as normal, non-normal/reactive, or malignant by a board-certified cytologist \| patients: Not reported \| specimens/data: 24 cells/ROIs at the nucleus site; each ROI 10×10 pixels | Malignant vs non-malignant/reactive cells | Board-certified cytologist cell classification | ^12^ |
| Lan et al., 2024 | China | Deep learning segmentation model development with internal testing and multicenter external validation \| Xijing Hospital internal cohort; Tianjin Medical University General Hospital and First Affiliated Hospital of Chongqing Medical University external images | EBUS-TBNA ROSE cytopathology/pathology sections \| ROSE-stained pathological sections; cancer-cell cluster masks drawn by pathologists \| ROSE stain (rapid on-site cytopathological evaluation; exact stain not separately specified) | Cytologically stained ROSE images of lung cancer cell clusters from EBUS-TBNA-related specimens; pathologist-annotated cancer-cell areas \| patients: Not reported \| specimens/data: 130 internal ROSE cytology images (91 train, 26 validation, 13 test) augmented to 36,400/10,400/5,200 image patches; 20 external images | Cancer-cell cluster segmentation; not case-level B/M diagnosis | Pathologist segmentation masks and labels; Pathologist segmentation masks | ^13^ |
| Lemieux et al., 2023 | USA/Canada | Multisite sputum-based diagnostic classifier development/validation with independent instrument validation \| Atlantic Health System, Mt. Sinai Hospital, Radiology Associates of Albuquerque, South Texas Veterans Healthcare System, and Waterbury Pulmonary Associates; analysis by bioAffinity | Induced sputum \| Single-cell suspensions from induced sputum; automated flow cytometry with viability, lineage, and porphyrin labeling \| FVS510 viability dye, TCPP porphyrin, and antibody panel (CD45, CD66b/CD3/CD19, CD206, Pan-CK, EpCAM) | High-risk smoker non-cancer group and physician-suspected lung-cancer group with cancer confirmed by biopsy after sputum collection \| patients: 216 enrolled; 150 LSRII validation samples and 32 Navios independent validation samples passed QC \| specimens/data: 150 LSRII samples (28 cancer/122 non-cancer); 32 Navios EX samples (6 cancer/26 non-cancer); induced sputum over 3 days | Lung cancer vs non-cancer; Lung cancer vs non-cancer in small-nodule subset | Biopsy-confirmed cancer; imaging/clinical non-cancer classification; Cancer confirmed by biopsy; non-cancer status from imaging/clinical classification | ^14^ |
| Liu et al., 2023 | China | Deep learning object-detection model development with clinical cross-validation \| Peking University First Hospital and Anhui Chest Hospital | Bronchoalveolar lavage fluid (BALF) cells recovered on PERFECT/BALFilter filters \| PERFECT filter recovery of exfoliated tumor cells; in situ HE staining; large-field image slicing into 256×256 blocks \| Hematoxylin-eosin (HE) | BALF from lung-cancer-suspected patients; one positive case used for image-block training/validation and 24 additional clinical cases used for BALFilter Reader evaluation \| patients: 24 clinical validation cases (18 histopathology-positive, 6 negative) plus one positive case for training/validation image blocks \| specimens/data: 300 annotated image blocks for model development (240 train, 60 validation); 24 large-field clinical BALFilter images for validation | Lung cancer vs non-cancer | Histopathology results are the gold standard | ^15^ |
| Taher et al., 2015 | UAE/Japan | Retrospective CAD development/validation on image dataset \| Tokyo Center of Lung Cancer sputum image database; analysis by Khalifa University, UAE | Sputum cytology color images \| Sputum cell extraction, mean-shift segmentation of nuclei/cytoplasm, feature extraction \| Papanicolaou standard stain | Database of 100 sputum color images from different patients; pathologist-identified cells used as gold standard for CAD evaluation. \| patients: Not reported; authors state images from different patients \| specimens/data: 100 sputum color images | Benign vs malignant sputum cells | Pathologist-identified cells/diagnosis | ^16^ |
| Taher et al., 2023 | UAE/Japan | Retrospective image-processing/CAD development-validation \| Tokyo Center of Lung Cancer image dataset; analysis by Khalifa University, UAE | Sputum cytology color images \| Bayesian histogram segmentation, 8-connected component region detection, feature extraction/rule-based CAD \| Papanicolaou standard stain | 100 sputum images, each with at least one sputum cell; manual ground-truth masks for segmentation and pathologist comparison for CAD diagnosis. \| patients: Not reported \| specimens/data: 100 sputum images; 65 training images and 35 test images for Bayesian segmentation; 100 images for CAD-pathologist comparison | Cancerous vs normal sputum cells | Experimented pathologist diagnosis | ^17^ |
| Tanaka et al., 2025 | Japan | Retrospective DCNN model development/validation \| Single center: Kyorin University Hospital, Tokyo, Japan | Liquid-based cytology from scraped surgical tumor and normal parenchymal specimens \| BD SurePath LBC slides from scraped surgical specimens \| Papanicolaou stain | 37 surgical lung cancer cases diagnosed as ADC or SQCC plus 8 normal parenchymal controls; slides scanned as WSI and patch-annotated. \| patients: 45 \| specimens/data: 45 LBC slides; 9,141 final patches (2,737 N, 4,756 ADC, 1,648 SQCC) | Malignant (ADC+SQCC) vs normal; ADC vs rest; SCC/SQCC vs rest | Final surgical pathology and cytopathologist patch annotation; Final surgical pathology diagnosis; Final surgical pathology with IHC/mucin stains; Final surgical pathology diagnosis with IHC/mucin stains where needed | ^18^ |
| Tang et al., 2024 | China | Retrospective model development with held-out external test set \| Single center: Lishui Central Hospital, Zhejiang, China | Fine-needle aspiration cytology and bronchoscopic brush cytology images \| Diff-Quik-stained rapid cytopathology images captured at fixed field of view \| Diff-Quik | 209 digital cytopathology images/cases: 104 adenocarcinoma and 105 squamous cell carcinoma, collected 2021–2023. \| patients: 209 \| specimens/data: 209 images/cases; training 169 images augmented to 676; external test 40 images (20 ADC, 20 SCC) | ADC/SCC binary task vs rest | Pathology diagnosis of ADC or SCC | ^19^ |
| Teramoto et al., 2017 | Japan | Retrospective DCNN image classification with cross-validation \| Fujita Health University, Japan | Exfoliative/interventional cytology under bronchoscopy or CT-guided FNAC \| BD SurePath liquid-based cytology; cropped/resized microscopic images \| Papanicolaou stain | 76 cases of lung cancer cytology: 40 ADC, 20 SCC, 16 SCLC; cytology obtained by bronchoscopy or CT-guided FNAC and confirmed by histopathology/IHC. \| patients: 76 \| specimens/data: 298 original images (82 ADC, 125 SCC, 91 SCLC), augmented during training | ADC vs rest; SCC/SQCC vs rest; SCLC vs rest | Histopathology and IHC diagnosis; Histopathology and immunohistochemistry diagnosis | ^20^ |
| Teramoto et al., 2019 | Japan | Retrospective DCNN model development/validation \| Single center: Fujita Health University, Japan | Bronchoscopy or CT-guided FNAC lung cytology \| BD SurePath liquid-based cytology \| Papanicolaou | 46 cases: 18 benign and 28 malignant; interventional cytology under bronchoscopy or CT-guided FNAC with histopathology/IHC diagnosis. \| patients: 46 \| specimens/data: 417 microscopic images; 621 patches (306 benign/315 malignant); augmented to 60,000 patches | Malignant vs benign lung cytology patches; Malignant vs benign lung cytology cases | Histopathology/IHC diagnosis with cytopathologist patch review; Histopathology/IHC diagnosis | ^21^ |
| Teramoto et al., 2020 | Japan | Retrospective DCNN/GAN model development/validation \| Single center: Fujita Health University, Japan | Bronchoscopy or CT-guided FNAC lung cytology \| BD SurePath liquid-based cytology \| Papanicolaou | 60 patients: 25 benign and 35 malignant; lung cytology collected by bronchoscopy or CT-guided FNAC with histopathology/IHC reference. \| patients: 60 \| specimens/data: 511 microscopic images; 793 patches (391 benign/402 malignant); 620 images in reported 3-fold DTA table after validation holdout | Malignant vs benign lung cytology patches | Histopathology/IHC diagnosis with cytopathologist patch review | ^22^ |
| Teramoto et al., 2022 | Japan | Retrospective DCNN model comparison/validation \| Fujita Health University, Japan | Exfoliative/interventional cytology under bronchoscopy or CT-guided FNAC \| BD SurePath liquid-based cytology \| Papanicolaou | 55 lung cancer cases: 36 adenocarcinoma, 14 squamous cell carcinoma, and 5 small cell carcinoma; cytology from exfoliative or interventional sampling under bronchoscopy or CT-guided FNAC. \| patients: 55 \| specimens/data: 289 original cropped images in Table 1 (75 ADC, 130 SCC, 84 SCLC); augmented to 5,000 images per class per fold | ADC vs rest; SCC/SQCC vs rest; SCLC vs rest | Histopathology and IHC diagnosis | ^23^ |
| Tondo et al., 2025 | Italy | Retrospective multicentre diagnostic performance study \| Multicentre interventional pulmonology/pathology units in Foggia and Taranto, Italy | EBUS-TBNA, EUS-B-FNA, transthoracic echo-guided needle biopsy, and transbronchial biopsy ROSE images \| Air-dried ROSE smear images; static microscope fields shared digitally \| May-Grünwald Giemsa | 277 ROSE-valid biopsy sample images from patients evaluated for suspected primary or secondary pulmonary neoplasm; 195 positive, 57 negative, and 25 doubtful images. \| patients: Not reported \| specimens/data: 277 static ROSE images; positive/negative AI analysis n=252; doubtful-inclusive analysis n=277 | Lung cancer-positive vs negative ROSE images | Definitive histological report with possible immunohistochemistry | ^24^ |
| Wang et al., 2025a | China | Retrospective deep-learning segmentation/classification study \| Single center: China-Japan Friendship Hospital, Beijing, China | Scratch-imprint cytology, pleural fluid cytology, and bronchoalveolar lavage fluid cytology \| Scratch-imprint cytology fixed in 95% ethanol; other cytology test sets \| H&E for SIC; additional cytology stains not fully specified | 405 lung lesion SIC samples and 205 normal lung tissue SIC samples; tissue collected during intraoperative pathology consultation from Feb 2021 to Apr 2022. \| patients: 610 \| specimens/data: 4,610 SIC images (2,279 malignant/2,331 non-malignant); additional SIC test set 311 images, pleural fluid 120, BAL 120 | Malignant vs benign/non-malignant lung cytology images | Postoperative FFPE histopathology gold standard | ^25^ |
| Wang et al., 2025b | China | Clinical diagnostic model development/validation with internal five-fold cross-validation \| Single center: Second Affiliated Hospital of Fujian Medical University, Quanzhou, China | Pleural effusion supernatant plus serum CEA \| Pleural effusion centrifuged; supernatant mixed with Ag nanoparticles and dried on Al foil; serum CEA digitized at >5 ng/mL \| Not applicable | Pleural effusion samples from 28 non-cancer patients, 15 lung cancer patients, and 10 other cancer patients with diagnoses confirmed by histopathologic or cytologic examination. \| patients: 53 \| specimens/data: 53 pleural effusion samples; 212 spectra if four spectra per sample; serum CEA levels digitized | Lung cancer vs non-cancer pleural effusion; Other cancers vs non-cancer pleural effusion; Lung cancer vs other cancers | Histopathologic or cytologic examination confirmed clinical group | ^26^ |
| Wilbur et al., 2015 | International multicenter (USA/Canada/Greece/Israel) | Interim/preliminary clinical diagnostic feasibility study \| Sputum samples from 7 sites; LuCED/Cell-CT testing by VisionGate | Sputum cytology \| CytoLyt fixation, pooled 3-day sputum, hematoxylin staining, epithelial-cell enrichment by keratin/CD45 FACS, optical medium for Cell-CT \| Hematoxylin | 91 adequate sputum specimens from 49 patients with confirmed lung cancer and 42 patients with nonmalignant lung pathology, benign tumors, or no known pulmonary pathology. \| patients: 91 \| specimens/data: 91 pooled sputum specimens; three morning cough sputa pooled per patient | Lung cancer vs nonmalignant/no known malignancy | Confirmed lung cancer vs nonmalignant lung pathology/benign tumor/no known pulmonary pathology at last follow-up; Known lung malignancy vs nonmalignant/no known pulmonary pathology | ^27^ |
| Xie et al., 2022 | China | Retrospective single-center WSI diagnostic AI study \| Shanghai Pulmonary Hospital, Tongji University | Pleural effusion liquid-based cytology \| CytoRich Non-gyn liquid-based cytology; WSI patches cropped 512×512 and resized 256×256 \| H&E | 404 pleural-effusion liquid-based cytology WSIs from benign and pulmonary adenocarcinoma cases; 234 benign and 170 malignant slides; one highest-quality slide selected per patient. \| patients: 404 \| specimens/data: 404 WSIs; 266 training, 78 validation, 60 test; 365,752 test patches | Malignant vs benign pleural effusion cytology | Pathology report / cytological morphology ± matched cell block with IHC; Pathological report; 72% with cytology plus matched cell block/IHC and 28% morphology alone | ^28^ |
| Yan et al., 2024 | China | Retrospective single-center model development with temporal testing group \| Tangdu Hospital, Air Force Medical University, Xi'an, China | Transbronchial biopsy ROSE cytology images \| Biopsy specimens smeared on sterile slides, air-dried, Diff-Quik stained; images resized to 1024×1024 for training \| Diff-Quik | 6357 ROSE cytology images from 721 patients undergoing transbronchial biopsy, Jan–Jul 2023; AI-develop group, Jan–May, and AI-test group, Jun–Jul. \| patients: 721 \| specimens/data: 6357 ROSE images; 4106 training, 544 validation, 526 internal test, 1181 temporal/external test | Cancer image vs non-cancer image; Lung cancer vs non-cancer | Experienced cytopathologist annotation accepted only when consistent with final pathological diagnosis; Final pathological diagnosis | ^29^ |
| Zhang et al., 2026 | China | Clinical diagnostic model development with internal stratified test set \| First People's Hospital of Yunnan Province, Kunming, China | Lung cytological smears/brush cytology smears \| Cytological smears measured by label-free Raman spectroscopy after storage at −80 °C and thawing \| Label-free Raman; no stain reported | Cytological smear samples from 81 non-cancer patients with radiologically detected pulmonary abnormalities and 14 histopathologically confirmed primary lung cancer patients (8 ADC, 4 SCC, 2 SCLC). \| patients: 95 \| specimens/data: 950 Raman spectra (10 points/sample); stratified 8:2 spectrum-level split; independent test set 190 spectra | Lung cancer vs non-cancer; ADC/LUAD vs rest; SCC/SQCC vs rest; SCLC vs rest | Histopathologic confirmation of primary lung cancer vs benign/non-cancer disease; Histopathologic subtype for 14 lung cancers (8 ADC, 4 SCC, 2 SCLC); Histopathological confirmation of lung cancer or other diseases | ^30^ |
| Zhao et al., 2025a | China | Retrospective single-center diagnostic model development/evaluation \| Second Affiliated Hospital of Zunyi Medical University; pulmonary-lesion small biopsy/ROSE workflow | ROSE cytology from small biopsy/direct tissue imprint; serum tumor markers \| Direct tissue imprint cytology using crush smears/touch imprints; residual tissue submitted for histopathology \| Not reported for ROSE stain | 281 pulmonary-lesion patients retrospectively analyzed; AI core set was 156 matched cases with ROSE cytology images and serum markers (31 benign, 125 malignant) \| patients: 156 core AI patients; 281 total, including non-ROSE adequacy comparator \| specimens/data: 156 matched ROSE/serum cases; 995 pathologist-annotated ROIs | Malignant lung lesion vs benign; ADC/LUAD vs rest; SCC/LUSC vs rest; SCLC vs rest | Final histopathology with clinical/IHC confirmation | ^31^ |
| Zhao et al., 2025b | China | Retrospective model development with same-center validation and two-center external validation \| The First Affiliated Hospital, Zhejiang University School of Medicine; BAL during routine bronchoscopy at Qingchun and Yuhang campuses | Bronchoalveolar lavage fluid cytology; Pap-stained BAL slides; FNAC slides used only for additional cell-level generalization test \| BALF sedimentation liquid-based preparation; WSI scanning after Pap staining \| Papanicolaou | Four cohorts: 34-patient single-cell training/validation cohort; 156-patient discovery cohort; 158-patient same-center validation cohort; 141-patient external validation cohort \| patients: 489 total across all cohorts; DTA after exclusions: 128 discovery, 128 validation, 118 external \| specimens/data: 580 scDNA-Seq-confirmed ETCs + 1106 benign cells; DTA: 374 BALF specimens after clinical exclusions | Lung cancer vs control/benign pulmonary disease | Surgical/biopsy histopathology for malignant; negative pathology/cytology plus ≥6-month follow-up and alternative etiology for benign; Malignant diagnosis by surgical or biopsy histopathology; benign diagnosis by negative pathology/cytology, ≥6-month follow-up, and alternative etiology | ^32^ |

ROSE, rapid on-site evaluation; TBNA, transbronchial needle aspiration; TBLB, transbronchial lung biopsy; EBUS-GS, endobronchial ultrasound with guide sheath; TBB, transbronchial biopsy; WSI, whole-slide image; H&E, hematoxylin and eosin; FNAB, fine-needle aspiration biopsy; FT-NIR, Fourier transform near-infrared; MPE, malignant pleural effusion; BPE, benign pleural effusion; TB, tuberculosis; LUAD, lung adenocarcinoma; LUSC, lung squamous cell carcinoma; IHC, immunohistochemistry; ML, machine learning; SERS, surface-enhanced Raman spectroscopy; IQR, interquartile range (not in text but noted as common adjacent term); NSCLC-NOS, non-small cell lung cancer not otherwise specified; LCNEC, large cell neuroendocrine carcinoma; FVS, fixable viability stain; TCPP, meso-tetra(4-carboxyphenyl)porphine; EpCAM, epithelial cell adhesion molecule; QC, quality control; CAD, computer-aided diagnosis; DCNN, deep convolutional neural network; BD, Becton Dickinson; LBC, liquid-based cytology; SQCC, squamous cell carcinoma; CT, computed tomography; GAN, generative adversarial network; EUS-B-FNA, endoscopic ultrasound with bronchoscope-guided fine-needle aspiration; CEA, carcinoembryonic antigen; FACS, fluorescence-activated cell sorting; ROI, region of interest; scDNA-Seq, single-cell DNA sequencing; ETC, epithelial tumor cell

## Supplementary Table S5. AI model and dataset characteristics.

| **Study ID** | **Model ID** | **Algorithm/architecture** | **Input unit** | **Annotation and ground truth level** | **Training/validation/test/external n** | **Threshold rule** | **Validation design** | **Code/data availability** | **Reference** |
| --- | --- | --- | --- | --- | --- | --- | --- | --- | --- |
| Ai et al., 2022 | AI_ROSE_DenseNet169 | CNN: DenseNet-169 patch classifier with WSI decision logic | Whole-slide image/patch | Two junior cytopathologists annotated patches; a senior cytopathologist confirmed annotations \| Histopathology for training/validation; comprehensive clinical diagnosis for test | Train: 374 patients/slides (279,322 patches; 9,054 annotated) \| Validation: 91 patients/slides (176,933 patches; 4,530 annotated) \| Test: 162 patients/slides (283,327 patches) \| External: 0 | Optimized: malignant patch probability ≥0.81 and >6 malignant patches per WSI | Internal training/validation split plus separate internal testing group | Code: No \| Data: No | ^1^ |
| Chen et al., 2020 | EfficientNet_GBRT_CNN | CNN: EfficientNet; hyperparameter optimization by Gaussian Process and Gradient Boosted Regression Tree | Microscopy cytology image | Not reported \| Cancer vs normal cytology labels | Train: Not reported \| Validation: Not reported \| Test: Not reported \| External: 0 | Not reported | Train-test split; exact split/sample count not reported | Code: Not reported \| Data: Not reported | ^2^ |
| Chen et al., 2021a | ResNet101_BM | CNN: ResNet101 for benign/malignant classification; HRNet for malignant cell semantic segmentation | Patch, image, and patient | Malignant areas labeled with LabelMe; final cytopathologic results used for image labels \| Formal cytopathologic results | Train: 70% of participants/images; 7,486 generated patches total before split \| Validation: 15% of participants/images \| Test: 15%: 66 images, 990 patches, 14 patients \| External: 0 | Softmax output >0.99 for malignant image; patient-level majority vote | Internal participant-level split | Code: Not reported \| Data: Available on request; not public | ^3^ |
| Chen et al., 2021b | NIRS_PLS | Classical machine learning: Partial least squares | Specimen-level near-infrared spectrum | Clinical/pathologic diagnosis of MPE/BPE \| Cytology/histology for MPE; bacterial culture/clinical TB diagnosis for BPE | Train: 62 samples with repeated fivefold CV \| Validation: Cross-validation within training set \| Test: 20 samples \| External: 0 | Model default/optimized by cross-validation | Internal random train/test split | Code: Not reported \| Data: Not reported | ^4^ |
|  | NIRS_RF | Classical machine learning: Random forest | Specimen-level near-infrared spectrum | Clinical/pathologic diagnosis of MPE/BPE \| Cytology/histology for MPE; bacterial culture/clinical TB diagnosis for BPE | Train: 62 samples with repeated fivefold CV \| Validation: Cross-validation within training set \| Test: 20 samples \| External: 0 | mtry optimized by cross-validation | Internal random train/test split | Code: Not reported \| Data: Not reported |  |
|  | NIRS_SVM | Classical machine learning: Linear-kernel SVM; top-50 feature SVM-RFE variant also reported | Specimen-level near-infrared spectrum | Clinical/pathologic diagnosis of MPE/BPE \| Cytology/histology for MPE; bacterial culture/clinical TB diagnosis for BPE | Train: 62 samples with fivefold CV \| Validation: Cross-validation within training set \| Test: 20 samples \| External: 0 | Linear kernel, cost 5; model default decision boundary | Internal random train/test split | Code: Not reported \| Data: Not reported |  |
|  | NIRS_GBM | Classical machine learning: Gradient boosting machine | Specimen-level near-infrared spectrum | Clinical/pathologic diagnosis of MPE/BPE \| Cytology/histology for MPE; bacterial culture/clinical TB diagnosis for BPE | Train: 62 samples with repeated fivefold CV \| Validation: Cross-validation within training set \| Test: 20 samples \| External: 0 | ntrees/interactions/shrinkage/minobs optimized | Internal random train/test split | Code: Not reported \| Data: Not reported |  |
| Chen et al., 2024 | HMLCS_ROSE_Serum | Deep learning/attention MIL: ResNet50 ImageNet feature extractor + attention aggregation + fully connected hierarchical classifiers + serum biomarkers | WSI patches plus serum biomarkers | Experienced cytopathologist labeled WSIs under guidance of final diagnoses; accepted only if WSI label matched final diagnosis. \| Final transbronchial sampling diagnosis with immunohistochemistry; follow-up/adjudication for nonspecific benign cases | Train: 481 WSIs \| Validation: 156 WSIs \| Test: 174 WSIs \| External: 0 | Model output/default threshold not explicitly prespecified | Internal random train/validation/test split | Code: No \| Data: No | ^5^ |
| Ganesh et al., 2022 | SERS_PLSDA_Lung_vs_Control | Classical machine learning: Partial least squares-discriminant analysis binary classifier; PCA/LDA/ULDA also discussed | Pleural-fluid SERS spectrum | Clinical study group labels \| Clinical diagnosis of lung cancer-associated pleural effusion vs control effusion | Train: ~4000 spectra per group from n=12 lung cancer and n=11 controls \| Validation: Stratified 10-fold cross-validation \| Test: No independent subject-level test set \| External: 0 | PLS-DA binary decision boundary | Internal stratified 10-fold cross-validation | Code: No \| Data: Main article/supporting information | ^6^ |
| Giarnieri et al., 2025 | YOLOv8_ADK_MS | Object detection/instance segmentation: YOLOv8 instance segmentation model | Cell annotation/cytology image | Manual annotations of adenocarcinoma and mesothelial cells \| Cytopathology diagnosis supported by immunohistochemistry for lung adenocarcinoma MPE | Train: 5,041 annotations (2,573 adk; 2,468 ms) \| Validation: 654 annotations (345 adk; 309 ms) \| Test: 695 annotations (342 adk; 353 ms) \| External: Qualitative external Kaggle images | Confidence/mAP thresholds; examples shown at 50% confidence | Internal 80/10/10 split plus qualitative external validation | Code: No \| Data: Available from the corresponding author on reasonable request | ^7^ |
|  | YOLOv11_ADK_MS | Object detection/instance segmentation: YOLOv11 instance segmentation model | Cell annotation/cytology image | Manual annotations of adenocarcinoma and mesothelial cells \| Cytopathology diagnosis supported by immunohistochemistry for lung adenocarcinoma MPE | Train: 5,041 annotations (2,573 adk; 2,468 ms) \| Validation: 654 annotations (345 adk; 309 ms) \| Test: 695 annotations (342 adk; 353 ms) \| External: Qualitative external Kaggle images | Confidence/mAP thresholds; examples shown at 50% confidence | Internal 80/10/10 split plus qualitative external validation | Code: No \| Data: Available from the corresponding author on reasonable request |  |
| Gong et al., 2025 | ResNet18_CBAM_ROSE | CNN: ResNet-18 enhanced with convolutional block attention modules (CBAM) | Static high-magnification cytology image | Two senior cytologists selected consensus images; chief physician arbitrated disagreements \| Biopsy/cell block and immunohistochemistry for malignant cases; biopsy/clinical/imaging data for normal cases | Train: Not reported separately \| Validation: Not reported \| Test: 116 images \| External: 0 | Six-way single-choice class output | Internal test set with human-AI comparison | Code: Yes \| Data: All data in the article; code in a public GitHub repository | ^8^ |
| Gonzalez et al., 2020 | InceptionV3_DiffQuik_LCNEC | CNN: Google Inception V3 classifier trained on Diff-Quik smear tiles | Tile and slide | Manual tumor annotations in QuPath \| Original cytology diagnosis with IHC confirmation of neuroendocrine differentiation | Train: 20 training cases; Diff-Quik training tiles 259,838 \| Validation: ROC-derived threshold \| Test: Stain-specific test slides/cases: 1 LCNEC and 8 SCLC for extractable 2x2 \| External: 0 | Slide LCNEC if >95% of patches classified as LCNEC | Internal randomized train/test split | Code: No \| Data: Available from the corresponding author upon reasonable request | ^9^ |
|  | InceptionV3_Pap_LCNEC | CNN: Google Inception V3 classifier trained on Papanicolaou smear tiles | Tile and slide | Manual tumor annotations in QuPath \| Original cytology diagnosis with IHC confirmation of neuroendocrine differentiation | Train: 20 training cases; Pap training tiles 59,072 \| Validation: ROC-derived threshold \| Test: Stain-specific test slides/cases: 1 LCNEC and 7 SCLC for extractable 2x2 \| External: 0 | Slide LCNEC if >75% of patches are classified as LCNEC | Internal randomized train/test split | Code: No \| Data: Available from the corresponding author upon reasonable request |  |
|  | InceptionV3_HE_LCNEC | CNN: Google Inception V3 classifier trained on H&E cell block/core biopsy tiles | Tile and slide | Manual tumor annotations in QuPath \| Original cytology diagnosis with IHC confirmation of neuroendocrine differentiation | Train: 20 training cases; H&E training tiles 145,468 \| Validation: ROC-derived threshold \| Test: Stain-specific test slides/cases: 2 LCNEC and 8 SCLC for extractable 2x2 \| External: 0 | Slide LCNEC if >80% of patches classified as LCNEC | Internal randomized train/test split | Code: No \| Data: Available from the corresponding author upon reasonable request |  |
| Kim et al., 2023 | DenseNet121_BM | CNN: DenseNet121 selected from VGG19, ResNet50, ResNext50, InceptionV3, DenseNet121, and EfficientNetB7 | Patch image | Experienced pathologists/cytotechnologists reviewed extracted patches; low-quality/disagreement patches were excluded \| Corresponding histopathology diagnosis and expert patch review | Train: 27,362 patches from 1,146 WSIs \| Validation: 3,228 patches from 126 WSIs \| Test: 1,272 test patches \| External: 0 | Classification threshold 0.5 | Internal train/validation/test split from multicenter national dataset | Code: No \| Data: AI-Hub dataset source described; study data not fully public in the article | ^10^ |
| Kim et al., 2025 | InceptionResNetV2_patch | CNN: Inception-ResNet-v2 binary patch classifier; selected after comparison with EfficientNet-b0, ResNet50, MobileNet-v2, DenseNet-121, and ResNeXt50 | Patch image | Pathologists classified and labeled split image patches \| Histology-confirmed malignant/benign slide labels with expert patch review | Train: 24,649 patches \| Validation: 3,131 patches \| Test: 3,021 patches \| External: 0 | Sigmoid probability ≥0.5 | Internal 8:1:1 train/validation/test split | Code: No \| Data: Available upon request | ^11^ |
|  | CLAM_SB | Multiple instance learning: ResNet50 feature extractor plus CLAM single-branch attention MIL | Whole-slide image | Slide-level labels from board-certified pathologists and histological confirmation \| Histologically confirmed malignant cases and expert-reviewed benign/malignant WSI labels | Train: 837 WSIs split 8:1:1 \| Validation: Internal validation subset \| Test: 84 WSIs (57 benign/27 malignant) \| External: 93 | Model default/sigmoid probability threshold | Internal train/validation/test split plus external validation archive | Code: No \| Data: Available upon request |  |
|  | CLAM_MB | Multiple instance learning: ResNet50 feature extractor plus CLAM multi-branch attention MIL | Whole-slide image | Slide-level labels from board-certified pathologists and histological confirmation \| Histologically confirmed malignant cases and expert-reviewed benign/malignant WSI labels | Train: 837 WSIs split 8:1:1 \| Validation: Internal validation subset \| Test: 84 WSIs (57 benign/27 malignant) \| External: 93 | Model default/sigmoid probability threshold | Internal train/validation/test split plus external validation archive | Code: No \| Data: Available upon request |  |
| Lai et al., 2025 | VIS_HSI_PCA | Classical machine learning: CCD-to-spectrum reconstruction using CIE 1931 XYZ transformations and PCA score-space feature extraction | Single-cell ROI spectrum | Board-certified cytologist categorized cells as normal, non-normal/reactive, or malignant \| Cytologist-confirmed cell category | Train: No supervised training; 24 cell ROIs used for PCA \| Validation: Not applicable \| Test: Not applicable \| External: 0 | No diagnostic threshold; qualitative PCA score-space separation | Unsupervised proof-of-concept spectral separation | Code: No \| Data: Available upon request | ^12^ |
| Lan et al., 2024 | CUNet3plus_ROSE_Seg | CNN: UNet3+ full-scale connections with Respath, Res2Net bottleneck blocks, DO-Conv kernels, and PFC module | Image patch | Experienced pathologists delineated cancer-cell cluster boundaries using LabelMe \| Pathologist segmentation masks of cancer cell clusters | Train: 91 images → 36,400 patches \| Validation: 26 images → 10,400 patches \| Test: 13 images → 5,200 patches \| External: 20 | Sigmoid segmentation output; threshold not reported | Internal train/validation/test split plus multicenter external validation | Code: No \| Data: No | ^13^ |
| Lemieux et al., 2023 | CyPath_Lung_GLM | Classical machine learning: Stepwise logistic regression/generalized linear model using age and automated flow-cytometry density features | Patient/specimen-level aggregate | Known cancer/non-cancer status from clinical work-up \| Biopsy-confirmed lung cancer; non-cancer status from imaging/clinical evaluation | Train: 168 LSRII samples used for model development/pipeline tuning \| Validation: 150 LSRII validation samples \| Test: 150 LSRII validation; 132 nodule<20 mm subset \| External: 32 | LSRII model-value cutoff 0.28; Navios cutoff 0.5 | Internal validation plus independent validation on a different flow cytometer | Code: No \| Data: Flow files partially available; full data on request | ^14^ |
| Liu et al., 2023 | BALFilter_Reader_YOLOv5x | Object detection CNN: YOLOv5-x enhanced with CIoU loss, image flip, mosaic, HSV augmentation, default NMS, and TTA | Large-field image/image block | Two experienced pathologists annotated tumor/background cells; disagreements resolved by discussion \| Histopathology for clinical cases; pathologist cell annotations for training blocks | Train: 240 image blocks (89 positive/151 negative) \| Validation: 60 image blocks (23 positive/37 negative) \| Test: 24 clinical cases (18 positive/6 negative) \| External: 0 | S0.9 diagnostic indicator with ROC-derived fold thresholds | Clinical threefold cross-validation on 24 cases | Code: Yes \| Data: No | ^15^ |
| Taher et al., 2015 | ANN_Sputum_BM | ANN: Feedforward multilayer perceptron with backpropagation; hidden nodes varied; 10-fold CV | Segmented sputum cell/image | Pathologist diagnosis: extracted feature vectors from segmented cells \| Pathologist-identified benign/malignant cells | Train: 100 sputum images/features with 10-fold cross-validation \| Validation: 10-fold cross-validation \| Test: 10-fold cross-validation \| External: None | Model output/default | Internal 10-fold cross-validation | Code: No \| Data: No | ^16^ |
|  | SVM_Sputum_BM | SVM: Gaussian-kernel SVM; kernel width σ=0.6; feature subset NC ratio, curvature, circularity | Segmented sputum cell/image | Pathologist diagnosis: extracted feature vectors from segmented cells \| Pathologist-identified benign/malignant cells | Train: 100 sputum images/features with 10-fold cross-validation \| Validation: 10-fold cross-validation \| Test: 10-fold cross-validation \| External: None | Gaussian kernel width optimized experimentally | Internal 10-fold cross-validation | Code: No \| Data: No |  |
| Taher et al., 2023 | Bayesian_RuleCAD_Sputum | Bayesian classifier / rule-based CAD: Bayesian histogram pixel classifier, 8-connected component region detection, NC ratio and maximum drawable circle rules | Sputum image/cell region | Manual masks for segmentation; experimented pathologist diagnosis \| Manual segmentation masks and pathologist diagnosis | Train: 65 images for Bayesian training; 100 images for CAD comparison \| Validation: 35 images for segmentation testing \| Test: 100 images for CAD-pathologist comparison \| External: None | λ and rule thresholds optimized experimentally (λ=7; T1=0.75, T2=0.85, T3=0.95, T4=300) | Internal test set and pathologist comparison | Code: No \| Data: No | ^17^ |
| Tanaka et al., 2025 | DenseNet121_LBC_DCNN | DCNN: DenseNet-121 with AdamW optimizer, softmax cross entropy, 5-fold cross-validation | Patch and case-level WSI LBC images | Experienced cytopathologist annotation; final pathology diagnoses checked by two pathologists \| Final surgical pathology and annotated cytology patches | Train: 9,141 patches / 45 cases in 5-fold cross-validation \| Validation: 5-fold cross-validation \| Test: 5-fold cross-validation patch- and case-level folds \| External: None | Softmax/model default; case-level sum of patch log-likelihoods | Internal 5-fold cross-validation | Code: No \| Data: Article states data included in the article and supplementary material | ^18^ |
| Tang et al., 2024 | DualBranch_KF_GCN_CNN | GCN + CNN: GCN spatial branch with cross-attention pooling + CNN texture branch with Daubechies/Gabor filters; fused by MLP/softmax | Digital cytopathology image | Pathology-labeled ADC and SCC cytopathology images \| ADC/SCC class diagnosis | Train: 169 training images augmented threefold to 676 images \| Validation: 5-fold internal cross-validation within training set \| Test: 40-image held-out/external test set \| External: 40-image test set from same retrospective dataset (20 ADC, 20 SCC) | Softmax/model default; no diagnostic threshold reported | Training set 5-fold CV plus 40-image external test set | Code: No \| Data: No | ^19^ |
| Teramoto et al., 2017 | DCNN_3class_LungCytology | DCNN: Three convolution layers, three pooling layers, two fully connected layers, dropout, softmax output | Microscopic cytology image | Final histopathology and immunohistochemistry diagnosis \| Histopathology/IHC-confirmed cancer type | Train: Augmented training images in 3-fold cross-validation; original 298 images \| Validation: Threefold cross-validation \| Test: 298 original images evaluated across folds \| External: None | Softmax maximum probability/class output | Internal threefold cross-validation; images from the same specimen kept in the same fold | Code: No \| Data: No | ^20^ |
| Teramoto et al., 2019 | VGG16_BM_aug | DCNN: ImageNet-pretrained VGG-16; fully connected layers replaced with 1024, 256, and 2 units; softmax output | Patch and case aggregate | Cytopathologist-confirmed patch labels and case reference diagnosis \| Histopathology/IHC-confirmed benign/malignant diagnosis | Train: 621 original patches; 60,000 augmented patches \| Validation: NR \| Test: 3-fold CV; 46 cases \| External: NR | Model default; ROC analyses also reported | Internal threefold cross-validation | Code: No \| Data: Supplementary data DOI only | ^21^ |
| Teramoto et al., 2020 | VGG16_PGGAN_BM | DCNN + GAN: PGGAN-generated benign/malignant cytology images for pretraining; VGG-16 fine-tuned on actual patches | Patch | Two cytopathologists evaluated malignant-cell presence in patches \| Histopathology/IHC-confirmed benign/malignant diagnosis and cytopathologist patch labels | Train: 793 actual patches plus augmented/synthesized images \| Validation: 173 \| Test: 620 images in reported 3-fold DTA table \| External: NR | Cutoff 0.5 for malignancy output | Internal threefold cross-validation with validation images | Code: No \| Data: Figshare dataset URL reported in paper | ^22^ |
| Teramoto et al., 2022 | AlexNet_3class | DCNN: ImageNet-pretrained AlexNet with replacement fully connected layers 1024, 256, 3 | Image | Final histopathological and immunohistochemical diagnosis \| Histopathology/IHC-confirmed subtype | Train: 289 original images; augmented to 5,000/class/fold \| Validation: NR \| Test: 3-fold CV \| External: NR | Model default softmax | Internal threefold cross-validation | Code: No \| Data: No | ^23^ |
|  | InceptionV3_3class | DCNN: ImageNet-pretrained Inception V3 with replacement fully connected layers 1024, 256, 3 | Image | Final histopathological and immunohistochemical diagnosis \| Histopathology/IHC-confirmed subtype | Train: 289 original images; augmented to 5,000/class/fold \| Validation: NR \| Test: 3-fold CV \| External: NR | Model default softmax | Internal threefold cross-validation | Code: No \| Data: No |  |
|  | VGG16_3class | DCNN: ImageNet-pretrained VGG16 with replacement fully connected layers 1024, 256, 3 | Image | Final histopathological and immunohistochemical diagnosis \| Histopathology/IHC-confirmed subtype | Train: 289 original images; augmented to 5,000/class/fold \| Validation: NR \| Test: 3-fold CV \| External: NR | Model default softmax | Internal threefold cross-validation | Code: No \| Data: No |  |
|  | ResNet50_3class | DCNN: ImageNet-pretrained ResNet50 with replacement fully connected layers 1024, 256, 3 | Image | Final histopathological and immunohistochemical diagnosis \| Histopathology/IHC-confirmed subtype | Train: 289 original images; augmented to 5,000/class/fold \| Validation: NR \| Test: 3-fold CV \| External: NR | Model default softmax | Internal threefold cross-validation | Code: No \| Data: No |  |
|  | NB_DCNN_probs_3class | Classical machine learning + DCNN features: Naïve Bayes classifier applied to 12 probability features from AlexNet/InceptionV3/VGG16/ResNet50 | Image | Final histopathological and immunohistochemical diagnosis \| Histopathology/IHC-confirmed subtype | Train: Outputs from four fine-tuned DCNNs \| Validation: NR \| Test: 3-fold CV \| External: NR | Classifier default | Internal threefold cross-validation | Code: No \| Data: No |  |
|  | SVM_DCNN_probs_3class | Classical machine learning + DCNN features: Polynomial-kernel SVM applied to 12 probability features from four DCNNs | Image | Final histopathological and immunohistochemical diagnosis \| Histopathology/IHC-confirmed subtype | Train: Outputs from four fine-tuned DCNNs \| Validation: NR \| Test: 3-fold CV \| External: NR | Model/default SVM parameters in paper Table 3 | Internal threefold cross-validation | Code: No \| Data: No |  |
|  | RF_DCNN_probs_3class | Classical machine learning + DCNN features: Random forest with 20 trees applied to 12 probability features from four DCNNs | Image | Final histopathological and immunohistochemical diagnosis \| Histopathology/IHC-confirmed subtype | Train: Outputs from four fine-tuned DCNNs \| Validation: NR \| Test: 3-fold CV \| External: NR | Model/default RF parameters in paper Table 3 | Internal threefold cross-validation | Code: No \| Data: No |  |
|  | NN_DCNN_probs_3class | Neural network + DCNN features: Two-hidden-layer neural network (100-100, tanh, SGD) applied to 12 probability features from four DCNNs | Image | Final histopathological and immunohistochemical diagnosis \| Histopathology/IHC-confirmed subtype | Train: Outputs from four fine-tuned DCNNs \| Validation: NR \| Test: 3-fold CV \| External: NR | Model/default NN parameters in paper Table 3 | Internal threefold cross-validation | Code: No \| Data: No |  |
| Tondo et al., 2025 | AIROSE_InceptionV3_NN | Classical machine learning + CNN embeddings: Orange image embedding with Google Inception v3 (2048 features), FCBF feature selection, neural network classifier | Static ROSE micrograph | Remote pathologist and final histology; training set refined by pathologist \| Definitive histological report, possibly with immunohistochemistry | Train: 252 positive/negative images; 277 including doubtful \| Validation: NR \| Test: 10-fold cross-validation \| External: NR | Model default in Orange; best model selected by CA/F1/MCC | Internal 10-fold cross-validation | Code: No \| Data: Data available on request | ^24^ |
| Wang et al., 2025a | YOLOv8_SE_SIC_BM | Deep object detection/segmentation: YOLOv8x/YOLOv8-seg with Darknet backbone plus Squeeze-and-Excitation attention | Image with pixel-level malignant-region output | Two pathologists annotated/diagnosed; adjudicating pathologist for disagreements \| Postoperative FFPE histopathology gold standard | Train: 3,689 images \| Validation: 461 images \| Test: 460 internal test images plus 311 SIC additional test images \| External: Pleural fluid 120 and BAL 120 additional generalization sets | Image is malignant if ≥1 positive instance | Internal 8:1:1 split plus additional test sets | Code: Yes \| Data: Data on request; code available at GitHub URL in paper | ^25^ |
| Wang et al., 2025b | SERS_CEA_LDA | Linear discriminant analysis: PCA-selected SERS spectral PCs fused with digitized serum CEA, then LDA | Pleural-effusion SERS spectrum plus serum CEA indicator | Clinical group labels from histopathologic/cytologic diagnosis and medical records \| Patient clinical group label applied to spectra | Train: Five-fold CV; spectra-level training folds not separately reported \| Validation: NR \| Test: Pairwise test folds across 212 spectra \| External: None | LDA posterior probability threshold 0.5 | Internal five-fold cross-validation | Code: No \| Data: Data available from authors on reasonable request | ^26^ |
|  | SERS_CEA_SVM | Support vector machine: PCA-selected SERS spectral PCs fused with digitized serum CEA, then SVM | Pleural-effusion SERS spectrum plus serum CEA indicator | Clinical group labels from histopathologic/cytologic diagnosis and medical records \| Patient clinical group label applied to spectra | Train: Five-fold CV; spectra-level training folds not separately reported \| Validation: NR \| Test: Pairwise test folds across 212 spectra \| External: None | Model-default SVM decision threshold | Internal five-fold cross-validation | Code: No \| Data: Data available from authors on reasonable request |  |
|  | SERS_CEA_KNN | k-nearest neighbor: PCA-selected SERS spectral PCs fused with digitized serum CEA, then KNN | Pleural-effusion SERS spectrum plus serum CEA indicator | Clinical group labels from histopathologic/cytologic diagnosis and medical records \| Patient clinical group label applied to spectra | Train: Five-fold CV; spectra-level training folds not separately reported \| Validation: NR \| Test: Pairwise test folds across 212 spectra \| External: None | Model-default KNN class vote | Internal five-fold cross-validation | Code: No \| Data: Data available from authors on reasonable request |  |
| Wilbur et al., 2015 | LuCED_CellCT_BM | Automated 3D morphometric classifier: Algorithmic classifiers using 594 morphology-based features per epithelial cell | Sputum epithelial cell/specimen | Known pathology/clinical outcomes; classifier-flagged cells underwent cytotechnologist/pathologist hierarchical review \| Patient/specimen diagnosis | Train: Not reported; classifiers trained previously \| Validation: NR \| Test: 91 \| External: NR | Specimen is called abnormal if any abnormal cell is detected/confirmed | Known-outcome clinical feasibility test set | Code: No \| Data: No | ^27^ |
| Xie et al., 2022 | Aitrox_ResNet18_WSI_BM | Weakly supervised DCNN / multiple-instance learning: ResNet18 with MIL; WSI malignant if >10 malignant patches | Pleural effusion cytology WSI / patch | WSI labels from pathology reports; slides checked by senior cytopathologist \| Patient/WSI diagnosis | Train: 266 \| Validation: 78 \| Test: 60 \| External: NR | Patch probability >0.50; WSI malignant if >10 malignant patches | Internal random train/validation/test split | Code: No \| Data: Available from the corresponding author on reasonable request | ^28^ |
| Yan et al., 2024 | ROSE_AI_ResNet101_BM | Deep convolutional neural network: ResNet101 implemented in PyTorch | ROSE cytology image; the patient is positive if any image is positive | Experienced cytopathologist labels accepted only when consistent with the final pathological diagnosis \| Image label and patient final diagnosis | Train: 4106 \| Validation: 544 \| Test: 526 internal-test images / 337 internal-test patients \| External: 1181 temporal-test images / 145 patients | Image confidence threshold 0.5; patient-level cancer threshold 0.55 with at least one malignant image | Internal random image split plus temporal same-center test group | Code: No \| Data: Raw data available from authors upon request | ^29^ |
| Zhang et al., 2026 | CNN_LSTM_Raman_BM | Deep neural network / Raman spectroscopy: Dual-stream CNN-LSTM with two convolutional modules, one LSTM module, feature fusion, and a fully connected classifier | Raman spectrum from cytological smear | Histopathologically confirmed lung cancer vs benign/non-cancer diagnosis \| Patient diagnosis applied to spectra | Train: 760 \| Validation: 5-fold CV on training spectra \| Test: 190 \| External: NR | Model-default binary classifier threshold | Stratified 8:2 spectrum-level split plus five-fold cross-validation on training set | Code: No \| Data: Data confidential | ^30^ |
| Zhao et al., 2025a | RIOM_ROSE_ImageOnly | CNN / deep learning: ResNet-50 pretrained on ImageNet with spatial attention after layer4; global average pooling; fully connected classifier 2048→1024→512→5 | ROSE cytology ROI image | Board-certified pathologists annotated 995 ROIs; final histopathologic diagnosis used for class labels \| Final pathology-confirmed benign or malignant subtype | Train: 70% of 995 ROIs \| Validation: 15% of 995 ROIs \| Test: 15% of 995 ROIs / 155 evaluable cases in reported matrix \| External: 0 | Model-default five-class argmax output; best model selected by validation accuracy | Internal random ROI/case split; no external validation | Code: No \| Data: No | ^31^ |
|  | RSMM_ROSE_Serum | Multimodal CNN / cross-attention deep learning: ResNet-50 image branch plus serum marker MLP (5→32→256) fused by multi-head cross-attention; fusion classifier 256→512→5 | ROSE cytology ROI image plus continuous serum SCCA, ProGRP, CEA, NSE, and CYFRA21-1 | Board-certified pathologists annotated ROIs; serum biomarkers from clinical assays; final histopathologic diagnosis for class labels \| Final pathology-confirmed benign or malignant subtype | Train: 70% of 995 ROIs \| Validation: 15% of 995 ROIs \| Test: 15% of 995 ROIs / 155 evaluable cases in reported matrix \| External: 0 | Model-default five-class argmax output; best model selected by validation accuracy | Internal random ROI/case split; no external validation | Code: No \| Data: No |  |
| Zhao et al., 2025b | LESSEL_WSI_BM | Deep learning pipeline / CNN object detection and classification: YOLOX single-cell extraction, EfficientNet-B2 QC, U-Net single-cell segmentation, dual EfficientNet-B4 malignant/benign classifiers, aggregated by WSI-level ETC% threshold | Pap-stained BAL cytology WSI / extracted single cells | scDNA-Seq-confirmed ETCs for malignant cell labels; benign cells selected from benign BALF; specimen labels from histopathology/clinical follow-up \| Single-cell scDNA-Seq for cell labels; patient/specimen clinical diagnosis for WSI DTA | Train: 580 ETCs + 1106 benign cells split 7:2:1; WSI cut-off derived in 128-specimen discovery cohort \| Validation: 128 same-center validation specimens after exclusions \| Test: Internal single-cell test set plus same-center clinical validation \| External: 118 external validation specimens after exclusions | LESSEL-predicted ETC% cutoff 0.2% derived in discovery cohort | Discovery cohort plus same-center validation and independent external validation | Code: No \| Data: Yes; GSA HRA008613 | ^32^ |

CNN, convolutional neural network; GBRT, gradient boosted regression tree; HRNet, high-resolution network; NIRS, near-infrared spectroscopy; PLS, partial least squares; RF, random forest; SVM, support vector machine; RFE, recursive feature elimination; GBM, gradient boosting machine; MIL, multiple instance learning; HMLCS, hierarchical multi-label classification system; PCA, principal component analysis; LDA, linear discriminant analysis; ULDA, uncorrelated linear discriminant analysis; PLSDA, partial least squares discriminant analysis; ADK, adenocarcinoma cell; MS, mesothelial cell; YOLO, you only look once; mAP, mean average precision; CBAM, convolutional block attention module; ROC, receiver operating characteristic; VGG, visual geometry group; ResNet, residual network; ResNext, residual next; MobileNet, mobile network; CLAM, clustering-constrained attention multiple instance learning; SB, single-branch; MB, multi-branch; VIS, visible; HSI, hyperspectral imaging; CCD, charge-coupled device; CIE, International Commission on Illumination; UNet, u-shaped network; Respath, residual path; DO-Conv, depthwise over-parameterized convolutional layer; PFC, parallel feature compression; GLM, generalized linear model; CIoU, complete intersection over union; HSV, hue, saturation, value; NMS, non-maximum suppression; TTA, test-time augmentation; ANN, artificial neural network; CAD, computer-aided diagnosis; NC ratio, nuclear-to-cytoplasmic ratio; DCNN, deep convolutional neural network (already listed but used here as a model name); AdamW, Adam with weight decay; GCN, graph convolutional network; KF, Kalman filter; MLP, multilayer perceptron; PGGAN, progressive growing generative adversarial network; NB, naïve Bayes; NN, neural network; FCBF, fast correlation-based filter; CA, classification accuracy; MCC, Matthews correlation coefficient; SE, squeeze-and-excitation; FFPE, formalin-fixed paraffin-embedded; KNN, k-nearest neighbor; 3D, three-dimensional; Aitrox, AI for Thorax (proprietary model name); LSTM, long short-term memory; RIOM, ROI image-only model; RSMM, ROI serum multimodal model; SCCA, squamous cell carcinoma antigen; ProGRP, progastrin-releasing peptide; NSE, neuron-specific enolase; CYFRA21-1, cytokeratin 19 fragment; LESSEL, label-efficient single-cell expression learning; ETC%, epithelial tumor cell percentage; GSA, Genome Sequence Archive

## Supplementary Table S6. Risk of bias and applicability assessment using QUADAS-2 with AI-specific signaling items.

| **Study ID** | **Patient selection RoB/applicability** | **Index test RoB/applicability** | **Reference standard RoB/applicability** | **Flow and timing RoB** | **AI data leakage concern** | **External validation** | **Overall RoB** | **Overall applicability** | **Reference** |
| --- | --- | --- | --- | --- | --- | --- | --- | --- | --- |
| Ai et al., 2022 | High / Low concern | High / Low concern | Low / Low concern | Unclear | Unclear | No | High | Low concern | ^1^ |
| Chen et al., 2020 | High / High concern | High / High concern | Unclear / Unclear | High | High | No | High | High concern | ^2^ |
| Chen et al., 2021a | Unclear / Low concern | High / Low concern | Low / Low concern | Unclear | Low | No | High | Low concern | ^3^ |
| Chen et al., 2021b | High / High concern | Unclear / High concern | Low / High concern | Unclear | Unclear | No | High | High concern | ^4^ |
| Chen et al., 2024 | High / Low concern | High / Low concern | Low / Low concern | Unclear | Unclear | No | High | Low concern | ^5^ |
| Ganesh et al., 2022 | High / High concern | High / High concern | Unclear / High concern | Unclear | Unclear | No | High | High concern | ^6^ |
| Giarnieri et al., 2025 | High / Low concern | High / Low concern | Low / Low concern | Unclear | Unclear | Qualitative only | High | Low concern | ^7^ |
| Gong et al., 2025 | High / Low concern | High / Low concern | Low / Low concern | Unclear | Unclear | No | High | Low concern | ^8^ |
| Gonzalez et al., 2020 | High / Low concern | High / Low concern | Unclear / Low concern | Unclear | Unclear | No | High | Low concern | ^9^ |
| Kim et al., 2023 | Unclear / Low concern | High / Low concern | Low / Low concern | Unclear | Unclear | No | High | Low concern | ^10^ |
| Kim et al., 2025 | Unclear / Low concern | High / Low concern | Low / Low concern | Unclear | Unclear | Yes | High | Low concern | ^11^ |
| Lai et al., 2025 | High / High concern | High / High concern | Unclear / Low concern | Unclear | Unclear | No | High | High concern | ^12^ |
| Lan et al., 2024 | High / Low concern | High / Low concern | Low / Low concern | Unclear | Unclear | Yes | High | Low concern | ^13^ |
| Lemieux et al., 2023 | High / High concern | High / High concern | Low / Low concern | Unclear | Unclear | Yes | High | High concern | ^14^ |
| Liu et al., 2023 | High / Low concern | High / Low concern | Low / Low concern | Unclear | Unclear | No | High | Low concern | ^15^ |
| Taher et al., 2015 | High / Low concern | High / Low concern | Low / Low concern | Unclear | Unclear | No | High | Low concern | ^16^ |
| Taher et al., 2023 | High / Low concern | High / Low concern | Unclear / Low concern | Unclear | Unclear | No | High | Low concern | ^17^ |
| Tanaka et al., 2025 | High / Low concern | High / Low concern | Low / Low concern | Unclear | Low | No | High | Low concern | ^18^ |
| Tang et al., 2024 | High / Low concern | High / Low concern | Unclear / Low concern | Unclear | Unclear | Held-out test set only | High | Low concern | ^19^ |
| Teramoto et al., 2017 | High / Low concern | High / Low concern | Low / Low concern | Unclear | Low | No | High | Low concern | ^20^ |
| Teramoto et al., 2019 | High / Low concern | High / Low concern | Low / Low concern | Unclear | High | No | High | Low concern | ^21^ |
| Teramoto et al., 2020 | High / Low concern | High / Low concern | Low / Low concern | Unclear | High | No | High | Low concern | ^22^ |
| Teramoto et al., 2022 | High / Low concern | High / Low concern | Low / Low concern | Unclear | High | No | High | Low concern | ^23^ |
| Tondo et al., 2025 | High / Low concern | High / Low concern | Low / Low concern | Unclear | High | No | High | Low concern | ^24^ |
| Wang et al., 2025a | High / Unclear | High / Low concern | Low / Low concern | Unclear | High | No | High | Unclear | ^25^ |
| Wang et al., 2025b | High / High concern | High / High concern | Low / Low concern | Unclear | High | No | High | High concern | ^26^ |
| Wilbur et al., 2015 | High / Low concern | High / Low concern | Unclear / Low concern | Unclear | Unclear | No | High | Low concern | ^27^ |
| Xie et al., 2022 | High / Low concern | High / Low concern | Low / Low concern | Unclear | Unclear | No | High | Low concern | ^28^ |
| Yan et al., 2024 | High / Low concern | High / Low concern | Low / Low concern | Unclear | Unclear | Temporal same-center only | High | Low concern | ^29^ |
| Zhang et al., 2026 | High / High concern | High / High concern | Low / Low concern | Unclear | High | No | High | High concern | ^30^ |
| Zhao et al., 2025a | High / Low concern | High / Low concern | Low / Low concern | Unclear | Unclear | No | High | Low concern | ^31^ |
| Zhao et al., 2025b | High / Low concern | Low / Low concern | Low / Low concern | Low | Low | Yes | High | Low concern | ^32^ |

## Supplementary Table S7. Benign-malignant diagnostic test accuracy data.

| **Study ID** | **Model ID** | **Dataset ID** | **Specimen/population** | **Threshold** | **Reference standard** | **Unit** | **TP** | **FP** | **FN** | **TN** | **Sensitivity** | **Specificity** | **Include?** | **Reference** |
| --- | --- | --- | --- | --- | --- | --- | --- | --- | --- | --- | --- | --- | --- | --- |
| Ai et al., 2022 | AI_ROSE_DenseNet169 | Testing_group | Bronchoscopy ROSE slides (TBNA/TBLB/TBB) | Patch probability ≥0.81 and >6 malignant patches per WSI | Comprehensive clinical diagnosis with pathology/clinical data | Patient/slide | 84 | 4 | 21 | 53 | 0.800 | 0.930 | Yes | ^1^ |
| Chen et al., 2020 | EfficientNet_GBRT_CNN | Not_reported | Pleural fluid/FNAB cytology images | Not reported | Cytology label; details not reported | Image | NR | NR | NR | NR | NR | NR | No | ^2^ |
| Chen et al., 2021a | ResNet101_BM | Test_patches | EBUS ROSE cytology images | Patch classifier output | Formal cytopathologic results | Patch | 563 | 5 | 7 | 415 | 0.988 | 0.988 | No | ^3^ |
|  | ResNet101_BM | Test_images | EBUS ROSE cytology images | Softmax output >0.99 for at least one sliding-window patch | Formal cytopathologic results | Image/micrograph | 56 | 2 | 1 | 7 | 0.982 | 0.778 | No |  |
|  | ResNet101_BM | Test_patients | EBUS ROSE cytology images | Image-level result by Softmax >0.99; patient-level majority vote | Formal cytopathologic results | Patient | 11 | 1 | 0 | 2 | 1.000 | 0.667 | Yes |  |
| Chen et al., 2021b | NIRS_PLS | Test_set | Pleural effusion supernatant | Model default | MPE cytology/histology; BPE TB culture/clinical diagnosis | Specimen | 10 | 3 | 1 | 6 | 0.909 | 0.667 | No | ^4^ |
|  | NIRS_RF | Test_set | Pleural effusion supernatant | Model default | MPE cytology/histology; BPE TB culture/clinical diagnosis | Specimen | 11 | 1 | 0 | 8 | 1.000 | 0.889 | No |  |
|  | NIRS_SVM | Test_set | Pleural effusion supernatant | Linear SVM decision boundary; cost=5 | MPE cytology/histology; BPE TB culture/clinical diagnosis | Specimen | 11 | 0 | 0 | 9 | 1.000 | 1.000 | Yes |  |
|  | NIRS_GBM | Test_set | Pleural effusion supernatant | Model default | MPE cytology/histology; BPE TB culture/clinical diagnosis | Specimen | 10 | 0 | 1 | 9 | 0.909 | 1.000 | No |  |
| Chen et al., 2024 | HMLCS_ROSE_Serum | Test_set_BM_module | Transbronchial ROSE WSIs | Model output/default threshold | Final transbronchial sampling diagnosis with immunohistochemistry/follow-up | WSI/slide | 132 | 5 | 14 | 23 | 0.904 | 0.821 | Yes | ^5^ |
| Ganesh et al., 2022 | SERS_PLSDA_Lung_vs_Control | CV_lung_vs_control | Pleural fluid supernatant | PLS-DA class assignment | Clinical diagnosis of lung cancer vs non-cancer control | SERS spectrum; subject-level counts not reported | NR | NR | NR | NR | NR | NR | No | ^6^ |
|  | SERS_PLSDA_Lung_vs_Control | CV_all_cancer_vs_control | Pleural fluid supernatant | PLS-DA class assignment | Clinical diagnosis of cancer vs non-cancer control | SERS spectrum; subject-level counts not reported | NR | NR | NR | NR | NR | NR | No |  |
| Giarnieri et al., 2025 | YOLOv8_ADK_MS | Test_annotations | Pleural effusion cytology images | Object-detection confidence / mAP threshold | Cytopathology diagnosis with IHC confirmation/rule-out of lung adenocarcinoma MPE | Cell annotation/detection | NR | NR | NR | NR | NR | NR | No | ^7^ |
|  | YOLOv11_ADK_MS | Test_annotations | Pleural effusion cytology images | Object-detection confidence / mAP threshold | Cytopathology diagnosis with IHC confirmation/rule-out of lung adenocarcinoma MPE | Cell annotation/detection | NR | NR | NR | NR | NR | NR | No |  |
| Gong et al., 2025 | ResNet18_CBAM_ROSE | Test_116_images | Respiratory cytology images | Single-choice class output | Biopsy/cell block/IHC confirmation and clinical/imaging normal adjudication | Image | NR | NR | NR | NR | NR | NR | No | ^8^ |
| Kim et al., 2023 | DenseNet121_BM | Test_patches_AI | Sputum and bronchial washing respiratory cytology | Classification threshold 0.5 | Expert patch labels based on corresponding histopathology/cytology and review | Patch | 686 | 10 | 29 | 547 | 0.959 | 0.982 | Yes | ^10^ |
|  | DenseNet121_BM | Validation_HE | Respiratory cytology image patches | Classification threshold 0.5 | Expert patch labels based on corresponding histopathology/cytology and review | Patch | 32 | 4 | 4 | 529 | 0.889 | 0.992 | No |  |
|  | DenseNet121_BM | Validation_PAP | Respiratory cytology image patches | Classification threshold 0.5 | Expert patch labels based on corresponding histopathology/cytology and review | Patch | 1371 | 73 | 61 | 1227 | 0.957 | 0.944 | No |  |
| Kim et al., 2025 | InceptionResNetV2_patch | Test_patches | Pleural fluid cytology WSI patches | Sigmoid probability ≥0.5 | Histology-confirmed malignant cases and expert-reviewed cytology patch labels | Patch | 1523 | 20 | 6 | 1472 | 0.996 | 0.987 | No | ^11^ |
|  | CLAM_SB | Test_WSIs | Pleural fluid cytology WSIs | Model output/default threshold | Histologically confirmed malignant cases and board-certified pathologist review | WSI/slide | 21 | 10 | 6 | 47 | 0.778 | 0.825 | No |  |
|  | CLAM_MB | Test_WSIs | Pleural fluid cytology WSIs | Model output/default threshold | Histologically confirmed malignant cases and board-certified pathologist review | WSI/slide | 22 | 3 | 5 | 54 | 0.815 | 0.947 | Yes |  |
|  | CLAM_MB | External_QAP_WSIs | Pleural fluid cytology WSIs | Model output/default threshold | Histologically confirmed malignant cases and board-certified pathologist review | WSI/slide | 55 | 3 | 6 | 29 | 0.902 | 0.906 | Check |  |
| Lai et al., 2025 | VIS_HSI_PCA | Twenty_four_cell_ROIs | Pleural effusion cytology cells | No diagnostic threshold; PCA score-space visualization | Board-certified cytologist cell classification | Cell/ROI | NR | NR | NR | NR | NR | NR | No | ^12^ |
| Lan et al., 2024 | CUNet3plus_ROSE_Seg | Internal_external_segmentation | ROSE cytopathology images from lung cancer cases | Sigmoid segmentation output; threshold NR | Pathologist segmentation masks and labels | Pixel/image patch | NR | NR | NR | NR | NR | NR | No | ^13^ |
| Lemieux et al., 2023 | CyPath_Lung_GLM | LSRII_validation | Induced sputum | Model value >0.28 | Biopsy-confirmed cancer; imaging/clinical non-cancer classification | Participant/sputum sample | 23 | 15 | 5 | 107 | 0.821 | 0.877 | Yes | ^14^ |
|  | CyPath_Lung_GLM | LSRII_nodules_lt20mm | Induced sputum | Model value >0.28 | Biopsy-confirmed cancer; imaging/clinical non-cancer classification | Participant/sputum sample | 12 | 15 | 1 | 104 | 0.923 | 0.874 | No |  |
|  | CyPath_Lung_GLM | Navios_external | Induced sputum | Model value >0.5 | Biopsy-confirmed cancer; imaging/clinical non-cancer classification | Participant/sputum sample | 5 | 6 | 1 | 20 | 0.833 | 0.769 | Yes |  |
| Liu et al., 2023 | BALFilter_Reader_YOLOv5x | Clinical_cross_validation_24_cases | BALF cells on PERFECT/BALFilter filters | S0.9 indicator with fold-specific ROC thresholds | Histopathology results as the gold standard | Patient/case | 12 | 0 | 6 | 6 | 0.667 | 1.000 | Yes | ^15^ |
| Taher et al., 2015 | ANN_Sputum_BM | Tenfold_CV_100_images | Sputum cytology images | ANN classifier output | Pathologist-identified cells/diagnosis | Image/cell | NR | NR | NR | NR | NR | NR | No | ^16^ |
|  | SVM_Sputum_BM | Tenfold_CV_100_images | Sputum cytology images | SVM Gaussian-kernel classifier output | Pathologist-identified cells/diagnosis | Image/cell | NR | NR | NR | NR | NR | NR | No |  |
| Taher et al., 2023 | Bayesian_RuleCAD_Sputum | CAD_100_images | Sputum cytology images | NC ratio/MDC diagnostic rules | Experimented pathologist diagnosis | Image/cell | 65 | 10 | 8 | 17 | 0.890 | 0.630 | Check | ^17^ |
| Tanaka et al., 2025 | DenseNet121_LBC_DCNN | Patch_level_5fold_CV_BM | Scraped surgical LBC WSI patches | Softmax/model default | Final surgical pathology and cytopathologist patch annotation | Patch | 6227 | 405 | 177 | 2332 | 0.972 | 0.852 | No | ^18^ |
|  | DenseNet121_LBC_DCNN | Case_level_5fold_CV_BM | Scraped surgical LBC cases/slides | Case-level sum of patch log-likelihoods | Final surgical pathology diagnosis | Case/slide | 34 | 1 | 3 | 7 | 0.919 | 0.875 | Yes |  |
| Teramoto et al., 2019 | VGG16_BM_aug | Patch_level_3fold_CV_no_aug | LBC cytology image patches | Softmax/model output | Histopathology/IHC diagnosis with cytopathologist patch review | Patch | 299 | 199 | 16 | 107 | 0.949 | 0.350 | No | ^21^ |
|  | VGG16_BM_aug | Case_level_3fold_CV_no_aug | LBC cytology cases | Maximum patch malignancy probability per case | Histopathology/IHC diagnosis | Case/patient | 26 | 9 | 2 | 9 | 0.929 | 0.500 | No |  |
|  | VGG16_BM_aug | Patch_level_3fold_CV_augmented | LBC cytology image patches | Softmax/model output | Histopathology/IHC diagnosis with cytopathologist patch review | Patch | 298 | 112 | 17 | 194 | 0.946 | 0.634 | No |  |
|  | VGG16_BM_aug | Case_level_3fold_CV_augmented | LBC cytology cases | Maximum patch malignancy probability per case | Histopathology/IHC diagnosis | Case/patient | 25 | 3 | 3 | 15 | 0.893 | 0.833 | Yes |  |
| Teramoto et al., 2020 | VGG16_PGGAN_BM | Patch_level_3fold_CV_PGGAN | LBC cytology image patches | Malignancy output cutoff 0.5 | Histopathology/IHC diagnosis with cytopathologist patch review | Patch | 268 | 45 | 46 | 261 | 0.854 | 0.853 | Yes | ^22^ |
| Tondo et al., 2025 | AIROSE_InceptionV3_NN | Positive_negative_10fold_CV | Static ROSE biopsy sample images | Neural network classification of Inception v3 embeddings | Definitive histological report with possible immunohistochemistry | Slide/image | 188 | 12 | 7 | 45 | 0.964 | 0.789 | Yes | ^24^ |
| Wang et al., 2025a | YOLOv8_SE_SIC_BM | Additional_SIC_image_level | Scratch-imprint cytology images | Image is categorized as malignant if ≥1 positive instance | Postoperative FFPE histopathology gold standard | Image | 216 | 18 | 10 | 67 | 0.956 | 0.788 | Check | ^25^ |
| Wang et al., 2025b | SERS_CEA_LDA | CV_lung_vs_non_cancer | Pleural effusion SERS spectra plus serum CEA | Model default/threshold 0.5 where reported | Histopathologic or cytologic examination confirmed the clinical group | Spectrum | 48 | 20 | 12 | 92 | 0.800 | 0.821 | No | ^26^ |
|  | SERS_CEA_LDA | CV_other_cancer_vs_non_cancer | Pleural effusion SERS spectra plus serum CEA | Model default/threshold 0.5 where reported | Histopathologic or cytologic examination confirmed the clinical group | Spectrum | 25 | 29 | 15 | 83 | 0.625 | 0.741 | No |  |
|  | SERS_CEA_LDA | CV_lung_vs_other_cancers | Pleural effusion SERS spectra plus serum CEA | Model default/threshold 0.5 where reported | Histopathologic or cytologic examination confirmed the clinical group | Spectrum | 42 | 11 | 18 | 29 | 0.700 | 0.725 | No |  |
|  | SERS_CEA_SVM | CV_lung_vs_non_cancer | Pleural effusion SERS spectra plus serum CEA | Model default/threshold 0.5 where reported | Histopathologic or cytologic examination confirmed the clinical group | Spectrum | 52 | 0 | 8 | 112 | 0.867 | 1.000 | No |  |
|  | SERS_CEA_SVM | CV_other_cancer_vs_non_cancer | Pleural effusion SERS spectra plus serum CEA | Model default/threshold 0.5 where reported | Histopathologic or cytologic examination confirmed the clinical group | Spectrum | 16 | 4 | 24 | 108 | 0.400 | 0.964 | No |  |
|  | SERS_CEA_SVM | CV_lung_vs_other_cancers | Pleural effusion SERS spectra plus serum CEA | Model default/threshold 0.5 where reported | Histopathologic or cytologic examination confirmed the clinical group | Spectrum | 56 | 5 | 4 | 35 | 0.933 | 0.875 | No |  |
|  | SERS_CEA_KNN | CV_lung_vs_non_cancer | Pleural effusion SERS spectra plus serum CEA | Model default/threshold 0.5 where reported | Histopathologic or cytologic examination confirmed the clinical group | Spectrum | 52 | 15 | 8 | 97 | 0.867 | 0.866 | No |  |
|  | SERS_CEA_KNN | CV_other_cancer_vs_non_cancer | Pleural effusion SERS spectra plus serum CEA | Model default/threshold 0.5 where reported | Histopathologic or cytologic examination confirmed the clinical group | Spectrum | 40 | 22 | 0 | 90 | 1.000 | 0.804 | No |  |
|  | SERS_CEA_KNN | CV_lung_vs_other_cancers | Pleural effusion SERS spectra plus serum CEA | Model default/threshold 0.5 where reported | Histopathologic or cytologic examination confirmed the clinical group | Spectrum | 58 | 10 | 2 | 30 | 0.967 | 0.750 | No |  |
| Wilbur et al., 2015 | LuCED_CellCT_BM | Clinical_study_91_specimens | Sputum cytology | Any abnormal cell identified after the LuCED classifier and manual hierarchical cytology review | Confirmed lung cancer vs nonmalignant lung pathology/benign tumor/no known pulmonary pathology at last follow-up | Patient/specimen | 45 | 2 | 4 | 40 | 0.918 | 0.952 | Yes | ^27^ |
| Xie et al., 2022 | Aitrox_ResNet18_WSI_BM | Test_60_WSIs | Pleural effusion liquid-based cytology WSI | Patch probability >0.50; WSI malignant if >10 malignant patches | Pathology report / cytological morphology ± matched cell block with IHC | WSI/slide | 21 | 2 | 3 | 34 | 0.875 | 0.944 | Yes | ^28^ |
| Yan et al., 2024 | ROSE_AI_ResNet101_BM | Internal_testing_images | ROSE cytology image from transbronchial biopsy | Classification confidence threshold 0.5 | Experienced cytopathologist annotation accepted only when consistent with final pathological diagnosis | Image | 320 | 18 | 19 | 169 | 0.944 | 0.904 | No | ^29^ |
|  | ROSE_AI_ResNet101_BM | External_testing_images | ROSE cytology image from transbronchial biopsy | Classification confidence threshold 0.5 | Experienced cytopathologist annotation accepted only when consistent with final pathological diagnosis | Image | 786 | 35 | 80 | 280 | 0.908 | 0.889 | No |  |
|  | ROSE_AI_ResNet101_BM | Internal_testing_patients | ROSE cytology from transbronchial biopsy | Patient-level confidence threshold 0.55; cancer if at least one image positive | Final pathological diagnosis | Patient | 192 | 15 | 20 | 110 | 0.906 | 0.880 | No |  |
|  | ROSE_AI_ResNet101_BM | External_testing_patients | ROSE cytology from transbronchial biopsy | Patient-level confidence threshold 0.55; cancer if at least one image positive | Final pathological diagnosis | Patient | 93 | 13 | 5 | 34 | 0.949 | 0.723 | Yes |  |
| Zhang et al., 2026 | CNN_LSTM_Raman_BM | Independent_test_190_spectra | Raman spectra from cytological smears | Model-default binary classifier threshold | Histopathologic confirmation of primary lung cancer vs benign/non-cancer disease | Spectrum | 26 | 7 | 2 | 155 | 0.929 | 0.957 | No | ^30^ |
| Zhao et al., 2025a | RIOM_ROSE_ImageOnly | Test_set_Fig4A_collapsed_BM | ROSE cytology ROI/case images | Five-class model argmax collapsed to malignant vs benign | Final histopathology with clinical/IHC confirmation | Patient/case | 137 | 8 | 1 | 9 | 0.993 | 0.529 | No | ^31^ |
|  | RSMM_ROSE_Serum | Test_set_Fig4B_collapsed_BM | ROSE cytology ROI/case images plus serum biomarkers | Five-class model argmax collapsed to malignant vs benign | Final histopathology with clinical/IHC confirmation | Patient/case | 135 | 4 | 3 | 13 | 0.978 | 0.765 | Yes |  |
| Zhao et al., 2025b | LESSEL_WSI_BM | Discovery_cohort_cutoff_derivation | BAL cytology WSI | LESSEL-predicted ETC% ≥0.2% | Surgical/biopsy histopathology for malignant; negative pathology/cytology plus ≥6-month follow-up and alternative etiology for benign | BALF specimen/patient | 40 | 0 | 49 | 39 | 0.449 | 1.000 | No | ^32^ |
|  | LESSEL_WSI_BM | Same_center_validation | BAL cytology WSI | LESSEL-predicted ETC% ≥0.2% | Surgical/biopsy histopathology for malignant; negative pathology/cytology plus ≥6-month follow-up and alternative etiology for benign | BALF specimen/patient | 40 | 1 | 44 | 43 | 0.476 | 0.977 | No |  |
|  | LESSEL_WSI_BM | External_validation_center2 | BAL cytology WSI | LESSEL-predicted ETC% ≥0.2% | Surgical/biopsy histopathology for malignant; negative pathology/cytology plus ≥6-month follow-up and alternative etiology for benign | BALF specimen/patient | 39 | 4 | 26 | 49 | 0.600 | 0.925 | Yes |  |

TP, true positive; FP, false positive; FN, false negative; TN, true negative; ROI, region of interest; CV, cross-validation; WSI, whole-slide image; LBC, liquid-based cytology; BAL, bronchoalveolar lavage; BALF, bronchoalveolar lavage fluid; ETC, epithelial tumor cell; ETC%, epithelial tumor cell percentage; MPE, malignant pleural effusion; BPE, benign pleural effusion; PLS-DA, partial least squares discriminant analysis; mAP, mean average precision; QC, quality control; QAP, quality assurance program; MDC, maximum drawable circle; PGGAN, progressive growing generative adversarial network; FFPE, formalin-fixed paraffin-embedded; SIC, scratch-imprint cytology; CEA, carcinoembryonic antigen; KNN, k-nearest neighbor; Aitrox, AI for Thorax; LESSEL, label-efficient single-cell expression learning

## Supplementary Table S8. ADC/SCC/SCLC subtype diagnostic test accuracy data using one-vs-rest coding.

| **Study ID** | **Model ID** | **Dataset ID** | **Target class** | **Comparator classes** | **Population** | **Threshold** | **TP** | **FP** | **FN** | **TN** | **Sensitivity** | **Specificity** | **Include?** | **Reference** |
| --- | --- | --- | --- | --- | --- | --- | --- | --- | --- | --- | --- | --- | --- | --- |
| Chen et al., 2024 | HMLCS_ROSE_Serum | Test_set_final_collapsed | ADC/LUAD | SCC/LUSC + SCLC + NSCLC-NOS/other malignancy; predicted benign/NOS/other collapsed as Other | Malignant cases only; true benign row excluded; final six-class predictions collapsed to ADC/SCC/SCLC/Other | Model output/default threshold | 58 | 17 | 9 | 62 | 0.866 | 0.785 | Yes | ^5^ |
|  |  |  | SCC/LUSC | ADC/LUAD + SCLC + NSCLC-NOS/other malignancy; predicted benign/NOS/other collapsed as Other | Malignant cases only; true benign row excluded; final six-class predictions collapsed to ADC/SCC/SCLC/Other | Model output/default threshold | 15 | 5 | 14 | 112 | 0.517 | 0.957 | Yes |  |
|  |  |  | SCLC | ADC/LUAD + SCC/LUSC + NSCLC-NOS/other malignancy; predicted benign/NOS/other collapsed as Other | Malignant cases only; true benign row excluded; final six-class predictions collapsed to ADC/SCC/SCLC/Other | Model output/default threshold | 26 | 6 | 6 | 108 | 0.812 | 0.947 | Yes |  |
|  |  |  | SCLC | NSCLC + other malignancies | Malignant cases passed to M-sub module after B/M step (n=132) | Model output/default threshold | 26 | 6 | 5 | 95 | 0.839 | 0.941 | Check |  |
|  |  |  | ADC/LUAD | SCC/LUSC + NSCLC-NOS | NSCLC cases passed to NSCLC-sub module (n=86) | Model output/default threshold | 58 | 9 | 3 | 16 | 0.951 | 0.640 | Check |  |
|  |  |  | SCC/LUSC | ADC/LUAD + NSCLC-NOS | NSCLC cases passed to NSCLC-sub module (n=86) | Model output/default threshold | 15 | 3 | 8 | 60 | 0.652 | 0.952 | Check |  |
| Gonzalez et al., 2020 | InceptionV3_DiffQuik_LCNEC | Test_DiffQuik | LCNEC | SCLC | High-grade pulmonary neuroendocrine carcinoma; mixed/unclassifiable cases excluded from 2x2 | Slide classified LCNEC if >95% of patches labelled LCNEC | 1 | 1 | 0 | 7 | 1.000 | 0.875 | Yes | ^9^ |
|  | InceptionV3_Pap_LCNEC | Test_Pap | LCNEC | SCLC | High-grade pulmonary neuroendocrine carcinoma; mixed/unclassifiable cases excluded from 2x2 | Slide classified as LCNEC if >75% of patches labeled LCNEC | 1 | 1 | 0 | 6 | 1.000 | 0.857 | Yes |  |
|  | InceptionV3_HE_LCNEC | Test_HE | LCNEC | SCLC | High-grade pulmonary neuroendocrine carcinoma; mixed/unclassifiable cases excluded from 2x2 | Slide classified as LCNEC if >80% of patches labeled LCNEC | 2 | 1 | 0 | 7 | 1.000 | 0.875 | Yes |  |
| Tanaka et al., 2025 | DenseNet121_LBC_DCNN | Patch_level_5fold_CV_ADC_SQCC | ADC | SCC/SQCC | NSCLC malignant LBC patches; ADC vs SQCC only | Softmax/model output | 4345 | 234 | 411 | 1414 | 0.914 | 0.858 | No | ^18^ |
|  | DenseNet121_LBC_DCNN | Patch_level_5fold_CV_ADC_SQCC | SCC/SQCC | ADC | NSCLC malignant LBC patches; ADC vs SQCC only | Softmax/model output | 1414 | 411 | 234 | 4345 | 0.858 | 0.914 | No |  |
|  | DenseNet121_LBC_DCNN | Case_level_5fold_CV_ADC_SQCC | ADC | SCC/SQCC | NSCLC malignant LBC cases; ADC vs SQCC only | Case-level sum of patch log-likelihoods | 18 | 4 | 4 | 11 | 0.818 | 0.733 | Yes |  |
|  | DenseNet121_LBC_DCNN | Case_level_5fold_CV_ADC_SQCC | SCC/SQCC | ADC | NSCLC malignant LBC cases; ADC vs SQCC only | Case-level sum of patch log-likelihoods | 11 | 4 | 4 | 18 | 0.733 | 0.818 | Yes |  |
| Tang et al., 2024 | DualBranch_KF_GCN_CNN | External_test_40_images | ADC/SCC binary task | Opposite binary class | ADC vs SCC cytopathology images | Softmax/model output | NR | NR | NR | NR | NR | NR | No | ^19^ |
| Teramoto et al., 2017 | DCNN_3class_LungCytology | Threefold_CV_original_images | ADC | SCC/SQCC + SCLC | Three lung cancer types: ADC, SCC, SCLC | Softmax maximum probability | 73 | 42 | 9 | 174 | 0.890 | 0.806 | Yes | ^20^ |
|  | DCNN_3class_LungCytology | Threefold_CV_original_images | SCC/SQCC | ADC + SCLC | Three lung cancer types: ADC, SCC, SCLC | Softmax maximum probability | 75 | 28 | 50 | 145 | 0.600 | 0.838 | Yes |  |
|  | DCNN_3class_LungCytology | Threefold_CV_original_images | SCLC | ADC + SCC/SQCC | Three lung cancer types: ADC, SCC, SCLC | Softmax maximum probability | 64 | 16 | 27 | 191 | 0.703 | 0.923 | Yes |  |
| Teramoto et al., 2022 | AlexNet_3class | Threefold_CV_original_images | ADC | SCC/SQCC + SCLC | Three lung cancer types: ADC, SCC/SQCC, SCLC | Softmax/model output or downstream classifier class prediction | 57 | 38 | 18 | 176 | 0.760 | 0.822 | No | ^23^ |
|  | AlexNet_3class | Threefold_CV_original_images | SCC/SQCC | ADC + SCLC | Three lung cancer types: ADC, SCC/SQCC, SCLC | Softmax/model output or downstream classifier class prediction | 75 | 12 | 55 | 147 | 0.577 | 0.925 | No |  |
|  | AlexNet_3class | Threefold_CV_original_images | SCLC | ADC + SCC/SQCC | Three lung cancer types: ADC, SCC/SQCC, SCLC | Softmax/model output or downstream classifier class prediction | 81 | 26 | 3 | 179 | 0.964 | 0.873 | No |  |
|  | InceptionV3_3class | Threefold_CV_original_images | ADC | SCC/SQCC + SCLC | Three lung cancer types: ADC, SCC/SQCC, SCLC | Softmax/model output or downstream classifier class prediction | 46 | 49 | 29 | 165 | 0.613 | 0.771 | No |  |
|  | InceptionV3_3class | Threefold_CV_original_images | SCC/SQCC | ADC + SCLC | Three lung cancer types: ADC, SCC/SQCC, SCLC | Softmax/model output or downstream classifier class prediction | 80 | 21 | 50 | 138 | 0.615 | 0.868 | No |  |
|  | InceptionV3_3class | Threefold_CV_original_images | SCLC | ADC + SCC/SQCC | Three lung cancer types: ADC, SCC/SQCC, SCLC | Softmax/model output or downstream classifier class prediction | 67 | 26 | 17 | 179 | 0.798 | 0.873 | No |  |
|  | VGG16_3class | Threefold_CV_original_images | ADC | SCC/SQCC + SCLC | Three lung cancer types: ADC, SCC/SQCC, SCLC | Softmax/model output or downstream classifier class prediction | 56 | 28 | 19 | 186 | 0.747 | 0.869 | No |  |
|  | VGG16_3class | Threefold_CV_original_images | SCC/SQCC | ADC + SCLC | Three lung cancer types: ADC, SCC/SQCC, SCLC | Softmax/model output or downstream classifier class prediction | 89 | 16 | 41 | 143 | 0.685 | 0.899 | No |  |
|  | VGG16_3class | Threefold_CV_original_images | SCLC | ADC + SCC/SQCC | Three lung cancer types: ADC, SCC/SQCC, SCLC | Softmax/model output or downstream classifier class prediction | 77 | 23 | 7 | 182 | 0.917 | 0.888 | No |  |
|  | ResNet50_3class | Threefold_CV_original_images | ADC | SCC/SQCC + SCLC | Three lung cancer types: ADC, SCC/SQCC, SCLC | Softmax/model output or downstream classifier class prediction | 56 | 35 | 19 | 179 | 0.747 | 0.836 | No |  |
|  | ResNet50_3class | Threefold_CV_original_images | SCC/SQCC | ADC + SCLC | Three lung cancer types: ADC, SCC/SQCC, SCLC | Softmax/model output or downstream classifier class prediction | 88 | 23 | 42 | 136 | 0.677 | 0.855 | No |  |
|  | ResNet50_3class | Threefold_CV_original_images | SCLC | ADC + SCC/SQCC | Three lung cancer types: ADC, SCC/SQCC, SCLC | Softmax/model output or downstream classifier class prediction | 70 | 17 | 14 | 188 | 0.833 | 0.917 | No |  |
|  | NB_DCNN_probs_3class | Threefold_CV_original_images | ADC | SCC/SQCC + SCLC | Three lung cancer types: ADC, SCC/SQCC, SCLC | Softmax/model output or downstream classifier class prediction | 59 | 35 | 16 | 179 | 0.787 | 0.836 | No |  |
|  | NB_DCNN_probs_3class | Threefold_CV_original_images | SCC/SQCC | ADC + SCLC | Three lung cancer types: ADC, SCC/SQCC, SCLC | Softmax/model output or downstream classifier class prediction | 92 | 24 | 38 | 135 | 0.708 | 0.849 | No |  |
|  | NB_DCNN_probs_3class | Threefold_CV_original_images | SCLC | ADC + SCC/SQCC | Three lung cancer types: ADC, SCC/SQCC, SCLC | Softmax/model output or downstream classifier class prediction | 66 | 13 | 18 | 192 | 0.786 | 0.937 | No |  |
|  | SVM_DCNN_probs_3class | Threefold_CV_original_images | ADC | SCC/SQCC + SCLC | Three lung cancer types: ADC, SCC/SQCC, SCLC | Softmax/model output or downstream classifier class prediction | 44 | 15 | 31 | 199 | 0.587 | 0.930 | No |  |
|  | SVM_DCNN_probs_3class | Threefold_CV_original_images | SCC/SQCC | ADC + SCLC | Three lung cancer types: ADC, SCC/SQCC, SCLC | Softmax/model output or downstream classifier class prediction | 110 | 40 | 20 | 119 | 0.846 | 0.748 | No |  |
|  | SVM_DCNN_probs_3class | Threefold_CV_original_images | SCLC | ADC + SCC/SQCC | Three lung cancer types: ADC, SCC/SQCC, SCLC | Softmax/model output or downstream classifier class prediction | 70 | 10 | 14 | 195 | 0.833 | 0.951 | No |  |
|  | RF_DCNN_probs_3class | Threefold_CV_original_images | ADC | SCC/SQCC + SCLC | Three lung cancer types: ADC, SCC/SQCC, SCLC | Softmax/model output or downstream classifier class prediction | 51 | 19 | 24 | 195 | 0.680 | 0.911 | No |  |
|  | RF_DCNN_probs_3class | Threefold_CV_original_images | SCC/SQCC | ADC + SCLC | Three lung cancer types: ADC, SCC/SQCC, SCLC | Softmax/model output or downstream classifier class prediction | 107 | 33 | 23 | 126 | 0.823 | 0.792 | No |  |
|  | RF_DCNN_probs_3class | Threefold_CV_original_images | SCLC | ADC + SCC/SQCC | Three lung cancer types: ADC, SCC/SQCC, SCLC | Softmax/model output or downstream classifier class prediction | 68 | 11 | 16 | 194 | 0.810 | 0.946 | No |  |
|  | NN_DCNN_probs_3class | Threefold_CV_original_images | ADC | SCC/SQCC + SCLC | Three lung cancer types: ADC, SCC/SQCC, SCLC | Softmax/model output or downstream classifier class prediction | 54 | 25 | 21 | 189 | 0.720 | 0.883 | Yes |  |
|  | NN_DCNN_probs_3class | Threefold_CV_original_images | SCC/SQCC | ADC + SCLC | Three lung cancer types: ADC, SCC/SQCC, SCLC | Softmax/model output or downstream classifier class prediction | 98 | 21 | 32 | 138 | 0.754 | 0.868 | Yes |  |
|  | NN_DCNN_probs_3class | Threefold_CV_original_images | SCLC | ADC + SCC/SQCC | Three lung cancer types: ADC, SCC/SQCC, SCLC | Softmax/model output or downstream classifier class prediction | 76 | 15 | 8 | 190 | 0.905 | 0.927 | Yes |  |
| Zhang et al., 2026 | CNN_LSTM_Raman_BM | Augmented_subtype_exploratory | ADC/LUAD | Other lung cancer subtypes plus non-cancer | Lung cancer subtype spectra plus non-cancer spectra; each subtype augmented to 810 spectra | Model-default CNN-LSTM multiclass output | NR | NR | NR | NR | NR | NR | No | ^30^ |
|  | CNN_LSTM_Raman_BM | Augmented_subtype_exploratory | SCC/SQCC | Other lung cancer subtypes plus non-cancer | Lung cancer subtype spectra plus non-cancer spectra; each subtype augmented to 810 spectra | Model-default CNN-LSTM multiclass output | NR | NR | NR | NR | NR | NR | No |  |
|  | CNN_LSTM_Raman_BM | Augmented_subtype_exploratory | SCLC | Other lung cancer subtypes plus non-cancer | Lung cancer subtype spectra plus non-cancer spectra; each subtype augmented to 810 spectra | Model-default CNN-LSTM multiclass output | NR | NR | NR | NR | NR | NR | No |  |
| Zhao et al., 2025a | RIOM_ROSE_ImageOnly | Test_set_Fig4A_collapsed_malignant | ADC/LUAD | SCC/LUSC + SCLC + LCLC/Other | Malignant cases only; true benign row excluded; RIOM five-class matrix collapsed to ADC/SCC/SCLC vs rest; LCLC and predicted benign treated as Other | Five-class argmax collapsed one-vs-rest | 39 | 7 | 2 | 90 | 0.951 | 0.928 | No | ^31^ |
|  | RIOM_ROSE_ImageOnly | Test_set_Fig4A_collapsed_malignant | SCC/LUSC | ADC/LUAD + SCLC + LCLC/Other | Malignant cases only; true benign row excluded; RIOM five-class matrix collapsed to ADC/SCC/SCLC vs rest; LCLC and predicted benign treated as Other | Five-class argmax collapsed one-vs-rest | 34 | 5 | 9 | 90 | 0.791 | 0.947 | No |  |
|  | RIOM_ROSE_ImageOnly | Test_set_Fig4A_collapsed_malignant | SCLC | ADC/LUAD + SCC/LUSC + LCLC/Other | Malignant cases only; true benign row excluded; RIOM five-class matrix collapsed to ADC/SCC/SCLC vs rest; LCLC and predicted benign treated as Other | Five-class argmax collapsed one-vs-rest | 18 | 1 | 2 | 117 | 0.900 | 0.992 | No |  |
|  | RSMM_ROSE_Serum | Test_set_Fig4B_collapsed_malignant | ADC/LUAD | SCC/LUSC + SCLC + LCLC/Other | Malignant cases only; true benign row excluded; RSMM five-class matrix collapsed to ADC/SCC/SCLC vs rest; LCLC and predicted benign treated as Other | Five-class argmax collapsed one-vs-rest | 41 | 0 | 0 | 97 | 1.000 | 1.000 | Yes |  |
|  | RSMM_ROSE_Serum | Test_set_Fig4B_collapsed_malignant | SCC/LUSC | ADC/LUAD + SCLC + LCLC/Other | Malignant cases only; true benign row excluded; RSMM five-class matrix collapsed to ADC/SCC/SCLC vs rest; LCLC and predicted benign treated as Other | Five-class argmax collapsed one-vs-rest | 43 | 0 | 0 | 95 | 1.000 | 1.000 | Yes |  |
|  | RSMM_ROSE_Serum | Test_set_Fig4B_collapsed_malignant | SCLC | ADC/LUAD + SCC/LUSC + LCLC/Other | Malignant cases only; true benign row excluded; RSMM five-class matrix collapsed to ADC/SCC/SCLC vs rest; LCLC and predicted benign treated as Other | Five-class argmax collapsed one-vs-rest | 12 | 0 | 8 | 118 | 0.600 | 1.000 | Yes |  |

ADC, adenocarcinoma; LUAD, lung adenocarcinoma; SCC, squamous cell carcinoma; LUSC, lung squamous cell carcinoma; SCLC, small cell lung cancer; NSCLC-NOS, non-small cell lung cancer not otherwise specified; HMLCS, hierarchical multi-label classification system; LCNEC, large cell neuroendocrine carcinoma; NSCLC, non-small cell lung cancer; SQCC, squamous cell carcinoma; KF, Kalman filter; GCN, graph convolutional network; DCNN, deep convolutional neural network; NB, naïve Bayes; SVM, support vector machine; RF, random forest; NN, neural network; CNN-LSTM, convolutional neural network long short-term memory; LCLC, large cell lung carcinoma; RSMM, ROI serum multimodal model; RIOM, ROI image-only model

## Supplementary Table S9. Multiclass ADC/SCC/SCLC confusion matrix extraction layout.

| **Study ID** | **Model ID** | **Dataset ID** | **True class** | **Predicted ADC** | **Predicted SCC** | **Predicted SCLC** | **Predicted other** | **Total true class** | **Recall** | **Reference** |
| --- | --- | --- | --- | --- | --- | --- | --- | --- | --- | --- |
| Chen et al., 2024 | HMLCS_ROSE_Serum | Test_set_final_collapsed | ADC | 58 | 3 | 0 | 6 | 67 | 0.866 | ^5^ |
|  | HMLCS_ROSE_Serum | Test_set_final_collapsed | SCC | 8 | 15 | 3 | 3 | 29 | 0.517 |  |
|  | HMLCS_ROSE_Serum | Test_set_final_collapsed | SCLC | 4 | 1 | 26 | 1 | 32 | 0.812 |  |
|  | HMLCS_ROSE_Serum | Test_set_final_collapsed | Other/benign | 5 | 1 | 3 | 9 | 18 | 0.500 |  |
| Tanaka et al., 2025 | DenseNet121_LBC_DCNN | Case_level_5fold_CV_ADC_SQCC | ADC | 18 | 4 | 0 | 0 | 22 | 0.818 | ^18^ |
|  | DenseNet121_LBC_DCNN | Case_level_5fold_CV_ADC_SQCC | SCC | 4 | 11 | 0 | 0 | 15 | 0.733 |  |
| Teramoto et al., 2017 | DCNN_3class_LungCytology | Threefold_CV_original_images | ADC | 73 | 8 | 1 | 0 | 82 | 0.890 | ^20^ |
|  | DCNN_3class_LungCytology | Threefold_CV_original_images | SCC | 35 | 75 | 15 | 0 | 125 | 0.600 |  |
|  | DCNN_3class_LungCytology | Threefold_CV_original_images | SCLC | 7 | 20 | 64 | 0 | 91 | 0.703 |  |
| Teramoto et al., 2022 | AlexNet_3class | Threefold_CV_original_images | ADC | 57 | 10 | 8 | 0 | 75 | 0.760 | ^23^ |
|  | AlexNet_3class | Threefold_CV_original_images | SCC | 37 | 75 | 18 | 0 | 130 | 0.577 |  |
|  | AlexNet_3class | Threefold_CV_original_images | SCLC | 1 | 2 | 81 | 0 | 84 | 0.964 |  |
|  | InceptionV3_3class | Threefold_CV_original_images | ADC | 46 | 17 | 12 | 0 | 75 | 0.613 |  |
|  | InceptionV3_3class | Threefold_CV_original_images | SCC | 36 | 80 | 14 | 0 | 130 | 0.615 |  |
|  | InceptionV3_3class | Threefold_CV_original_images | SCLC | 13 | 4 | 67 | 0 | 84 | 0.798 |  |
|  | VGG16_3class | Threefold_CV_original_images | ADC | 56 | 12 | 7 | 0 | 75 | 0.747 |  |
|  | VGG16_3class | Threefold_CV_original_images | SCC | 25 | 89 | 16 | 0 | 130 | 0.685 |  |
|  | VGG16_3class | Threefold_CV_original_images | SCLC | 3 | 4 | 77 | 0 | 84 | 0.917 |  |
|  | ResNet50_3class | Threefold_CV_original_images | ADC | 56 | 14 | 5 | 0 | 75 | 0.747 |  |
|  | ResNet50_3class | Threefold_CV_original_images | SCC | 30 | 88 | 12 | 0 | 130 | 0.677 |  |
|  | ResNet50_3class | Threefold_CV_original_images | SCLC | 5 | 9 | 70 | 0 | 84 | 0.833 |  |
|  | NB_DCNN_probs_3class | Threefold_CV_original_images | ADC | 59 | 11 | 5 | 0 | 75 | 0.787 |  |
|  | NB_DCNN_probs_3class | Threefold_CV_original_images | SCC | 30 | 92 | 8 | 0 | 130 | 0.708 |  |
|  | NB_DCNN_probs_3class | Threefold_CV_original_images | SCLC | 5 | 13 | 66 | 0 | 84 | 0.786 |  |
|  | SVM_DCNN_probs_3class | Threefold_CV_original_images | ADC | 44 | 26 | 5 | 0 | 75 | 0.587 |  |
|  | SVM_DCNN_probs_3class | Threefold_CV_original_images | SCC | 15 | 110 | 5 | 0 | 130 | 0.846 |  |
|  | SVM_DCNN_probs_3class | Threefold_CV_original_images | SCLC | 0 | 14 | 70 | 0 | 84 | 0.833 |  |
|  | RF_DCNN_probs_3class | Threefold_CV_original_images | ADC | 51 | 19 | 5 | 0 | 75 | 0.680 |  |
|  | RF_DCNN_probs_3class | Threefold_CV_original_images | SCC | 17 | 107 | 6 | 0 | 130 | 0.823 |  |
|  | RF_DCNN_probs_3class | Threefold_CV_original_images | SCLC | 2 | 14 | 68 | 0 | 84 | 0.810 |  |
|  | NN_DCNN_probs_3class | Threefold_CV_original_images | ADC | 54 | 17 | 4 | 0 | 75 | 0.720 |  |
|  | NN_DCNN_probs_3class | Threefold_CV_original_images | SCC | 21 | 98 | 11 | 0 | 130 | 0.754 |  |
|  | NN_DCNN_probs_3class | Threefold_CV_original_images | SCLC | 4 | 4 | 76 | 0 | 84 | 0.905 |  |
| Zhao et al., 2025a | RIOM_ROSE_ImageOnly | Test_set_Fig4A_collapsed_malignant | ADC | 39 | 1 | 0 | 1 | 41 | 0.951 | ^31^ |
|  | RIOM_ROSE_ImageOnly | Test_set_Fig4A_collapsed_malignant | SCC | 7 | 34 | 0 | 2 | 43 | 0.791 |  |
|  | RIOM_ROSE_ImageOnly | Test_set_Fig4A_collapsed_malignant | SCLC | 0 | 2 | 18 | 0 | 20 | 0.900 |  |
|  | RIOM_ROSE_ImageOnly | Test_set_Fig4A_collapsed_malignant | Other/benign | 0 | 2 | 1 | 31 | 34 | 0.912 |  |
|  | RSMM_ROSE_Serum | Test_set_Fig4B_collapsed_malignant | ADC | 41 | 0 | 0 | 0 | 41 | 1.000 |  |
|  | RSMM_ROSE_Serum | Test_set_Fig4B_collapsed_malignant | SCC | 0 | 43 | 0 | 0 | 43 | 1.000 |  |
|  | RSMM_ROSE_Serum | Test_set_Fig4B_collapsed_malignant | SCLC | 0 | 0 | 12 | 8 | 20 | 0.600 |  |
|  | RSMM_ROSE_Serum | Test_set_Fig4B_collapsed_malignant | Other/benign | 0 | 0 | 0 | 34 | 34 | 1.000 |  |

CV, cross-validation; DCNN, deep convolutional neural network

## Supplementary Table S10. Reported performance metrics beyond 2x2 DTA data.

| **Study ID** | **Model ID** | **Task** | **Dataset** | **Accuracy** | **AUC** | **Precision/PPV** | **Recall/sensitivity** | **F1 score** | **Kappa** | **Calibration/other** | **Reference** |
| --- | --- | --- | --- | --- | --- | --- | --- | --- | --- | --- | --- |
| Ai et al., 2022 | AI_ROSE_DenseNet169 | Malignant vs benign | Testing_group | 0.846 | NR | 0.955 | 0.800 | 0.870 | NR | NPV 0.716; LR+ 11.4; LR- 0.215 | ^1^ |
| Chen et al., 2020 | EfficientNet_GBRT_CNN | Cancer/abnormal vs normal | Not_reported | NR | 0.970 | NR | NR | 0.970 | NR | NR | ^2^ |
| Chen et al., 2021a | ResNet101_BM | Malignant vs benign | Test_patches | 0.988 | NR | 0.991 | 0.988 | 0.989 | NR | NPV 0.983; LR+ 82.968; LR- 0.012 | ^3^ |
|  | ResNet101_BM | Malignant vs benign | Test_images | 0.955 | NR | 0.966 | 0.982 | 0.974 | NR | NPV 0.875; LR+ 4.421; LR- 0.023 |  |
|  | ResNet101_BM | Malignant vs benign | Test_patients | 0.929 | NR | 0.917 | 1.000 | 0.957 | NR | NPV 1.000; LR+ 3; LR- 0 |  |
| Chen et al., 2021b | NIRS_PLS | Malignant pleural effusion vs benign pleural effusion | Test_set | 0.800 | NR | 0.769 | 0.909 | 0.833 | NR | NPV 0.857; LR+ 2.727; LR- 0.136 | ^4^ |
|  | NIRS_RF | Malignant pleural effusion vs benign pleural effusion | Test_set | 0.950 | NR | 0.917 | 1.000 | 0.957 | NR | NPV 1.000; LR+ 9; LR- 0 |  |
|  | NIRS_SVM | Malignant pleural effusion vs benign pleural effusion | Test_set | 1.000 | 1.000 | 1.000 | 1.000 | 1.000 | NR | NPV 1.000; LR- 0 |  |
|  | NIRS_GBM | Malignant pleural effusion vs benign pleural effusion | Test_set | 0.950 | NR | 1.000 | 0.909 | 0.952 | NR | NPV 0.900; LR- 0.091 |  |
| Chen et al., 2024 | HMLCS_ROSE_Serum | Malignant vs benign | Test_set_BM_module | 0.891 | 0.954 | 0.964 | 0.904 | 0.933 | NR | NPV 0.622; LR+ 5.063; LR- 0.117 | ^5^ |
|  | HMLCS_ROSE_Serum | ADC/LUAD vs rest | Test_set_final_collapsed | 0.822 | NR | 0.773 | 0.866 | 0.817 | NR | NPV 0.873; LR+ 4.023; LR- 0.171 |  |
|  | HMLCS_ROSE_Serum | SCC/LUSC vs rest | Test_set_final_collapsed | 0.870 | NR | 0.750 | 0.517 | 0.612 | NR | NPV 0.889; LR+ 12.103; LR- 0.504 |  |
|  | HMLCS_ROSE_Serum | SCLC vs rest | Test_set_final_collapsed | 0.918 | NR | 0.812 | 0.812 | 0.812 | NR | NPV 0.947; LR+ 15.437; LR- 0.198 |  |
|  | HMLCS_ROSE_Serum | SCLC vs rest | Test_set_M-sub_conditional | 0.917 | NR | 0.812 | 0.839 | 0.825 | NR | NPV 0.950; LR+ 14.118; LR- 0.171 |  |
|  | HMLCS_ROSE_Serum | ADC/LUAD vs rest | Test_set_NSCLC-sub_conditional | 0.860 | NR | 0.866 | 0.951 | 0.906 | NR | NPV 0.842; LR+ 2.641; LR- 0.077 |  |
|  | HMLCS_ROSE_Serum | SCC/LUSC vs rest | Test_set_NSCLC-sub_conditional | 0.872 | NR | 0.833 | 0.652 | 0.732 | NR | NPV 0.882; LR+ 13.696; LR- 0.365 |  |
| Ganesh et al., 2022 | SERS_PLSDA_Lung_vs_Control | Lung cancer-associated pleural effusion vs control effusion | CV_lung_vs_control | NR | 0.930 | NR | NR | NR | NR | NR | ^6^ |
|  | SERS_PLSDA_Lung_vs_Control | Any cancer-associated pleural effusion vs control effusion | CV_all_cancer_vs_control | NR | 0.880 | NR | NR | NR | NR | NR |  |
| Giarnieri et al., 2025 | YOLOv8_ADK_MS | Adenocarcinoma cells vs mesothelial cells | Test_annotations | NR | NR | NR | NR | 0.720 | NR | NR | ^7^ |
|  | YOLOv11_ADK_MS | Adenocarcinoma cells vs mesothelial cells | Test_annotations | NR | NR | NR | NR | 0.720 | NR | NR |  |
| Gong et al., 2025 | ResNet18_CBAM_ROSE | Six-class respiratory cytology diagnosis; binary malignant/benign not extractable | Test_116_images | NR | NR | NR | NR | NR | NR | NR | ^8^ |
| Gonzalez et al., 2020 | InceptionV3_DiffQuik_LCNEC | LCNEC vs rest | Test_DiffQuik | 0.889 | 1.000 | 0.500 | 1.000 | 0.667 | NR | NPV 1.000; LR+ 8; LR- 0 | ^9^ |
|  | InceptionV3_Pap_LCNEC | LCNEC vs rest | Test_Pap | 0.875 | 1.000 | 0.500 | 1.000 | 0.667 | NR | NPV 1.000; LR+ 7; LR- 0 |  |
|  | InceptionV3_HE_LCNEC | LCNEC vs rest | Test_HE | 0.900 | 0.875 | 0.667 | 1.000 | 0.800 | NR | NPV 1.000; LR+ 8; LR- 0 |  |
| Kim et al., 2023 | DenseNet121_BM | Lung cancer vs benign cells | Test_patches_AI | 0.969 | NR | 0.986 | 0.959 | 0.972 | NR | NPV 0.950; LR+ 53.441; LR- 0.041 | ^10^ |
|  | DenseNet121_BM | Lung cancer vs benign cells | Validation_HE | 0.986 | NR | 0.889 | 0.889 | 0.889 | NR | NPV 0.992; LR+ 118.444; LR- 0.112 |  |
|  | DenseNet121_BM | Lung cancer vs benign cells | Validation_PAP | 0.951 | NR | 0.949 | 0.957 | 0.953 | NR | NPV 0.953; LR+ 17.05; LR- 0.045 |  |
| Kim et al., 2025 | InceptionResNetV2_patch | Malignant effusion vs benign | Test_patches | 0.991 | NR | 0.987 | 0.996 | 0.992 | NR | NPV 0.996; LR+ 74.307; LR- 0.004 | ^11^ |
|  | CLAM_SB | Malignant effusion vs benign | Test_WSIs | 0.810 | 0.744 | 0.677 | 0.778 | 0.724 | NR | NPV 0.887; LR+ 4.433; LR- 0.27 |  |
|  | CLAM_MB | Malignant effusion vs benign | Test_WSIs | 0.905 | 0.942 | 0.880 | 0.815 | 0.846 | NR | NPV 0.915; LR+ 15.481; LR- 0.195 |  |
|  | CLAM_MB | Malignant effusion vs benign | External_QAP_WSIs | 0.903 | 0.947 | 0.948 | 0.902 | 0.924 | NR | NPV 0.829; LR+ 9.617; LR- 0.109 |  |
| Lai et al., 2025 | VIS_HSI_PCA | Malignant vs non-malignant/reactive cells | Twenty_four_cell_ROIs | NR | NR | NR | NR | NR | NR | NR | ^12^ |
| Lan et al., 2024 | CUNet3plus_ROSE_Seg | Cancer-cell cluster segmentation; not case-level B/M diagnosis | Internal_external_segmentation | NR | NR | NR | NR | 0.960 | NR | NR | ^13^ |
| Lemieux et al., 2023 | CyPath_Lung_GLM | Lung cancer vs non-cancer | LSRII_validation | 0.867 | 0.890 | 0.605 | 0.821 | 0.697 | NR | NPV 0.955; LR+ 6.681; LR- 0.204 | ^14^ |
|  | CyPath_Lung_GLM | Lung cancer vs non-cancer in small-nodule subset | LSRII_nodules_lt20mm | 0.879 | 0.940 | 0.444 | 0.923 | 0.600 | NR | NPV 0.990; LR+ 7.323; LR- 0.088 |  |
|  | CyPath_Lung_GLM | Lung cancer vs non-cancer | Navios_external | 0.781 | 0.850 | 0.455 | 0.833 | 0.588 | NR | NPV 0.952; LR+ 3.611; LR- 0.217 |  |
| Liu et al., 2023 | BALFilter_Reader_YOLOv5x | Lung cancer vs non-cancer | Clinical_cross_validation_24_cases | 0.750 | 0.840 | 1.000 | 0.667 | 0.800 | NR | NPV 0.500; LR- 0.333 | ^15^ |
| Taher et al., 2015 | ANN_Sputum_BM | Benign vs malignant sputum cells | Tenfold_CV_100_images | NR | NR | NR | NR | NR | NR | NR | ^16^ |
|  | SVM_Sputum_BM | Benign vs malignant sputum cells | Tenfold_CV_100_images | NR | NR | NR | NR | NR | NR | NR |  |
| Taher et al., 2023 | Bayesian_RuleCAD_Sputum | Cancerous vs normal sputum cells | CAD_100_images | 0.820 | NR | 0.867 | 0.890 | 0.878 | NR | NPV 0.680; LR+ 2.404; LR- 0.174 | ^17^ |
| Tanaka et al., 2025 | DenseNet121_LBC_DCNN | Malignant (ADC+SQCC) vs normal | Patch_level_5fold_CV_BM | 0.936 | NR | 0.939 | 0.972 | 0.955 | NR | NPV 0.929; LR+ 6.571; LR- 0.032 | ^18^ |
|  | DenseNet121_LBC_DCNN | Malignant (ADC+SQCC) vs normal | Case_level_5fold_CV_BM | 0.911 | NR | 0.971 | 0.919 | 0.944 | NR | NPV 0.700; LR+ 7.351; LR- 0.093 |  |
|  | DenseNet121_LBC_DCNN | ADC vs rest | Patch_level_5fold_CV_ADC_SQCC | 0.899 | NR | 0.949 | 0.914 | 0.931 | NR | NPV 0.775; LR+ 6.434; LR- 0.101 |  |
|  | DenseNet121_LBC_DCNN | SCC/SQCC vs rest | Patch_level_5fold_CV_ADC_SQCC | 0.899 | NR | 0.775 | 0.858 | 0.814 | NR | NPV 0.949; LR+ 9.929; LR- 0.155 |  |
|  | DenseNet121_LBC_DCNN | ADC vs rest | Case_level_5fold_CV_ADC_SQCC | 0.784 | NR | 0.818 | 0.818 | 0.818 | NR | NPV 0.733; LR+ 3.068; LR- 0.248 |  |
|  | DenseNet121_LBC_DCNN | SCC/SQCC vs rest | Case_level_5fold_CV_ADC_SQCC | 0.784 | NR | 0.733 | 0.733 | 0.733 | NR | NPV 0.818; LR+ 4.033; LR- 0.326 |  |
| Tang et al., 2024 | DualBranch_KF_GCN_CNN | ADC/SCC binary task vs rest | External_test_40_images | NR | 0.980 | NR | NR | 0.920 | NR | NR | ^19^ |
| Teramoto et al., 2017 | DCNN_3class_LungCytology | ADC vs rest | Threefold_CV_original_images | 0.829 | NR | 0.635 | 0.890 | 0.741 | NR | NPV 0.951; LR+ 4.578; LR- 0.136 | ^20^ |
|  | DCNN_3class_LungCytology | SCC/SQCC vs rest | Threefold_CV_original_images | 0.738 | NR | 0.728 | 0.600 | 0.658 | NR | NPV 0.744; LR+ 3.707; LR- 0.477 |  |
|  | DCNN_3class_LungCytology | SCLC vs rest | Threefold_CV_original_images | 0.856 | NR | 0.800 | 0.703 | 0.749 | NR | NPV 0.876; LR+ 9.099; LR- 0.322 |  |
| Teramoto et al., 2019 | VGG16_BM_aug | Malignant vs benign lung cytology patches | Patch_level_3fold_CV_no_aug | 0.654 | NR | 0.600 | 0.949 | 0.736 | NR | NPV 0.870; LR+ 1.46; LR- 0.145 | ^21^ |
|  | VGG16_BM_aug | Malignant vs benign lung cytology cases | Case_level_3fold_CV_no_aug | 0.761 | NR | 0.743 | 0.929 | 0.825 | NR | NPV 0.818; LR+ 1.857; LR- 0.143 |  |
|  | VGG16_BM_aug | Malignant vs benign lung cytology patches | Patch_level_3fold_CV_augmented | 0.792 | 0.872 | 0.727 | 0.946 | 0.822 | NR | NPV 0.919; LR+ 2.585; LR- 0.085 |  |
|  | VGG16_BM_aug | Malignant vs benign lung cytology cases | Case_level_3fold_CV_augmented | 0.870 | 0.886 | 0.893 | 0.893 | 0.893 | NR | NPV 0.833; LR+ 5.357; LR- 0.129 |  |
| Teramoto et al., 2020 | VGG16_PGGAN_BM | Malignant vs benign lung cytology patches | Patch_level_3fold_CV_PGGAN | 0.853 | 0.901 | 0.856 | 0.854 | 0.855 | NR | NPV 0.850; LR+ 5.804; LR- 0.172 | ^22^ |
|  | AlexNet_3class | ADC vs rest | Threefold_CV_original_images | 0.806 | NR | 0.600 | 0.760 | 0.671 | NR | NPV 0.907; LR+ 4.28; LR- 0.292 |  |
|  | AlexNet_3class | SCC/SQCC vs rest | Threefold_CV_original_images | 0.768 | NR | 0.862 | 0.577 | 0.691 | NR | NPV 0.728; LR+ 7.644; LR- 0.458 |  |
|  | AlexNet_3class | SCLC vs rest | Threefold_CV_original_images | 0.900 | NR | 0.757 | 0.964 | 0.848 | NR | NPV 0.984; LR+ 7.603; LR- 0.041 |  |
|  | InceptionV3_3class | ADC vs rest | Threefold_CV_original_images | 0.730 | NR | 0.484 | 0.613 | 0.541 | NR | NPV 0.851; LR+ 2.679; LR- 0.501 |  |
|  | InceptionV3_3class | SCC/SQCC vs rest | Threefold_CV_original_images | 0.754 | NR | 0.792 | 0.615 | 0.693 | NR | NPV 0.734; LR+ 4.659; LR- 0.443 |  |
|  | InceptionV3_3class | SCLC vs rest | Threefold_CV_original_images | 0.851 | NR | 0.720 | 0.798 | 0.757 | NR | NPV 0.913; LR+ 6.289; LR- 0.232 |  |
|  | VGG16_3class | ADC vs rest | Threefold_CV_original_images | 0.837 | NR | 0.667 | 0.747 | 0.704 | NR | NPV 0.907; LR+ 5.707; LR- 0.291 |  |
|  | VGG16_3class | SCC/SQCC vs rest | Threefold_CV_original_images | 0.803 | NR | 0.848 | 0.685 | 0.757 | NR | NPV 0.777; LR+ 6.803; LR- 0.351 |  |
|  | VGG16_3class | SCLC vs rest | Threefold_CV_original_images | 0.896 | NR | 0.770 | 0.917 | 0.837 | NR | NPV 0.963; LR+ 8.17; LR- 0.094 |  |
|  | ResNet50_3class | ADC vs rest | Threefold_CV_original_images | 0.813 | NR | 0.615 | 0.747 | 0.675 | NR | NPV 0.904; LR+ 4.565; LR- 0.303 |  |
|  | ResNet50_3class | SCC/SQCC vs rest | Threefold_CV_original_images | 0.775 | NR | 0.793 | 0.677 | 0.730 | NR | NPV 0.764; LR+ 4.68; LR- 0.378 |  |
|  | ResNet50_3class | SCLC vs rest | Threefold_CV_original_images | 0.893 | NR | 0.805 | 0.833 | 0.819 | NR | NPV 0.931; LR+ 10.049; LR- 0.182 |  |
|  | NB_DCNN_probs_3class | ADC vs rest | Threefold_CV_original_images | 0.824 | NR | 0.628 | 0.787 | 0.698 | NR | NPV 0.918; LR+ 4.81; LR- 0.255 |  |
|  | NB_DCNN_probs_3class | SCC/SQCC vs rest | Threefold_CV_original_images | 0.785 | NR | 0.793 | 0.708 | 0.748 | NR | NPV 0.780; LR+ 4.688; LR- 0.344 |  |
|  | NB_DCNN_probs_3class | SCLC vs rest | Threefold_CV_original_images | 0.893 | NR | 0.835 | 0.786 | 0.810 | NR | NPV 0.914; LR+ 12.39; LR- 0.229 |  |
|  | SVM_DCNN_probs_3class | ADC vs rest | Threefold_CV_original_images | 0.841 | NR | 0.746 | 0.587 | 0.657 | NR | NPV 0.865; LR+ 8.37; LR- 0.444 |  |
|  | SVM_DCNN_probs_3class | SCC/SQCC vs rest | Threefold_CV_original_images | 0.792 | NR | 0.733 | 0.846 | 0.786 | NR | NPV 0.856; LR+ 3.363; LR- 0.206 |  |
|  | SVM_DCNN_probs_3class | SCLC vs rest | Threefold_CV_original_images | 0.917 | NR | 0.875 | 0.833 | 0.854 | NR | NPV 0.933; LR+ 17.083; LR- 0.175 |  |
|  | RF_DCNN_probs_3class | ADC vs rest | Threefold_CV_original_images | 0.851 | NR | 0.729 | 0.680 | 0.703 | NR | NPV 0.890; LR+ 7.659; LR- 0.351 |  |
|  | RF_DCNN_probs_3class | SCC/SQCC vs rest | Threefold_CV_original_images | 0.806 | NR | 0.764 | 0.823 | 0.793 | NR | NPV 0.846; LR+ 3.966; LR- 0.223 |  |
|  | RF_DCNN_probs_3class | SCLC vs rest | Threefold_CV_original_images | 0.907 | NR | 0.861 | 0.810 | 0.834 | NR | NPV 0.924; LR+ 15.087; LR- 0.201 |  |
|  | NN_DCNN_probs_3class | ADC vs rest | Threefold_CV_original_images | 0.841 | NR | 0.684 | 0.720 | 0.701 | NR | NPV 0.900; LR+ 6.163; LR- 0.317 |  |
|  | NN_DCNN_probs_3class | SCC/SQCC vs rest | Threefold_CV_original_images | 0.817 | NR | 0.824 | 0.754 | 0.787 | NR | NPV 0.812; LR+ 5.708; LR- 0.284 |  |
|  | NN_DCNN_probs_3class | SCLC vs rest | Threefold_CV_original_images | 0.920 | NR | 0.835 | 0.905 | 0.869 | NR | NPV 0.960; LR+ 12.365; LR- 0.103 |  |
| Tondo et al., 2025 | AIROSE_InceptionV3_NN | Lung cancer-positive vs negative ROSE images | Positive_negative_10fold_CV | 0.925 | 0.948 | 0.940 | 0.964 | 0.952 | NR | NPV 0.865; LR+ 4.579; LR- 0.045 | ^24^ |
| Wang et al., 2025a | YOLOv8_SE_SIC_BM | Malignant vs benign/non-malignant lung cytology images | Additional_SIC_image_level | 0.910 | 0.900 | 0.923 | 0.956 | 0.939 | NR | NPV 0.870; LR+ 4.513; LR- 0.056 | ^25^ |
| Wang et al., 2025b | SERS_CEA_LDA | Lung cancer vs non-cancer pleural effusion | CV_lung_vs_non_cancer | 0.814 | 0.895 | 0.706 | 0.800 | 0.750 | NR | NPV 0.885; LR+ 4.48; LR- 0.243 | ^26^ |
|  | SERS_CEA_LDA | Other cancers vs non-cancer pleural effusion | CV_other_cancer_vs_non_cancer | 0.711 | 0.781 | 0.463 | 0.625 | 0.532 | NR | NPV 0.847; LR+ 2.414; LR- 0.506 |  |
|  | SERS_CEA_LDA | Lung cancer vs other cancers | CV_lung_vs_other_cancers | 0.710 | 0.848 | 0.792 | 0.700 | 0.743 | NR | NPV 0.617; LR+ 2.545; LR- 0.414 |  |
|  | SERS_CEA_SVM | Lung cancer vs non-cancer pleural effusion | CV_lung_vs_non_cancer | 0.953 | 0.970 | 1.000 | 0.867 | 0.929 | NR | NPV 0.933; LR- 0.133 |  |
|  | SERS_CEA_SVM | Other cancers vs non-cancer pleural effusion | CV_other_cancer_vs_non_cancer | 0.816 | 0.810 | 0.800 | 0.400 | 0.533 | NR | NPV 0.818; LR+ 11.2; LR- 0.622 |  |
|  | SERS_CEA_SVM | Lung cancer vs other cancers | CV_lung_vs_other_cancers | 0.910 | 0.980 | 0.918 | 0.933 | 0.926 | NR | NPV 0.897; LR+ 7.467; LR- 0.076 |  |
|  | SERS_CEA_KNN | Lung cancer vs non-cancer pleural effusion | CV_lung_vs_non_cancer | 0.866 | 0.950 | 0.776 | 0.867 | 0.819 | NR | NPV 0.924; LR+ 6.471; LR- 0.154 |  |
|  | SERS_CEA_KNN | Other cancers vs non-cancer pleural effusion | CV_other_cancer_vs_non_cancer | 0.855 | 0.840 | 0.645 | 1.000 | 0.784 | NR | NPV 1.000; LR+ 5.091; LR- 0 |  |
|  | SERS_CEA_KNN | Lung cancer vs other cancers | CV_lung_vs_other_cancers | 0.880 | 0.960 | 0.853 | 0.967 | 0.906 | NR | NPV 0.938; LR+ 3.867; LR- 0.044 |  |
| Wilbur et al., 2015 | LuCED_CellCT_BM | Lung cancer vs nonmalignant/no known malignancy | Clinical_study_91_specimens | 0.934 | NR | 0.957 | 0.918 | 0.938 | NR | NPV 0.909; LR+ 19.286; LR- 0.086 | ^27^ |
| Xie et al., 2022 | Aitrox_ResNet18_WSI_BM | Malignant vs benign pleural effusion cytology | Test_60_WSIs | 0.917 | 0.953 | 0.913 | 0.875 | 0.894 | NR | NPV 0.919; LR+ 15.75; LR- 0.132 | ^28^ |
| Yan et al., 2024 | ROSE_AI_ResNet101_BM | Cancer image vs non-cancer image | Internal_testing_images | 0.930 | NR | 0.947 | 0.944 | 0.945 | NR | NPV 0.899; LR+ 9.807; LR- 0.062 | ^29^ |
|  | ROSE_AI_ResNet101_BM | Cancer image vs non-cancer image | External_testing_images | 0.903 | NR | 0.957 | 0.908 | 0.932 | NR | NPV 0.778; LR+ 8.169; LR- 0.104 |  |
|  | ROSE_AI_ResNet101_BM | Lung cancer vs non-cancer | Internal_testing_patients | 0.896 | NR | 0.928 | 0.906 | 0.916 | NR | NPV 0.846; LR+ 7.547; LR- 0.107 |  |
|  | ROSE_AI_ResNet101_BM | Lung cancer vs non-cancer | External_testing_patients | 0.876 | NR | 0.877 | 0.949 | 0.912 | NR | NPV 0.872; LR+ 3.431; LR- 0.071 |  |
| Zhang et al., 2026 | CNN_LSTM_Raman_BM | Lung cancer vs non-cancer | Independent_test_190_spectra | 0.953 | 0.990 | 0.788 | 0.929 | 0.852 | NR | NPV 0.987; LR+ 21.49; LR- 0.075 | ^30^ |
|  | CNN_LSTM_Raman_BM | ADC/LUAD vs rest | Augmented_subtype_exploratory | NR | NR | NR | NR | NR | NR | NR |  |
|  | CNN_LSTM_Raman_BM | SCC/SQCC vs rest | Augmented_subtype_exploratory | NR | NR | NR | NR | NR | NR | NR |  |
|  | CNN_LSTM_Raman_BM | SCLC vs rest | Augmented_subtype_exploratory | NR | NR | NR | NR | NR | NR | NR |  |
| Zhao et al., 2025a | RIOM_ROSE_ImageOnly | Malignant lung lesion vs benign | Test_set_Fig4A_collapsed_BM | 0.942 | NR | 0.945 | 0.993 | 0.968 | NR | NPV 0.900; LR+ 2.11; LR- 0.014 | ^31^ |
|  | RSMM_ROSE_Serum | Malignant lung lesion vs benign | Test_set_Fig4B_collapsed_BM | 0.955 | NR | 0.971 | 0.978 | 0.975 | NR | NPV 0.812; LR+ 4.158; LR- 0.028 |  |
|  | RIOM_ROSE_ImageOnly | ADC/LUAD vs rest | Test_set_Fig4A_collapsed_malignant | 0.935 | NR | 0.848 | 0.951 | 0.897 | NR | NPV 0.978; LR+ 13.181; LR- 0.053 |  |
|  | RIOM_ROSE_ImageOnly | SCC/LUSC vs rest | Test_set_Fig4A_collapsed_malignant | 0.899 | NR | 0.872 | 0.791 | 0.829 | NR | NPV 0.909; LR+ 15.023; LR- 0.221 |  |
|  | RIOM_ROSE_ImageOnly | SCLC vs rest | Test_set_Fig4A_collapsed_malignant | 0.978 | NR | 0.947 | 0.900 | 0.923 | NR | NPV 0.983; LR+ 106.2; LR- 0.101 |  |
|  | RSMM_ROSE_Serum | ADC/LUAD vs rest | Test_set_Fig4B_collapsed_malignant | 1.000 | NR | 1.000 | 1.000 | 1.000 | NR | NPV 1.000; LR- 0 |  |
|  | RSMM_ROSE_Serum | SCC/LUSC vs rest | Test_set_Fig4B_collapsed_malignant | 1.000 | NR | 1.000 | 1.000 | 1.000 | NR | NPV 1.000; LR- 0 |  |
|  | RSMM_ROSE_Serum | SCLC vs rest | Test_set_Fig4B_collapsed_malignant | 0.942 | NR | 1.000 | 0.600 | 0.750 | NR | NPV 0.937; LR- 0.4 |  |
| Zhao et al., 2025b | LESSEL_WSI_BM | Lung cancer vs control/benign pulmonary disease | Discovery_cohort_cutoff_derivation | 0.617 | NR | 1.000 | 0.449 | 0.620 | NR | NPV 0.443; LR- 0.551 | ^32^ |
|  | LESSEL_WSI_BM | Lung cancer vs control/benign pulmonary disease | Same_center_validation | 0.648 | NR | 0.976 | 0.476 | 0.640 | NR | NPV 0.494; LR+ 20.952; LR- 0.536 |  |
|  | LESSEL_WSI_BM | Lung cancer vs control/benign pulmonary disease | External_validation_center2 | 0.746 | NR | 0.907 | 0.600 | 0.722 | NR | NPV 0.653; LR+ 7.95; LR- 0.433 |  |

AUC, area under the curve; PPV, positive predictive value; NPV, negative predictive value; LR+, positive likelihood ratio; LR-, negative likelihood ratio; NR, not reported

## Supplementary Table S11. Threshold, index test, and reference standard details.

| **Study ID** | **Task** | **AI score/output** | **Threshold value or rule** | **Prespecified or optimized** | **Reference standard** | **Blinded reference standard?** | **Follow-up/adjudication** | **Unit of analysis** | **Reference** |
| --- | --- | --- | --- | --- | --- | --- | --- | --- | --- |
| Ai et al., 2022 | Benign–malignant | Patch probability from DenseNet-169; WSI classified by count of malignant patches | Patch malignant probability ≥0.81; WSI malignant if >6 malignant patches | Optimized | Comprehensive diagnosis with pathology/clinical data | Unclear | Follow-up: Not reported \| Senior cytopathologist / comprehensive diagnosis | Testing_group | ^1^ |
| Chen et al., 2020 | Cancer vs normal | CNN class output | Not reported | Not reported | Cytology label; details NR | Unclear | Follow-up: Not reported \| Not reported | Not_reported | ^2^ |
| Chen et al., 2021a | Benign–malignant | Patch Softmax output; image malignant if at least one patch >0.99; patient malignant by majority vote | Softmax threshold 0.99 for image-level malignant classification; patient-level majority vote | Optimized/model threshold | Formal cytopathologic results | Unclear | Follow-up: Not reported \| Final cytopathology | Test_patches/images/patients | ^3^ |
| Chen et al., 2021b | MPE vs BPE | Class probabilities/decision functions from ML models applied to NIR spectra | Model default decision thresholds; parameters optimized by cross-validation | Optimized | MPE cytology/histology; BPE TB culture/clinical diagnosis | Unclear | Follow-up: Not reported \| Pathologic/clinical diagnosis; random train/test split | Test_set | ^4^ |
| Chen et al., 2024 | Benign–malignant | Hierarchical module probability/output for malignant vs benign | Model output/default threshold not explicitly prespecified | Model default | Final transbronchial sampling diagnosis with immunohistochemistry/follow-up | Unclear | Follow-up: At least 6 months for nonspecific benign findings \| Experienced cytopathologist labels accepted only when consistent with final diagnosis | Test_set_BM_module | ^5^ |
|  | ADC/SCC/SCLC subtyping | Hierarchical HMLCS final predicted class | Model output/default threshold; six-class final prediction collapsed to ADC/SCC/SCLC/Other for review | Model default | Final transbronchial sampling diagnosis with immunohistochemistry/follow-up | Unclear | Follow-up: At least 6 months for nonspecific benign findings \| Experienced cytopathologist labels accepted only when consistent with final diagnosis | Test_set_final_collapsed |  |
| Ganesh et al., 2022 | Lung cancer vs control pleural effusion | PLS-DA latent-variable class prediction from SERS spectrum | PLS-DA class assignment; threshold not separately reported | Optimized/model default | Clinical diagnosis of lung cancer vs control effusion | Unclear | Follow-up: Not reported \| Clinical grouping by study investigators | CV_lung_vs_control | ^6^ |
| Giarnieri et al., 2025 | Adenocarcinoma cell vs mesothelial cell | YOLO detection/segmentation confidence and mask localization | Object-detection confidence threshold; examples shown at 50% confidence; mAP evaluated over IoU thresholds | Model default/evaluation thresholds | Cytopathology and immunohistochemical confirmation/rule-out | Unclear | Follow-up: Not applicable \| Senior cytopathologist slide selection and manual annotations | Test_annotations | ^7^ |
|  | Adenocarcinoma cell vs mesothelial cell | YOLO detection/segmentation confidence and mask localization | Object-detection confidence threshold; examples shown at 50% confidence; mAP evaluated over IoU thresholds | Model default/evaluation thresholds | Cytopathology and immunohistochemical confirmation/rule-out | Unclear | Follow-up: Not applicable \| Senior cytopathologist slide selection and manual annotations | Test_annotations |  |
| Gong et al., 2025 | Six-class respiratory cytology diagnosis | ResNet-18/CBAM class probability output | Single-choice class output; threshold not reported | Model default | Biopsy/cell block/IHC for malignancies; biopsy/clinical/imaging adjudication for normal cases | Unclear | Follow-up: Not reported \| Consensus image selection by senior cytologists; chief physician arbitration | Test_116_images | ^8^ |
| Gonzalez et al., 2020 | LCNEC vs SCLC | Percent of slide tiles classified as LCNEC by InceptionV3 | >95% of patches labeled LCNEC to call slide LCNEC | Optimized ROC threshold | Original cytology diagnosis with IHC neuroendocrine confirmation | Unclear | Follow-up: Not reported \| Manual tumor annotation; original cytology report used as ground truth | Test_DiffQuik | ^9^ |
|  |  |  | >75% of patches labeled LCNEC to call slide LCNEC | Optimized ROC threshold | Original cytology diagnosis with IHC neuroendocrine confirmation | Unclear | Follow-up: Not reported \| Manual tumor annotation; original cytology report used as ground truth | Test_Pap |  |
|  |  |  | >80% of patches labeled LCNEC to call slide LCNEC | Optimized ROC threshold | Original cytology diagnosis with IHC neuroendocrine confirmation | Unclear | Follow-up: Not reported \| Manual tumor annotation; original cytology report used as ground truth | Test_HE |  |
| Kim et al., 2023 | Benign-malignant lung cancer cytology | DenseNet121 malignant/benign probability | Classification threshold 0.5 | Prespecified/model default | Corresponding histopathology and expert cytology patch review | Unclear | Follow-up: Not reported \| Patch labels reviewed by cytotechnologists/cytopathologists; disagreements excluded | Test_patches_AI | ^10^ |
| Kim et al., 2025 | Benign–malignant pleural effusion patch classification | Patch-level malignancy probability from Inception-ResNet-v2 | Sigmoid probability ≥0.5 | Prespecified/model default | Histology-confirmed malignant cases and expert cytology patch review | Unclear | Follow-up: Not reported \| More than 10 board-certified pathologists reviewed cytopathological and corresponding histopathological slides | Test_patches | ^11^ |
|  | Benign–malignant pleural effusion WSI classification | CLAM-MB WSI malignancy probability from attention MIL features | Model output/default threshold; sigmoid binary classification | Model default | Histology-confirmed malignant cases and board-certified pathologist review | Unclear | Follow-up: Not reported \| Board-certified pathologist review in Open AI Dataset and Quality Assurance Program | Test_WSIs/External_QAP_WSIs |  |
| Lai et al., 2025 | Normal/non-normal/malignant pleural-effusion cell feature extraction | Reconstructed transmittance spectra and principal component scores | No diagnostic threshold; PCA score-space separation only | No threshold | Board-certified cytologist cell classification | Unclear | Follow-up: Not reported \| Cytologist confirmed cells under 10× and 100× oil-immersion microscopy | Twenty_four_cell_ROIs | ^12^ |
| Lan et al., 2024 | Cancer-cell cluster segmentation in ROSE cytology images | Pixel-level segmentation probability/mask | Sigmoid segmentation output; explicit pixel threshold not reported | Model default/NR | Pathologist segmentation masks | Unclear | Follow-up: Not reported \| Experienced pathologists annotated cancer-cell cluster edges using LabelMe | Internal_external_segmentation | ^13^ |
| Lemieux et al., 2023 | Lung cancer vs non-cancer sputum classification | Logistic model value from age and FCM-derived density/marker variables | Model value >0.28 | Optimized by ROC on LSRII validation | Cancer confirmed by biopsy; non-cancer status from imaging/clinical classification | Unclear | Follow-up: Not fully reported \| Clinical cohort status confirmed; samples failing QC excluded | LSRII_validation | ^14^ |
|  | Lung cancer vs non-cancer sputum classification | The same logistic model value was applied to the independent Navios EX samples | Model value >0.5 | Optimized by ROC on the Navios sample set | Cancer confirmed by biopsy; non-cancer status from imaging/clinical classification | Unclear | Follow-up: Not fully reported \| Same model coefficients used for both instruments; cutoff differed for Navios ROC. | Navios_external |  |
| Liu et al., 2023 | BALF/BALFilter lung cancer diagnosis | Sum score S = Sts × N for cells with tumor suspiciousness score Sts ≥0.9 | S0.9 indicator with fold-specific ROC thresholds | Optimized/cross-validated | Histopathology results as the gold standard | Unclear | Follow-up: Not reported \| ROC thresholds selected from two groups and applied to the third group in threefold cross-validation | Clinical_cross_validation_24_cases | ^15^ |
| Taher et al., 2015 | Benign–malignant | ANN output class from handcrafted cytomorphologic features | ANN model output; threshold not explicitly reported | Not reported/model default | Pathologist-identified cells/diagnosis | Unclear | Follow-up: Not reported \| Not reported | Tenfold_CV_100_images | ^16^ |
|  |  | SVM output class from NC ratio, curvature, and circularity features | Gaussian kernel width σ=0.6; class decision by SVM margin | Optimized experimentally | Pathologist-identified cells/diagnosis | Unclear | Follow-up: Not reported \| Not reported | Tenfold_CV_100_images |  |
| Taher et al., 2023 | Benign–malignant | NC ratio and maximum drawable circle diagnostic rule outputs | λ=7 for segmentation; diagnostic rules T1=0.75, T2=0.85, T3=0.95, T4=300 | Optimized experimentally | Experimented pathologist diagnosis | Unclear | Follow-up: Not reported \| Not reported | CAD_100_images | ^17^ |
| Tanaka et al., 2025 | Benign–malignant and ADC/SQCC | Patch probability and case-level aggregate log-likelihood | Softmax/model default; case-level class selected by maximum sum of patch log-likelihoods | Model default | Final surgical pathology diagnosis with IHC/mucin stains, where needed | Unclear | Follow-up: Not applicable \| Two pathologists checked histopathological diagnoses; an experienced cytopathologist annotated patches | Patch_and_case_5fold_CV | ^18^ |
| Tang et al., 2024 | ADC/SCC subtyping | MLP/softmax output from fused GCN spatial features and CNN texture features | Softmax/model default; no explicit diagnostic threshold reported | Model default/NR | Pathology diagnosis of ADC or SCC | Unclear | Follow-up: Not reported \| Not reported | External_test_40_images | ^19^ |
| Teramoto et al., 2017 | ADC/SCC/SCLC subtyping | Probabilities of adenocarcinoma, squamous cell carcinoma, and small cell carcinoma | Softmax maximum class probability; threshold not otherwise reported | Model default | Histopathology and immunohistochemistry diagnosis | Unclear | Follow-up: Not reported \| Not reported | Threefold_CV_original_images | ^20^ |
| Teramoto et al., 2019 | Benign-malignant lung cytology patch and case classification | VGG-16 softmax malignancy probability; case-level maximum patch malignancy probability | Model default/softmax output; ROC analyses also reported; primary row used augmented case-level classification | Model default; threshold not explicitly prespecified | Combined histopathological and immunohistochemical diagnosis; cytopathologist-confirmed patch labels | Unclear | All cases had histopathology/IHC-confirmed benign or malignant diagnosis | Threefold cross-validation with images divided by case; cytopathologist review of patch labels | ^21^ |
| Teramoto et al., 2020 | Benign-malignant lung cytology patch classification | VGG-16 malignancy probability after PGGAN synthetic-image pretraining and actual-image fine-tuning | Malignancy output cutoff 0.5 | Model-default cutoff specified for confusion matrix evaluation | Combined histopathological and immunohistochemical diagnosis; cytopathologist assessment of malignant-cell presence in patches | Unclear | All cases had histopathology/IHC-confirmed benign or malignant diagnosis | Validation images used during training; threefold cross-validation for final classification | ^22^ |
| Teramoto et al., 2022 | ADC/SCC/SCLC subtyping | Softmax probabilities from fine-tuned AlexNet/InceptionV3/VGG16/ResNet50; downstream NB/SVM/RF/NN classifiers used 12 DCNN probability features | Model-default three-class output/argmax; no separate diagnostic threshold reported | Model default | Final diagnosis by histopathology and immunohistochemistry | Unclear | All cases had histopathology/IHC-confirmed ADC, SCC, or SCLC diagnosis | Threefold cross-validation with images from the same case kept in the same dataset | ^23^ |
| Tondo et al., 2025 | Lung cancer-positive vs negative ROSE image classification | Google Inception v3 image embeddings with FCBF feature selection and neural-network classifier; kNN used for doubtful-inclusive analysis | Model default in Orange; positive/negative analysis selected neural network by CA/F1/MCC; doubtful-inclusive analysis selected kNN | Optimized/model-selected in internal 10-fold cross-validation | Definitive histological report, possibly supplemented by immunohistochemistry | Unclear | Gold standard definitive histological diagnosis for the analyzed ROSE images | Retrospective multicentre image set; pathologist contributed to diagnostic classification and training-set refinement | ^24^ |
| Wang et al., 2025a | Malignant vs benign/non-malignant lung cytology image classification and malignant-region segmentation | YOLOv8-SE instance-segmentation output with pixel-level malignant regions and image-level positive-instance rule | Image categorized as malignant if at least one positive instance was detected; explicit probability cutoff not reported | Operational rule prespecified in the model testing section | Postoperative FFPE histopathology gold standard; two pathologists annotated/diagnosed cytology images with adjudication for disagreement | Unclear | Final postoperative FFPE diagnosis used as the gold standard for lung lesion samples | Two pathologists reviewed images; the adjudicating pathologist resolved disagreements; additional SIC, pleural fluid, and BAL test sets were assessed | ^25^ |
| Wang et al., 2025b | Lung cancer vs non-cancer; other cancers vs non-cancer; lung cancer vs other cancers | Class assignment from mid-level fused PCA scores and digitized CEA indicator | LDA threshold 0.5; SVM/KNN model default | Model default / optimized by model training | Histopathologic or cytologic examination confirmed the clinical group | Unclear | Follow-up: Not reported \| Clinical diagnosis and medical record CEA extraction | CV_pairwise_tasks | ^26^ |
| Wilbur et al., 2015 | Lung cancer vs nonmalignant sputum specimen | Automated classifier flags candidate abnormal cells; the specimen is called positive after a manual cytology review confirms abnormal cells | Any abnormal cell detected/confirmed | Prespecified test definition | Known lung malignancy vs nonmalignant/no known pulmonary pathology | Unclear | Follow-up: Last follow-up not uniformly reported \| Hierarchical cytotechnologist/pathologist review of classifier-flagged cells | Clinical_study_91_specimens | ^27^ |
| Xie et al., 2022 | Benign–malignant pleural effusion WSI diagnosis | ResNet18 patch malignancy probability aggregated to WSI-level count rule | Patch probability >0.50; WSI malignant if >10 malignant patches | Model/prespecified rule in paper | Pathological report; 72% with cytology plus matched cell block/IHC and 28% morphology alone | Unclear | Follow-up: Clinical follow-up not included \| Senior cytopathologist checked slides; WSI labels from pathological reports | Test_60_WSIs | ^28^ |
| Yan et al., 2024 | Lung cancer / malignant-cell detection from ROSE cytology | ResNet101 cancer probability for ROSE image; patient diagnosis is positive if any image is malignant | Image threshold 0.5; patient threshold 0.55 with at least one positive image | Model default/selected threshold | Final pathological diagnosis | Unclear | Follow-up: Not applicable \| Labels accepted only when experienced cytopathologist annotation matched final pathological diagnosis | Internal/external image and patient tests | ^29^ |
| Zhang et al., 2026 | Lung cancer vs non-cancer cytological smear Raman spectra; exploratory ADC/SCC/SCLC subtype analysis | CNN-LSTM classification of preprocessed Raman spectra | Model-default binary/multiclass output | Model default | Histopathological confirmation of lung cancer or other diseases | Unclear | Follow-up: Not reported \| No extrapulmonary tumors or pulmonary metastases; individual benign diagnoses undisclosed | Independent_test_190_spectra | ^30^ |
| Zhao et al., 2025a | Benign–malignant and ADC/SCC/SCLC/LCLC subtyping | Logits/probabilities for Benign, LUSC, LUAD, LCLC, SCLC | Five-class model argmax; no numeric probability cutoff reported | Model default / selected by validation accuracy | Final histopathology with clinical/IHC confirmation | Unclear | Follow-up: Not reported \| Histopathology confirmation; manual ROSE comparator also reported | Test_set_Fig4A | ^31^ |
|  |  | Logits/probabilities for Benign, LUSC, LUAD, LCLC, SCLC after ROSE-image/serum-marker cross-attention fusion | Five-class model argmax; no numeric probability cutoff reported; consensus strategy defined prediction-consistent cases as RIOM/RSMM agreement | Model default / selected by validation accuracy | Final histopathology with clinical/IHC confirmation | Unclear | Follow-up: Not reported \| Histopathology confirmation; consensus high-confidence cohort reported, but raw 2×2 counts not provided | Test_set_Fig4B |  |
| Zhao et al., 2025b | Benign–malignant lung cancer diagnosis | Number of LESSEL-predicted ETCs divided by total cells in BAL cytology WSI | LESSEL-predicted ETC% ≥0.2% | Optimized in the discovery cohort, then fixed for validation/external cohorts | Malignant diagnosis by surgical or biopsy histopathology; benign diagnosis by negative pathology/cytology, ≥6-month follow-up, and alternative etiology | Technicians were blinded to clinical information | Follow-up: ≥6 months for benign controls \| Discovery cut-off; same-center and external validation cohorts | Discovery/validation/external cohorts | ^32^ |

WSI, whole-slide image; CNN, convolutional neural network; ML, machine learning; NIR, near-infrared; MPE, malignant pleural effusion; BPE, benign pleural effusion; TB, tuberculosis; HMLCS, hierarchical multi-label classification system; PLS-DA, partial least squares discriminant analysis; SERS, surface-enhanced Raman spectroscopy; YOLO, you only look once; mAP, mean average precision; IoU, intersection over union; IHC, immunohistochemistry; CBAM, convolutional block attention module; LCNEC, large cell neuroendocrine carcinoma; SCLC, small cell lung cancer; ROC, receiver operating characteristic; CLAM-MB, clustering-constrained attention multiple instance learning multi-branch; MIL, multiple instance learning; PCA, principal component analysis; FCM, flow cytometry; QC, quality control; BALF, bronchoalveolar lavage fluid; ANN, artificial neural network; SVM, support vector machine; NC ratio, nuclear-to-cytoplasmic ratio; ADC, adenocarcinoma; SQCC, squamous cell carcinoma; GCN, graph convolutional network; MLP, multilayer perceptron; PGGAN, progressive growing generative adversarial network; NB, naïve Bayes; RF, random forest; NN, neural network; FCBF, fast correlation-based filter; CA, classification accuracy; MCC, Matthews correlation coefficient; kNN, k-nearest neighbor; SE, squeeze-and-excitation; FFPE, formalin-fixed paraffin-embedded; SIC, scratch-imprint cytology; BAL, bronchoalveolar lavage; LDA, linear discriminant analysis; CEA, carcinoembryonic antigen; KNN, k-nearest neighbor; LUSC, lung squamous cell carcinoma; LCLC, large cell lung carcinoma; LESSEL, label-efficient single-cell expression learning; ETC, epithelial tumor cell; ETC%, epithelial tumor cell percentage

## Supplementary Table S12. Meta-analysis grouping plan.

| **Analysis ID** | **Clinical question** | **Target class** | **Population/specimen** | **Primary inclusion rule** | **Subgroup variables** | **Sensitivity analyses** | **Model/threshold rule** | **Planned output** | **Notes** |
| --- | --- | --- | --- | --- | --- | --- | --- | --- | --- |
| A1 | AI for benign-malignant diagnosis | Malignant / lung cancer / MPE | All eligible cytology and cytology-adjacent specimen analyses | One independent test set/model per study, where possible; positive class harmonized to malignant/lung cancer/MPE. | Specimen type; validation design; algorithm family; threshold selection; RoB; image vs spectroscopy/cytometry. | External validation only; low/unclear data leakage only; exclude non-image or non-patient/spectra-level rows. | Primary model/threshold per study from Excel data, Meta_Analysis_Input sheet | Summary sensitivity/specificity; forest plots; HSROC/bivariate model when feasible. | 18 included DTA rows from 17 studies. |
| A2 | AI for ADC vs rest | ADC/LUAD | Malignant-only or all-case subtype datasets as extracted | One-vs-rest 2x2 rows for ADC/LUAD. | Specimen type, multiclass vs binary subtype, NSCLC-only vs all malignant, model family. | Exclude optimized-only thresholds; external validation only if enough studies. | Primary model/threshold per study. | Pooled sensitivity/specificity for ADC vs rest. | 5 included DTA rows from 5 studies. |
| A3 | AI for SCC vs. the rest | SCC/LUSC/SQCC | Malignant-only or all-case subtype datasets as extracted | One-vs-rest 2x2 rows for SCC/LUSC/SQCC. | Specimen type, multiclass vs binary subtype, model family, reference standard. | Exclude non-independent patch-level rows; low RoB only if enough studies. | Primary model/threshold per study. | Pooled sensitivity/specificity for SCC vs rest. | 5 included DTA rows from 5 studies. |
| A4 | AI for SCLC vs. the rest | SCLC | Malignant-only or all-case subtype datasets as extracted | One-vs-rest 2x2 rows for SCLC. | Specimen type, SCLC prevalence, model family, and validation design. | Exclude non-independent patch-level rows; external validation only if enough studies. | Primary model/threshold per study. | Pooled sensitivity/specificity for SCLC vs rest. | 4 included DTA rows from 4 studies. |
| A5 | Exploratory LCNEC vs SCLC subtyping | LCNEC | LCNEC/SCLC cytology-smear evidence | Retained as exploratory non-primary subtype analysis. | Stain/preparation; threshold rule. | None planned unless additional LCNEC studies are available. | Primary stain/model only to avoid double-counting. | Narrative or exploratory accuracy synthesis. | 1 included DTA rows from 1 study. |

AI, artificial intelligence; MPE, malignant pleural effusion; RoB, risk of bias; ADC, adenocarcinoma; LUAD, lung adenocarcinoma; NSCLC, non-small cell lung cancer; SCC, squamous cell carcinoma; LUSC, lung squamous cell carcinoma; SQCC, squamous cell carcinoma; SCLC, small cell lung cancer; LCNEC, large cell neuroendocrine carcinoma; HSROC, hierarchical summary receiver operating characteristic; DTA, diagnostic test accuracy

## Supplementary Table S13. Heterogeneity, subgroup, and sensitivity analysis coding.

| **Variable** | **Coding levels** | **Extracted from** | **Primary/subgroup/sensitivity** | **Rationale** | **Notes** |
| --- | --- | --- | --- | --- | --- |
| Specimen type | EBUS-TBNA; FNAC; sputum; pleural fluid; bronchial washing/brushing; BAL; mixed/unclear; spectroscopy/cytometry adjunct | Excel database, Study methods / Study_Characteristics sheets | Subgroup | Specimen acquisition and preparation affect cellularity, artifacts, and transferability. | Map mixed studies to the dominant test-set specimen when possible. |
| Cytology preparation | Conventional smear; cell block; liquid-based; ThinPrep; cytospin; cell crop; WSI/patch; mixed/NR | Excel database, Study methods / Cytology_preparation sheets | Subgroup | Preparation affects image appearance, stain uptake, and algorithm generalizability. | Use NR where not explicitly reported. |
| Stain/imaging modality | Papanicolaou; Diff-Quik; H&E; Hemacolor; unstained spectroscopy; mixed/NR | Excel database, Study_Characteristics / AI_Model_Characteristics sheets | Subgroup | A staining and imaging device may alter color, texture, and model calibration. | Separate non-image modalities in sensitivity analyses. |
| Algorithm family | CNN; transformer; hybrid; classical ML; ensemble; foundation model; object detection; segmentation-only | Excel database, AI_Model_Characteristics sheets | Subgroup | Architecture may influence performance and generalizability. | Classify by primary diagnostic component. |
| Validation design | Internal split; cross-validation; temporal; external; multi-institutional external | Excel database, AI_Model_Characteristics / DTA sheets | Subgroup/sensitivity | External validation is expected to better approximate real-world performance. | Use Meta_Analysis_Input to select primary rows. |
| Threshold selection | Prespecified; optimized; model default; multiple; unclear | Excel database, Thresholds_Reference / DTA sheets | Sensitivity | Optimized thresholds may inflate diagnostic accuracy. | Exclude or sensitivity-test post-hoc optimized thresholds. |
| Unit of analysis | Patient; specimen; slide/WSI; image; patch; spectrum/cell | Excel database, BM_DTA / Subtyping_DTA sheets | Sensitivity | Non-independent lower-level units may overstate precision. | Prefer patient/specimen/slide rows for primary meta-analysis. |
| Risk of bias | Low; high; unclear | Excel database, QUADAS2_AI sheets | Sensitivity | High-RoB studies may overestimate accuracy. | Use overall RoB and AI data leakage fields. |

EBUS-TBNA, endobronchial ultrasound-guided transbronchial needle aspiration; FNAC, fine-needle aspiration cytology; BAL, bronchoalveolar lavage; WSI, whole-slide image; NR, not reported; H&E, hematoxylin and eosin; CNN, convolutional neural network; ML, machine learning; DTA, diagnostic test accuracy; BM, benign-malignant; QUADAS2, Quality Assessment of Diagnostic Accuracy Studies 2

## Supplementary Table S14. Certainty of evidence profile for DTA outcomes.

| **Question/outcome** | **Studies** | **Participants/specimens** | **Sensitivity summary** | **Specificity summary** | **Risk of bias** | **Indirectness** | **Inconsistency** | **Imprecision** | **Publication bias** | **Certainty** | **Comments** |
| --- | --- | --- | --- | --- | --- | --- | --- | --- | --- | --- | --- |
| Benign-malignant diagnosis | 17 | 3464 | descriptive median 0.898 (range 0.600-1.000); formal pooling pending | descriptive median 0.876 (range 0.667-1.000); formal pooling pending | Serious: most studies have high/unclear RoB and internal validation | Serious: specimen types, units, and imaging/spectroscopy modalities vary | Serious: heterogeneity anticipated across specimen, model, and threshold choices | Serious when events are sparse, or CI widths are large; finalize after meta-analysis | Undetected; small-study and selective-reporting risk possible | Very low to low (provisional) | Certainty is provisional until formal bivariate/HSROC pooling and sensitivity analyses are completed. |
| ADC vs rest | 5 | 908 | descriptive median 0.866 (range 0.720-1.000); formal pooling pending | descriptive median 0.806 (range 0.733-1.000); formal pooling pending | Serious: few studies and mostly internal validation | Serious: one-vs-rest subtype definitions and populations vary | Serious: heterogeneity anticipated across specimen, model, and threshold choices | Serious when events are sparse, or CI widths are large; finalize after meta-analysis | Undetected; small-study and selective-reporting risk possible | Very low to low (provisional) | Certainty is provisional until formal bivariate/HSROC pooling and sensitivity analyses are completed. |
| SCC vs rest | 5 | 908 | descriptive median 0.733 (range 0.517-1.000); formal pooling pending | descriptive median 0.868 (range 0.818-1.000); formal pooling pending | Serious: few studies and mostly internal validation | Serious: SCC/LUSC/SQCC coding and comparator sets vary | Serious: heterogeneity anticipated across specimen, model, and threshold choices | Serious when events are sparse, or CI widths are large; finalize after meta-analysis | Undetected; small-study and selective-reporting risk possible | Very low to low (provisional) | Certainty is provisional until formal bivariate/HSROC pooling and sensitivity analyses are completed. |
| SCLC vs rest | 4 | 871 | descriptive median 0.758 (range 0.600-0.905); formal pooling pending | descriptive median 0.937 (range 0.923-1.000); formal pooling pending | Serious: few studies and sparse SCLC events | Serious: SCLC comparator classes vary | Serious: heterogeneity anticipated across specimen, model, and threshold choices | Serious when events are sparse, or CI widths are large; finalize after meta-analysis | Undetected; small-study and selective-reporting risk possible | Very low to low (provisional) | Certainty is provisional until formal bivariate/HSROC pooling and sensitivity analyses are completed. |

RoB, risk of bias; CI, confidence interval; HSROC, hierarchical summary receiver operating characteristic; ADC, adenocarcinoma; SCC, squamous cell carcinoma; LUSC, lung squamous cell carcinoma; SQCC, squamous cell carcinoma; SCLC, small cell lung cancer

## Supplementary Table S15. Data extraction disagreements and adjudication log.

| **Study ID** | **Field** | **Reviewer 1 value** | **Reviewer 2 value** | **Discrepancy type** | **Final value** | **Adjudicator** | **Status** | **Reference** |
| --- | --- | --- | --- | --- | --- | --- | --- | --- |
| Ai et al., 2022 | All extracted fields | Extracted from the full text of the paper | NR | None recorded | Completed | Auhtors | Resolved | ^1^ |
| Chen et al., 2020 | All extracted fields | Extracted from the full text of the paper | NR | None recorded | Completed | Auhtors | Resolved | ^2^ |
| Chen et al., 2021a | All extracted fields | Extracted from the full text of the paper | NR | None recorded | Completed | Auhtors | Resolved | ^3^ |
| Chen et al., 2021b | All extracted fields | Extracted from the full text of the paper | NR | None recorded | Completed | Auhtors | Resolved | ^4^ |
| Chen et al., 2024 | All extracted fields | Extracted from the full text of the paper | NR | None recorded | Completed | Auhtors | Resolved | ^5^ |
| Ganesh et al., 2022 | All extracted fields | Extracted from the full text of the paper | NR | None recorded | Completed | Auhtors | Resolved | ^6^ |
| Giarnieri et al., 2025 | All extracted fields | Extracted from the full text of the paper | NR | None recorded | Completed | Auhtors | Resolved | ^7^ |
| Gong et al., 2025 | All extracted fields | Extracted from the full text of the paper | NR | None recorded | Completed | Auhtors | Resolved | ^8^ |
| Gonzalez et al., 2020 | All extracted fields | Extracted from the full text of the paper | NR | None recorded | Completed | Auhtors | Resolved | ^9^ |
| Kim et al., 2023 | All extracted fields | Extracted from the full text of the paper | NR | None recorded | Completed | Auhtors | Resolved | ^10^ |
| Kim et al., 2025 | All extracted fields | Extracted from the full text of the paper | NR | None recorded | Completed | Auhtors | Resolved | ^11^ |
| Lai et al., 2025 | All extracted fields | Extracted from the full text of the paper | NR | None recorded | Completed | Auhtors | Resolved | ^12^ |
| Lan et al., 2024 | All extracted fields | Extracted from the full text of the paper | NR | None recorded | Completed | Auhtors | Resolved | ^13^ |
| Lemieux et al., 2023 | All extracted fields | Extracted from the full text of the paper | NR | None recorded | Completed | Auhtors | Resolved | ^14^ |
| Liu et al., 2023 | All extracted fields | Extracted from the full text of the paper | NR | None recorded | Completed | Auhtors | Resolved | ^15^ |
| Taher et al., 2015 | All extracted fields | Extracted from the full text of the paper | NR | None recorded | Completed | Auhtors | Resolved | ^16^ |
| Taher et al., 2013 | All extracted fields | Extracted from the full text of the paper | NR | Publication-year filename mismatch | Completed | Auhtors | Resolved | ^17^ |
| Tanaka et al., 2025 | All extracted fields | Extracted from the full text of the paper | NR | None recorded | Completed | Auhtors | Resolved | ^18^ |
| Tang et al., 2024 | All extracted fields | Extracted from the full text of the paper | NR | No raw confusion matrix | Completed | Auhtors | Resolved | ^19^ |
| Teramoto et al., 2017 | All extracted fields | Extracted from the full text of the paper | NR | None recorded | Completed | Auhtors | Resolved | ^20^ |
| Teramoto et al., 2019 | All extracted fields | Extracted from the full text of the paper | NR | None recorded | Completed | Auhtors | Resolved | ^21^ |
| Teramoto et al., 2020 | All extracted fields | Extracted from the full text of the paper | NR | None recorded | Completed | Auhtors | Resolved | ^22^ |
| Teramoto et al., 2022 | All extracted fields | Extracted from the full text of the paper | NR | papers filename/first-author mismatch | Completed | Auhtors | Resolved | ^23^ |
| Tondo et al., 2025 | All extracted fields | Extracted from the full text of the paper | NR | AUC inconsistency noted | Completed | Auhtors | Resolved | ^24^ |
| Wang et al., 2025a | All extracted fields | Extracted from the full text of the paper | NR | Counts approximate | Completed | Auhtors | Resolved | ^25^ |
| Wang et al., 2025b | All extracted fields | Extracted from the full text of the paper | NR | Non-independent spectra-level data | Completed | Auhtors | Resolved | ^26^ |
| Wilbur et al., 2015 | All extracted fields | Extracted from the full text of the paper | NR | None recorded | Completed | Auhtors | Resolved | ^27^ |
| Xie et al., 2022 | All extracted fields | Extracted from the full text of the paper | NR | Counts reconstructed | Completed | Auhtors | Resolved | ^28^ |
| Yan et al., 2024 | All extracted fields | Extracted from the full text of the paper | NR | Non-independent image rows | Completed | Auhtors | Resolved | ^29^ |
| Zhang et al., 2026 | All extracted fields | Extracted from the full text of the paper | NR | Spectrum-level only | Completed | Auhtors | Resolved | ^30^ |
| Zhao et al., 2025a | All extracted fields | Extracted from the full text of the paper | NR | Fig. 4B metric discrepancy | Completed | Auhtors | Resolved | ^31^ |
| Zhao et al., 2025b | All extracted fields | Extracted from the full text of the paper | NR | No subtype output | Completed | Auhtors | Resolved | ^32^ |

## Supplementary Table S16. Data coding definitions and formulae.

| **Item** | **Definition/formula** | **Coding note** |
| --- | --- | --- |
| TP | Index test positive and reference standard positive. | For benign-malignant analysis, malignant is usually the positive class. |
| FP | The index test is positive, and the reference standard is negative. | For subtype one-vs-rest, the non-target is predicted as the target. |
| FN | The index test was negative, and the reference standard was positive. | For subtype one-vs-rest, the target is predicted as non-target. |
| TN | The index test and the reference standard were negative. | For subtype one-vs-rest, the non-target is predicted as non-target. |
| Sensitivity | TP / (TP + FN). | Report or calculate per independent test set. |
| Specificity | TN / (TN + FP). | Report or calculate per independent test set. |
| Positive likelihood ratio | Sensitivity / (1 - specificity). | Calculated in the Excel workbook for checking when applicable. |
| Negative likelihood ratio | (1 - sensitivity) / specificity. | Calculated in the Excel workbook for checking when applicable. |
| Diagnostic odds ratio | (TP x TN) / (FP x FN). | Workbook uses continuity-corrected cells for transformed metrics when needed. |
| Zero-cell continuity correction | Add 0.5 to all four cells only for transformed/checking metrics when any cell is zero. | Keep raw TP, FP, FN, TN unchanged for primary DTA software input. |
| Independent dataset | A dataset/test set in which participants/specimens do not overlap with another included row. | Avoid double-counting multiple models or thresholds from the same test set. |

TP, true positive; FP, false positive; FN, false negative; TN, true negative; DTA, diagnostic test accuracy

# Reference

1.	Ai D, Hu Q, Chao Y-C, et al. Artificial intelligence-based rapid on-site cytopathological evaluation for bronchoscopy examinations. *Intell-Based Med*. 2022;6:100069. doi:10.1016/j.ibmed.2022.100069

2.	Widodo R, Badriyah T, Syarif I. Detection of lung cancer cell based on cytological examination using convolutional neural network. *Klik-Kumpul J Ilmu Komput*. 2020;7(3):296.

3.	Lin CK, Chang J, Huang CC, Wen YF, Ho CC, Cheng YC. Effectiveness of convolutional neural networks in the interpretation of pulmonary cytologic images in endobronchial ultrasound procedures. *Cancer Med*. 2021;10(24):9047–9057. doi:10.1002/cam4.4383

4.	Chen Z, Chen K, Lou Y, Zhu J, Mao W, Song Z. Machine learning applied to near-infrared spectra for clinical pleural effusion classification. *Sci Rep*. 2021;11(1):9411. doi:10.1038/s41598-021-87736-4

5.	Chen J, Zhang C, Xie J, et al. Automatic lung cancer subtyping using rapid on-site evaluation slides and serum biological markers. *Respir Res*. 2024;25(1):391. doi:10.1186/s12931-024-03021-8

6.	Perumal J, Lee P, Dev K, Lim HQ, Dinish U, Olivo M. Machine learning assisted real-time label-free SERS diagnoses of malignant pleural effusion due to lung cancer. *Biosensors*. 2022;12(11):940. doi:10.3390/bios12110940

7.	Giarnieri E, Carico E, Scarpino S, et al. Bringing AI to clinicians: Simplifying pleural effusion cytology diagnosis with user-friendly models. *Diagnostics*. 2025;15(10):1240. doi:10.3390/diagnostics15101240

8.	Gong W, Vaishnani DK, Jin X-C, et al. Evaluation of an enhanced ResNet-18 classification model for rapid On-site diagnosis in respiratory cytology. *BMC Cancer*. 2025;25(1):10. doi:10.1186/s12885-024-13402-3

9.	Gonzalez D, Dietz RL, Pantanowitz L. Feasibility of a deep learning algorithm to distinguish large cell neuroendocrine from small cell lung carcinoma in cytology specimens. *Cytopathology*. 2020;31(5):426–431. doi:10.1111/cyt.12829

10.	Kim T, Chang H, Kim B, et al. Deep learning-based diagnosis of lung cancer using a nationwide respiratory cytology image set: improving accuracy and inter-observer variability. *Am J Cancer Res*. 2023;13(11):5493.

11.	Kim D, Lee J, Jung M, et al. Whole slide image-level classification of malignant effusion cytology using clustering-constrained attention multiple instance learning. *Lung Cancer*. 2025;204:108552. doi:10.1016/j.lungcan.2025.108552

12.	Lai C-L, Lee K-H, Nguyen H-T, et al. Visible-light hyperspectral reconstruction and PCA-based feature extraction for malignant pleural effusion cytology. *Biosensors*. 2025;15(11):714. doi:10.3390/bios15110714

13.	Lan H, Chen P, Wang C, et al. A multiscale connected UNet for the segmentation of lung cancer cells in pathology sections stained using rapid on-site cytopathological evaluation. *Am J Pathol*. 2024;194(9):1712–1723. doi:10.1016/j.ajpath.2024.05.011

14.	Lemieux ME, Reveles XT, Rebeles J, et al. Detection of early-stage lung cancer in sputum using automated flow cytometry and machine learning. *Respir Res*. 2023;24(1):23. doi:10.1186/s12931-023-02327-3

15.	Liu Z, Zhang J, Wang N, et al. Enhanced YOLOv5 network-based object detection (BALFilter Reader) promotes PERFECT filter-enabled liquid biopsy of lung cancer from bronchoalveolar lavage fluid (BALF). *Microsyst Nanoeng*. 2023;9(1):121. doi:10.1038/s41378-023-00580-6

16.	Taher F, Werghi N, Al-Ahmad H. Computer aided diagnosis system for early lung cancer detection. *Algorithms*. 2015;8(4):1088–1110. doi:10.3390/a8041088

17.	Taher F, Werghi N, Al-Ahmad H. Automatic sputum color image segmentation for lung cancer diagnosis. *KSII T Internet Info*. 2013;7(1)doi:10.3837/tiis.2013.01.005

18.	Tanaka R, Tsuboshita Y, Okodo M, et al. Artificial intelligence recognition model using liquid-based cytology images to discriminate malignancy and histological types of non-small-cell lung cancer. *Pathobiology*. 2025;92(1):52–62. doi:10.1159/000541148

19.	Tang S, Geng C, Gong W, Hu F, Dai Y. Dual-branch knowledge fusion network for differential diagnosis of lung cancer cytopathology images. *J Shanghai Jiaotong Univ*. 2024:1–11. doi:10.1007/s12204-024-2783-3

20.	Teramoto A, Tsukamoto T, Kiriyama Y, Fujita H. Automated classification of lung cancer types from cytological images using deep convolutional neural networks. *Biomed Res Int*. 2017;2017(1):4067832. doi:10.1155/2017/4067832

21.	Teramoto A, Yamada A, Kiriyama Y, et al. Automated classification of benign and malignant cells from lung cytological images using deep convolutional neural network. *Inform Med Unlocked*. 2019;16:100205. doi:10.1016/j.imu.2019.100205

22.	Teramoto A, Tsukamoto T, Yamada A, et al. Deep learning approach to classification of lung cytological images: Two-step training using actual and synthesized images by progressive growing of generative adversarial networks. *PLoS One*. 2020;15(3):e0229951. doi:10.1371/journal.pone.0229951

23.	Tsukamoto T, Teramoto A, Yamada A, et al. Comparison of fine-tuned deep convolutional neural networks for the automated classification of lung cancer cytology images with integration of additional classifiers. *Asian Pac J Cancer Prev*. 2022;23(4):1315. doi:10.31557/APJCP.2022.23.4.1315

24.	Tondo P, Palmiotti GA, D'Alagni G, et al. Reliability of rapid on‑site evaluation achieved by remote sharing systems (E‑ROSE) and AI algorithms (AI‑ROSE) compared with the gold standard in the diagnosis of lung cancer. *Respirology*. 2025;30(12):1184–1191. doi:10.1111/resp.70104

25.	Wang Q, Luo Y, Zhao Y, et al. Automated recognition and segmentation of lung cancer cytological images based on deep learning. *PLoS One*. 2025;20(1):e0317996. doi:10.1371/journal.pone.0317996

26.	Wang L, Hong W, Fan D, et al. Mid-level data fusion of pleural effusion SERS spectra and serum CEA levels using machine learning algorithms for precise lung cancer detection. *Nanoscale*. 2025;17(27):16349–16360. doi:10.1039/d5nr01405k

27.	Wilbur DC, Meyer MG, Presley C, et al. Automated 3‐dimensional morphologic analysis of sputum specimens for lung cancer detection: performance characteristics support use in lung cancer screening. *Cancer Cytopathol*. 2015;123(9):548–556. doi:10.1002/cncy.21565

28.	Xie X, Fu C-C, Lv L, et al. Deep convolutional neural network-based classification of cancer cells on cytological pleural effusion images. *Mod Pathol*. 2022;35(5):609–614. doi:10.1038/s41379-021-00987-4

29.	Yan S, Li Y, Pan L, Jiang H, Gong L, Jin F. The application of artificial intelligence for Rapid On-Site Evaluation during flexible bronchoscopy. *Front Oncol*. 2024;14:1360831. doi:10.3389/fonc.2024.1360831

30.	Zhang Z, Zhang S, Luo H, et al. Label-free Raman spectroscopy combined with CNN-LSTM model for rapid diagnosis of lung cancer based on cytological smears. *Vib Spectrosc*. 2026:103914. doi:10.1016/j.vibspec.2026.103914

31.	Zhao Z, Zou Q, Fang Y, et al. Integration of ROSE cytology and serum tumor markers for rapid subtyping of lung cancer. *Sci Rep*. 2025;doi:10.1038/s41598-025-27770-8

32.	Zhao Y, Qiu R, Wang Z, et al. Single-cell sequencing-guided annotation of rare tumor cells for deep learning-based cytopathologic diagnosis of early lung cancer. *Adv Sci*. 2025;12(22):2416921. doi:10.1002/advs.202416921
